# Gene Network Inference and Biochemical Assessment Delineates GPCR Pathways and CREB Targets in Small Intestinal Neuroendocrine Neoplasia

**DOI:** 10.1371/journal.pone.0022457

**Published:** 2011-08-11

**Authors:** Ignat Drozdov, Bernhard Svejda, Bjorn I. Gustafsson, Shrikant Mane, Roswitha Pfragner, Mark Kidd, Irvin M. Modlin

**Affiliations:** 1 Cardiovascular Division, King's College London BHF Centre of Research Excellence, James Black Centre, London, United Kingdom; 2 Centre for Bioinformatics, School of Physical Sciences and Engineering, King's College London, London, United Kingdom; 3 Gastrointestinal Pathobiology Research Group, Yale University School of Medicine, New Haven, Connecticut, United States of America; 4 Department of Gastroenterology, St Olavs Hospital, and Department of Cancer Research and Molecular Medicine, NTNU, Trondheim, Norway; 5 Keck Affymetrix Facility, Yale University School of Medicine, New Haven, Connecticut, United States of America; 6 Institute of Pathophysiology and Immunology, Centre for Molecular Medicine, Medical University of Graz, Austria; Beth Israel Deaconess Medical Center, United States of America

## Abstract

Small intestinal (SI) neuroendocrine tumors (NET) are increasing in incidence, however little is known about their biology. High throughput techniques such as inference of gene regulatory networks from microarray experiments can objectively define signaling machinery in this disease. Genome-wide co-expression analysis was used to infer gene relevance network in SI-NETs. The network was confirmed to be non-random, scale-free, and highly modular. Functional analysis of gene co-expression modules revealed processes including ‘Nervous system development’, ‘Immune response’, and ‘Cell-cycle’. Importantly, gene network topology and differential expression analysis identified over-expression of the GPCR signaling regulators, the cAMP synthetase, *ADCY2*, and the protein kinase A, *PRKAR1A*. Seven CREB response element (CRE) transcripts associated with proliferation and secretion: *BEX1*, *BICD1*, *CHGB*, *CPE*, *GABRB3*, *SCG2* and *SCG3* as well as *ADCY2* and *PRKAR1A* were measured in an independent SI dataset (*n* = 10 NETs; *n* = 8 normal preparations). All were up-regulated (*p*<0.035) with the exception of *SCG3* which was not differently expressed. Forskolin (a direct cAMP activator, 10^−5^ M) significantly stimulated transcription of pCREB and 3/7 CREB targets, isoproterenol (a selective ß-adrenergic receptor agonist and cAMP activator, 10^−5^ M) stimulated pCREB and 4/7 targets while BIM-53061 (a dopamine D_2_ and Serotonin [5-HT_2_] receptor agonist, 10^−6^ M) stimulated 100% of targets as well as pCREB; CRE transcription correlated with the levels of cAMP accumulation and PKA activity; BIM-53061 stimulated the highest levels of cAMP and PKA (2.8-fold and 2.5-fold vs. 1.8–2-fold for isoproterenol and forskolin). Gene network inference and graph topology analysis in SI NETs suggests that SI NETs express neural GPCRs that activate different CRE targets associated with proliferation and secretion. *In vitro* studies, in a model NET cell system, confirmed that transcriptional effects are signaled through the cAMP/PKA/pCREB signaling pathway and that a SI NET cell line was most sensitive to a D_2_ and 5-HT_2_ receptor agonist BIM-53061.

## Introduction

Neuroendocrine or “carcinoid” tumors of the gut, usually misperceived as a rare, indolent neoplasia, have not rigorously been studied, are poorly understood and often misdiagnosed [1]. The perception that these tumors are rare has been altered by introduction of diagnostic strategies including endoscopy, the measurement of plasma biochemical markers such as Chromogranin A, and nuclear medicine techniques, including somatostatin receptor scintigraphy (SRS) [2]. A review of the current Surveillance Epidemiology and End Results (SEER) database indicates that small intestinal (SI) neuroendocrine tumors (NET) comprise 24.3% of all NETs with the overall 5-year survival rate of 64.1% [3], [4]. In the event of liver metastases, bioactive tumor products enter the systemic circulation, bypassing hepatic inactivation, and engender a ‘carcinoid syndrome’. This consists of a variety of symptoms including episodic skin flushing, diarrhea, bronchoconstriction, sweating and abdominal cramping, and as many as 30–50% of individuals may have cardiac valvular disease [5].

Although the cell of origin of SI NET has been identified as the enterochromaffin (EC) cell, the secretory and proliferative regulation of these cells is poorly defined and, as a result, progress in the development of effective therapeutic strategies for diseases associated with the cell, e.g. NETs or Crohns disease [6], has been limited. The principal secretory product of the EC cell is serotonin (5-HT), although substance P (motility regulator) and guanylin (secretory regulator) have also been identified [7]–[9]. The most successful therapy, to date, has been somatostatin analogs which activate inhibitory G-protein coupled receptors (GPCRs) and result in decreased secretion of bioactive products with concomitant amelioration of symptoms [10]–[12].

GPCRs represent the largest family of cell-surface molecules involved in environment sensing and signal transmission, accounting for >2% of the total genes encoded by the human genome [13]. Mutations in GPCRs and G_α_ subunits have been identified in endocrine tumors and are often associated with symptoms caused by unregulated hormonal secretion. For example, activating mutations of the thyroid stimulating hormone receptor (TSHR) are found in some thyroid carcinomas and approximately 80% of thyroid adenomas, while germline mutations in TSHR cause familial non-autoimmune hyperthyroidism [14]. In the GPCR-mediated downstream signal transduction system, cyclic AMP responsive element-binding (CREB) protein has been shown to be an important transcription factor that is involved in the progression of hepatocellular carcinoma, leukemia, pituitary tumor, and lung cancer through control of cell function (secretion, proliferation, angiogenesis and apoptosis) [15]–[17]. To date, the cAMP/CREB mechanism in SI NETs has not been demonstrated.

In our previous evaluation of transcriptome analyses (Affymetrix U133 Plus chips) of the normal human EC cell and GI NET cell line KRJ-I, we identified candidate luminal GPCRs and neural/hormonal GPCRs including β1 adrenergic and dopamine D receptors (DR) [18], [19]. Further investigation demonstrated that isoproterenol, a ß-adrenergic GPCR agonist, stimulated 5-HT secretion through increased intracellular cAMP [20]. Others have shown, in PC12 (rat pheochromocytoma cells), HEK293T (Human Embryonic Kidney cells) and the pancreatic beta cell line, MIN6, that activation of the cAMP pathway stimulates gene expression through protein kinase A (PKA)-mediated phosphorylation of CREB at Ser-133 [21], [22]. Since little is known about neoplastic EC cell transcription and proliferation or secretion, we considered that delineation of the molecular basis of GPCR-mediated transcription through cAMP/PKA/CREB would provide novel information regarding the mechanistic basis of these processes and facilitate the identification of new therapeutic targets that might be used to inhibit NET function. As these tumors autoregulate their own growth through amine production [23] and regulate the local microenvironment (e.g. stimulate fibroblast proliferation and secretion) [24], delineating GPCR-pathways may identify novel targets to inhibit tumor cell proliferation.

We used gene network analysis and identified *in silico* the cAMP/CREB-mediated mechanisms of transcription in SI NETs. Using an established SI NET model, the human EC cell line (KRJ-I) [25], we validated GPCR-mediated transcription of CREB targets through cAMP/PKA/pCREB-activation in SI NETs. Our results provide novel information regarding the transcription of CREB response elements (CREs) known to be relevant to tumor proliferation and secretion that are activated by GPCR regulation of intracellular cAMP. Furthermore, we offer the first formal network topology analysis of this disease.

## Results

### 1. Systems-wide properties of a SI NET gene co-expression network

Gene co-expression patterns reflecting the pathogenesis of SI NETs were represented as undirected weighted network where nodes correspond to genes and edges correspond to co-expressions between them. We examined the network by systematically testing the Pearson correlation coefficient (PCC) cut-off in the range from 0.5 to 1 (**Figure S1**). Only gene pairs with an absolute PCC≥0.94 were included in the network. The reasons for selecting such stringent cut-off (0.94) were three-fold: i) gene correlation profiles with PCC over 0.60 are known to be more biologically relevant [26], ii) at PCC<0.94 the network was excessively large (most of the nodes were present), suggesting evidence of false-positive edges, iii) at PCC≥0.94, a large number of connected components emerged, while the overall network density remained high, suggesting that genes were organized into tightly interconnected modules that may be functionally relevant (**Figure S1**). The final network contained 3470 genes and 4549 links (average node degree = 2.6). Of these, 788 (23%) genes have known tumorigenic somatic mutations (obtained from the Catalogue of Somatic Mutations in Cancer [COSMIC] database).

**Figure 1:**
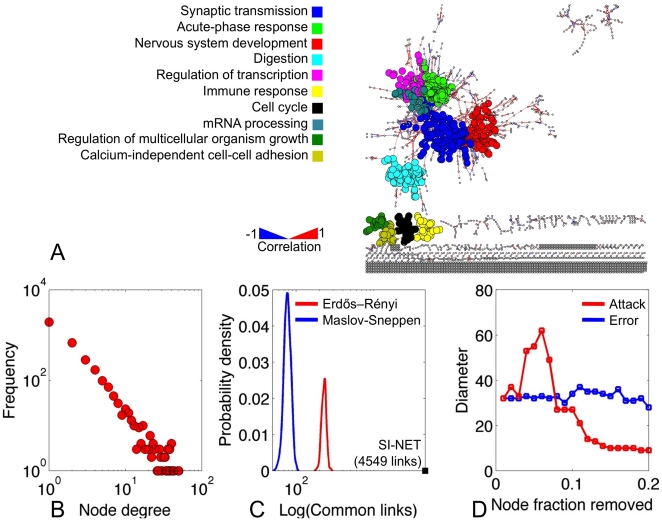
Network analysis of the SI NET interactome. **1A**) The SI NET network and top 10 functional gene clusters identified using the Louvain algorithm and enriched for Gene Ontology (GO) Biological Process (BP) terms. **1B**) Node degree frequency distribution for the SI NET gene co-expression network. **1C**) Comparison of the SI NET network to random graph models generated by using the Erdős–Rényi and Maslov-Sneppen algorithms. **1D**) Change in stability of the SI NET network following the removal of highly connected genes (attack) and random genes (error).

It has been suggested that, in a co-expression network, functionally related genes tend to organize into tightly linked communities [27]. The Louvain algorithm was used to identify these functional modules in the SI NET network in an unbiased manner (see **Methods**). The network was partitioned into 882 clusters, of which 10 contained >20 genes. Network modularity was 0.86, confirming that the SI NET interactome is embedded with highly interconnected modules, reinforcing the complex nature of signaling cascades in this disease (**Figure 1A**). The top 10 clusters were enriched for Gene Ontology (GO) Biological Process (BP) terms. The most enriched terms included ‘Nervous system development’ (BEX1, SYN1, GRIA2), ‘Immune response’ (CD38, IGKC, SLAMF8), and ‘Cell cycle’ (ASPM, MKI67, TOP2A).

To determine the overall architecture of the SI NET interactome, the node degree frequency distribution was calculated and established to be “scale-free” (**Figure 1B**). Generally, a scale-free architecture implies that most of the connections are confined to a few highly interconnected nodes (hubs) – a hallmark of most biological networks. However, it is possible to reconstruct similar connectivity patterns using random edge rewiring. To confirm that the SI NET network was non-random, we compared it to two models of random networks (see **Methods**): the Maslov-Sneppen model (scale-free architecture, preserved node degrees, randomly rewired edges) and the Erdős–Rényi model (preserved number of nodes, edges are constructed using a random Gaussian probability distribution). For each model, 200 random networks were generated and intersected with the original SI NET graph. On average, the Maslov-Sneppen and the Erdős–Rényi networks shared 78.3 (standard deviation = 7.7) and 229.1 (standard deviation = 15.3) links with the SI NET interactome respectively (**Figure 1C**). This substantiates the hypothesis that the original network is significantly non-random (minimal z-score = 282.3).

To assess the stability of the SI NET network, we measured the effects of random (error) and targeted (attack) node removal on the network diameter. Removal of random nodes had no effect on the diameter, suggesting that the SI NET interactome was robust against random mutations. However, targeted removal of the most connected hubs, as predicted, caused the network to collapse (**Figure 1D**).

### 2. *In Silico* Prediction of CREB Targets

Interestingly, 940/3470 genes (27%) in the SI NET network were differentially expressed (student's t-test, p≤0.05). Of these, 539 were up-regulated and 401 were down-regulated compared to normal SI mucosa. Eight genes (CHGA, CPE, ENO2, INSM1, PTPRN2, SERPINA10, and SLC18A1/2) that have been previously identified as markers of neuroendocrine tumors [28]–[31] were confirmed in this study to be altered. Automated KEGG pathway analysis (using the DAVID Functional Annotation Database) of differentially expressed genes, identified an over-represented (*p* = 0.03) GPCR signaling pathway characterized by an over-expression of cyclic AMP synthetase and adenylate cyclase 2 (ADCY2, *t-value* = 6.0) and ADCY9 (t-value = -3.8), and a PKA responsible for phosphorylation of the CREB transcription factor, PRKAR1A (t-value = 2.8) (**Figure S2**). The Wnt signaling pathway was also identified. These findings are consistent with previous studies *in vitro* of small intestinal and pituitary NET cell lines investigating cAMP recruitment through a GPCR complex and downstream elements of CREB phosphorylation [20], [32]–[36].

**Figure 2:**
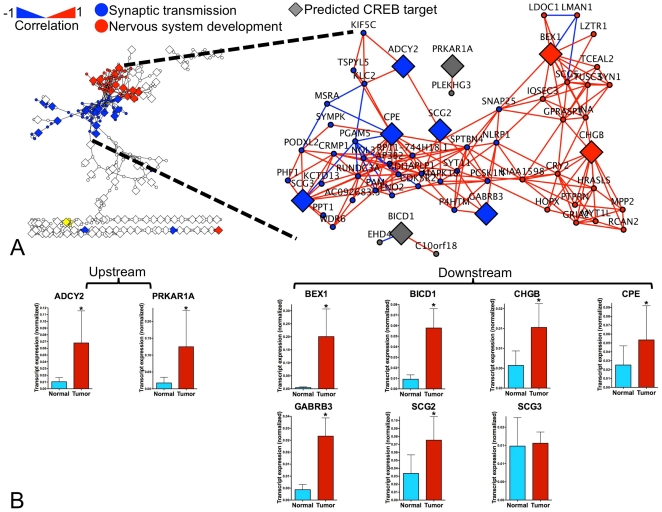
*ADCY2*, *PRKAR1A*, and CREB response elements expression examined by Real-time PCR. **2A**) Identification of putative CREB response elements in the SI NET interactome. **2B**) Transcripts of *ADCY2*, cAMP synthetase, and *PRKAR1A*, a key member of the PKA, were up-regulated in KRJ-I, (700% and 722% respectively, *p*<0.02 compared to normal). Six of 7 CRE transcripts were confirmed to be over-expressed in SI NETs (*p*<0.05) with the exception of *SCG3* (*p* = 0.24). MEAN±SEM (n_Tumor_ = 10, n_Normal_ = 8).

We next compared up-regulated genes to a CREB Target Gene database (http://natural.salk.edu/CREB/) to identify potential CREs in SI NETs. Using a confidence level for the binding value (BV)<0.001 and a binding ratio (BR)>1.5, which are considered to be significant for the identification of CREs [22], a list of 123 genes representing putative CREB binding targets and cAMP response elements was compiled. Interestingly, the putative CREs localized only to the ‘Synaptic transmission’ and ‘Nervous system development’ clusters of the SI NET interactome, which is consistent with the biological function of known CREB targets as well as the nature of the neuroendocrine system [37] (**Figure 2A**). We specifically selected for further investigation the CREs (BEX1, BICD1, CHGB, CPE, GABRB3, SCG2, and SCG3) given their known association with the regulation of cell function [16], [29], [30], [38]–[40] (**Table 1**).

**Table 1:** 7 CREB targets assessed by Real-time PCR in a SI NET database and the KRJ-I cell line.

Gene Symbol	Gene Title	CREB p-value	CREB binding ratio	GO Biological Process	Chromosomal Location
BEX1	brain expressed, X-linked 1	5.00E-05	2.1	multicellular organismal development	Xq21-q23
				nervous system development	
				cell differentiation	
BICD1	bicaudal D homolog 1 (Drosophila)	1.30E-03	1.7	RNA processing	12p11.2-p11.1
				intracellular mRNA localization	
				anatomical structure morphogenesis	
CHGB	chromogranin B (secretogranin 1)	7.50E-08	3	—	20pter-p12
CPE	carboxypeptidase E	1.10E-12	7.4	protein modification process	4q32.3
				proteolysis	
				neuropeptide signaling pathway	
				metabolic process	
				insulin processing	
GABRB3	gamma-amino[10]butyric acid (GABA) A receptor, beta 3	1.20E-02	1.5	Transport	15q11.2-q12
				signal transduction	
SCG2	secretogranin II (chromogranin C)	1.70E-04	1.9	MAPKKK cascade	2q35-q36
				Angiogenesis	
				regulation of endothelial cell proliferation	
				cell motility	
				inflammatory response	
				intracellular signaling cascade	
				protein secretion	
SCG3	secretogranin III	2.50E-05	2.2	—	15q21

### 3. Real-time PCR Validation of the Gene Expression Analysis

To confirm the over-expression of CRE transcripts in SI NETs, we measured transcript expression by real-time PCR (RT-PCR) of *n* = 7 CREB targets (**Table 1**) in the SI NET cell line KRJ-I (*n* = 10) and in normal EC cell preparations (*n* = 8) (**Figure 2B**). Transcript levels were normalized to expression of housekeeping genes *ALG9*, *TFCP2*, and *ZNF410* as described [41] using GeNorm [42]. Levels of *BEX1*, *BICD1*, *CHGB*, *CPE*, *GABRB*3 and *SCG2* were up-regulated in SI NETs (*p*<0.05) while Secretogranin III (*SCG3*) was not differently expressed, *p* = 0.24 (**Figure 2B**).

Additionally, we measured transcription of *ADCY2* and *PRKAR1A*. Levels of these upstream CRE pathway regulators were elevated 700% and 722% respectively, in KRJ-I (*p* = 0.02) compared to normal EC cells (**Figure 2B**).

### 4. *In vitro* model of CRE transcription

**Figure 3:**
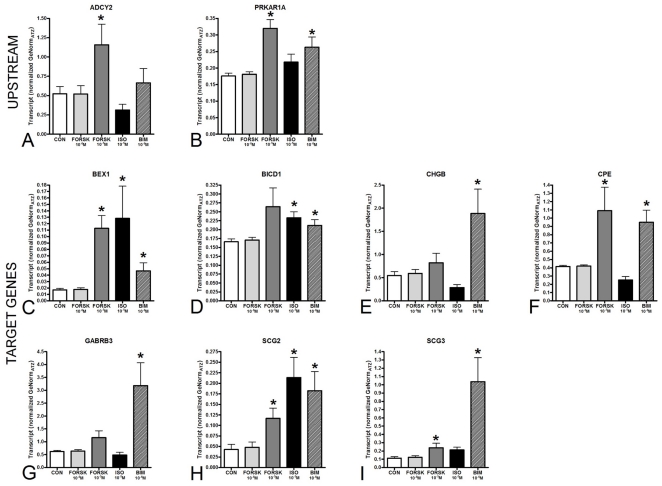
*In vitro* assessment of *ADCY2*, *PRKAR1A*, and CREB response elements transcripts. *ADCY2* responded to cAMP activator forskolin (10^−5^ M: 220%) (**3A**), while *PRKAR1A* was stimulated by forskolin and dopamine D_2_ and 5-HT_2_ receptor agonist BIM-53061 (10^−6^ M) (188% and 153% respectively) (**3B**). BIM-53061 was a universal CRE activator, while forskolin had a less pronounced effect (**3C–I**). The selective ß-adrenergic receptor agonist isoproterenol (10^−5^ M) stimulated transcription of *BEX1*, *BICD1*, *SCG2* and *SCG3*. Forskolin (10^−6^ M) had no effect. *p<0.05 vs. CON. MEAN±SEM (*n* = 6).

To evaluate whether these genes were regulated through the cAMP signaling pathway *in vitro*, we investigated their expression in KRJ-I. CRE transcription was measured by stimulating KRJ-I cells with the cAMP activator forskolin (10^−6^ M and 10^−5^ M), the selective β-adrenergic receptor agonist isoproterenol (10^−5^ M), and the dopamine D_2_ (D_2_R) and Serotonin (5-HT) receptor agonist BIM-53061 (10^−6^ M) (**Figure 3**) for two hours. While *ADCY2* was significantly upregulated only by forskolin (10^−5^ M, 220%, *p*<0.03), *PRKAR1A* transcripts were up-regulated both by forskolin and BIM-53061 (188%, *p* = 0.0004, and 153%, *p* = 0.021 respectively). Lower concentrations of forskolin (10^−6^ M) did not stimulate transcription of either *ADCY2* or *PRKAR1A*. Overall, BIM-53061 was a universal CRE activator (100% of target genes transcriptionally activated), while the effects of forskolin (10^−5^ M, ∼42% activated) and isoproterenol (∼57% activated) were less pronounced.

### 5. cAMP/PKA and pCREB activation *in vitro*

**Figure 4:**
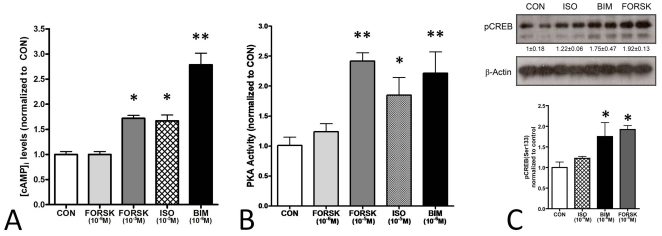
Functional assessment of the cAMP pathway in the KRJ-I cell line. **4A**) Intracellular cAMP accumulation in KRJ-I cell line. Stimulation with forskolin (10^−5^ M), isoproterenol (10^−5^ M) or BIM-53061 (10^−6^ M) increased cAMP accumulation *in vitro* 72%, 67%, and 178% respectively. Forskolin at lower concentrations (10^−6^ M) had no effect. *p<0.05, **p = 0.006 vs CON. MEAN±SEM (*n* = 3). **4B**) PKA activity in the KRJ-I cell line. Stimulation with forskolin (10^−5^ M), isoproterenol (10^−5^ M) or BIM-53061 (10^−6^ M) increased PKA activity *in vitro* 142%, 85%, and 122% respectively. Forskolin at lower concentrations (10^−6^ M) had no effect. *p<0.05, **p<0.01 vs. CON. MEAN±SEM (*n* = 4). **4C**) Phospho-CREB(Ser133) activation in KRJ-I cell line. Stimulation with isoproterenol (10^−5^), BIM-53061 (10^−6^ M) or forskolin (10^−5^ M) increased CREB phosphorylation at the Ser133 site after 15 mins by 122%, 175% and 192% respectively. *p<0.05 vs. CON. MEAN±SEM (*n* = 3).

Next, to confirm that the mechanisms regulating CRE transcription occurred through the cAMP signaling pathway, we measured cAMP in response to each of the ligands in the KRJ-I cell line. Twenty minute incubation with forskolin (10^−5^ M), isoproterenol (10^−5^ M) or BIM-53061 (10^−6^ M) all increased intracellular cAMP accumulation 1.72-fold, 1.67-fold, and 2.78-fold respectively compared to control (*p*<0.05). Lower concentrations of forskolin (10^−6^ M) had no effect on cAMP accumulation (**Figure 4A**). PKA activity was similarly stimulated by these agents in the order of BIM-53061 = forskolin (10^−5^ M) (2.2–2.4-fold)>isoproterenol (1.85-fold) (**Figure 4B**), as was pCREB (BIM-53061 = forskolin (10^−5^ M) (1.75–1.9-fold)>isoproterenol (1.2-fold) (**Figure 4C**).

## Discussion

The role of the cAMP signaling pathway in the regulation of tumor CREB-mediated transcription has not previously been investigated in gastrointestinal NETs. In the current study, using a transcript database of SI NETs and normal SI mucosa we demonstrated: 1) transcripts of *ADCY2*, a member of the adenylate cyclase family, are up-regulated in SI NETs and KRJ-I; 2) intracellular accumulation of cAMP is stimulated by forskolin, isoproterenol and BIM-53061; 2) intracellular PKA activity and pCREB is stimulated by these agents; 4) cAMP-dependent protein kinase, *PRKAR1A*, is over-expressed in SI NETs and KRJ-I cells; 5) CREs are differentially transcribed when subject to a classic cAMP activator, a selective ß-adrenergic receptor agonist, or a selective D_2_R and 5-HT receptor agonist. Additionally, we performed the first formal large scale network topology assessment of SI NET disease.

Initially, using gene network inference, we reconstructed the SI NET co-expression network from genome wide expression levels obtained from microarray profiling. The network (**Table S1**) was determined to be scale-free, non-random, and topologically stable. This suggests a system that is dominated by a few highly connected biologically relevant hubs and that is “protective” against random perturbations (e.g. mutations). Indeed, this is consistent with the behavior of most cellular networks [43] and confirms that the biology of SI NETs can be further probed using graph-theory approaches. The advantage of this approach is that it allows the study of significant genes in relation to the entire system rather than by merits of up- or down-regulation alone. For example, node betweenness centrality as well as node degree have been previously reported as possible indicators of gene essentiality [44]. We computed these statistics for every gene in the SI NET network. Thus our dataset can further be explored using functional assays utilizing network topology as well as differential expression.

Highly modular structure of the SI NET network was explored using an unbiased graph clustering technique (the Louvain algorithm). The method is a greedy optimization method that attempts to optimize the modularity [45] of a partition of the network. We identified 10 modules (>20 genes) in the SI NET that were subsequently enriched for GO-BP terms including ‘Nervous system development’ and ‘Cell cycle’. The functional cluster heterogeneity suggests that the SI NET disease is a multi-modal entity with complex metabolic, hormonal, and proliferative cascades that call for a systems-wide assessment as well as traditional approaches.

We used differential expression analysis to map significantly changed genes onto the SI NET network to increase the biological utility of the analysis. Most of the significantly changed genes formed tight networks involved in transcription, secretion, cell proliferation, tissue development, embryonic development and extracellular matrix regulation. Using the DAVID functional annotation tool [46], it was determined that cAMP/CREB signaling cascade was highly upregulated in SI NETs (**Figure S2**). It was of interest to note that the statistical enrichment also identified that the Wnt signaling pathway was similarly altered. It was previously suggested that the cAMP/CREB signaling may also contribute to Wnt-regulated processes in cancer [47]. It appears that our network analysis reiterates this concept; however, further implications of this finding in NET biology need to be investigated.

We further explored the CREB mechanism *in silico* by identifying possible CREB binding targets and cAMP response elements among the significantly altered genes using the Salk CREB Target Gene database [22]. These CRE targets encoded genes responsible for nucleosome assembly (*NAP1L3*, *TSPYL4*), regulation of transcription (*TERF2IP*), organism development (*BEX1*, *INA*), secretion (*CHGB*, *SCG2*, *SCG3*, *SYN1*) and adhesion (*TRO*). Because we were specifically interested in genes involved in cAMP-mediated secretory processes, we examined this subset further.

The accumulation of cAMP in response to activation of GPCRs induces a wide range of cellular processes including transcription, metabolism, cell cycle progression and apoptosis through the PKA pathway [48]. In this study, transcript of *PRKAR1A*, the type 1alpha regulatory subunit (RIalpha) of PKA, was up-regulated in KRJ-I and stimulated by forskolin and BIM-53061. This is consistent with the function of these compounds as inducers of the cAMP pathway. The selective D_2_R and 5-HT receptor agonist, BIM-53061 appears to be at least as potent a cAMP recruiter as either forskolin or isoproterenol particularly for secretory gene transcription. This suggests the involvement of the dopamine/5-HT-mediated pathway in the recruitment of intracellular cAMP/PKA activation. In addition, *PRKAR1A* transcript levels stimulated by BIM-53061 were consistent with the accumulation of intracellular cAMP suggesting a direct involvement of neural GPCR receptor activation with *ADCY2* and *PRKAR1A* recruitment and subsequent PKA-induced CREB phosphorylation.

We have demonstrated that elevation in cAMP is associated with normal and neoplastic EC cell secretion [18], [49]. In the present study, we identified elevated levels of *ADCY2* and *PRKAR1A* transcripts in a database of SI NETs compared to normal SI mucosa, suggesting that cAMP signaling may indeed be activated in tumor cells [32], [50]. ADCY2 is a class B member of the Adenylate Cyclase (ADCY) which is calcium insensitive but is stimulated by G_βγ_ subunits of heterotrimeric G-proteins and is therefore directly coupled with GPCRs [35]. In the KRJ-I cell line, transcript levels of *ADCY2* were sensitive to forskolin and BIM-53061, which suggests that cAMP-induced transcription may occur through activation of this cyclase regulator in the KRJ-I cell line.

Cellular gene expression is regulated following CREB protein phosphorylation at serine residue 133 [21], [51]. This occurs as a consequence of cAMP accumulation which liberates the C subunits of PKA that passively diffuse into the nucleus and induce CREB phosphorylation. CREB is an important transcription factor activated by multiple signal transduction pathways in response to external stimuli, including synaptic activity, hormones, growth factors, cytokines, and stress [15]. It affects cellular functions and enhances growth, increases angiogenesis, and decreases apoptosis. We identified *in silico* and demonstrated *in vitro* cAMP-mediated regulation of seven putative CREB targets – *BEX1* (modulates nerve growth factor [NGF] signaling through nuclear factor-kappaB [NFkB] to regulate cell cycle, apoptosis, and differentiation in neural tissues [38]), *BICD1* (structural constituent of cytoskeleton [39]), *CHGB* (neuroendocrine cell-specific gene, which may play a role in early tumor development but is also a NET secretory product [16], [52], *CPE* (pulmonary NET marker [29]), *GABRB3* (characteristic GABA receptor of normal and neoplastic human EC cells and controlled through CREB [20], [36], [40]), *SCG2* (secreted neuroendocrine marker observed in prostatic small-cell neuroendocrine carcinoma [30]) and *SCG3* (secretory product commonly expressed in pituitary adenomas [53]). *In vitro* investigations of these CREs indicate that cAMP activation through either adenylate cyclase activation (forskolin) or through neural GPCR activation (isoproterenol and dopamine/5-HT) resulted in gene expression. CRE transcription correlated with cAMP levels: BIM-53061, which was associated with the highest cAMP (2.8-fold) accumulation, was also associated with the majority of genes transcribed (100%). Both forskolin and isoproterenol stimulated cAMP levels 1.8-fold and were associated with 42–57% of target genes being transcribed. The suggestion that gene transcription was cAMP concentration-dependent was reinforced by the observation that forskolin (10^−6^ M), which did not significantly elevate cAMP, was not associated with CRE target transcription. Similar investigations in the rat pituitary cell line GH4 have shown that forskolin-induced cAMP accumulation results in an increase of prolactin and growth hormone gene transcription [54], suggesting that a single intracellular mediator can simultaneously regulate the transcription of different sets of responsive genes by stimulating independent biochemical events.

The study provides an illustration of how genome-wide network inference can be used to infer CRE-mediated transcription in neoplastic cell lines and has implications for defining the mechanisms of NET proliferation and secretion. Similar studies examining the progression of cancer [55], [56], heart disease [57], neuropsychiatric disorders [58], [59], asthma pathogenesis [60], and the analysis of factors associated with infertility [61] have provided information in regard to these disease processes. We propose that the application of this methodology to the investigation of NETs or other diseases associated with abnormal EC cell secretion, like Crohn's disease [6] or IBS [62], will provide significant mechanistic information on the cell regulatory phenomena. Our current data demonstrates that neoplastic EC cells over-express regulators in the cAMP signaling pathway and that activation of neural GPCRs results in proliferative and secretory gene transcription thus providing novel information regarding the neural activation of tumor behavior. This investigative strategy, that emphasizes co-expression network inference, provides a useful tool to define and delineate the mechanisms involved in the mechanistic cellular basis of the clinical manifestations of NET disease. It is likely that the application of this technique will facilitate the identification of specific regulatory elements that can be targeted for therapeutic gain.

## Materials and Methods

### Statistical analyses of Affymetrix GeneChip data

Raw expression data for each of the 13 microarray experiments (Affymetrix U133A; normal mucosa: *n* = 4; primary SI NETs: *n* = 9) was normalized using the MAS5.0 algorithm available through the Bioconductor suit [63] for the R statistical language [64]. Affymetrix probe identifiers (IDs) were mapped to their corresponding Ensembl (September 26, 2010) gene IDs [65]. In cases where multiple probesets mapped to a single gene, only median signal intensity was retained. Data is deposited in the ArrayExpress database (accession number: E-GEOD-6272).

### Gene Network Inference

Pairwise similarity in gene expression vectors was expressed by the PCC. Gene pairs that correlated above a predefined PCC threshold value were represented in the form of an undirected weighted network, where nodes (vertices) correspond to genes and links (edges) correspond to co-expression between genes. The Maslov-Sneppen randomized network model was generated by rewiring edges in the original network while preserving the degrees of the respective nodes [66]. The number of rewiring steps taken for each model was 4× (number of edges). This method ensures that the topological structure of the network is retained during randomization. The Erdős–Rényi random model was generated by retaining the nodes of the original network and building edges using a uniform probability [67].

### Network Topology Concepts

Topological properties examined were node degree, network diameter, betweenness centrality, connected components, clustering coefficient, and modularity [68]. Node degree is defined as the total number of edges that connect to a given node. Network diameter is defined as the average shortest path between any pair of nodes in the network. Betweenness centrality is the measure of node importance within a graph, where nodes that occur on many shortest paths between nodes have higher betweenness. Connected components are maximal connected subgraphs of an undirected graph in which any two vertices are connected to each other by edges. Clustering coefficient is the degree to which nodes tend to cluster together. Modularity quantifies the capacity of a network to divide into clusters or communities. Higher modularity indicates a favorable partition.

### Network clustering and functional enrichment

Clusters of genes in a co-expression network were identified using the Louvain method, a fast algorithm for community detection in graphs [69]. The Louvain method is a greedy algorithm for iterative grouping of nodes into communities based on optimization of modularity [45]. A distinct advantage of this method is its parameter-free architecture that allows unbiased exploration of network structure. Because clusters of co-expressed genes are known to be functionally related [27], functional enrichment for GO-BP terms was performed. For a cluster with *n* genes and an *a priori* defined functional category with *K* genes, the hypergeometric test was used to evaluate the significance of the overlap *k* between the cluster and a functional category [70]. All genes in a network were used as reference.

### Pathway Analysis

Over-represented pathway analysis was performed using the DAVID functional annotation tool [46] and prediction of CREB target phosphorylation was assessed using CREB target gene database (http://natural.salk.edu/CREB/) [22] with a confidence level of the binding value (BV)≤0.001 and a binding ratio (BR)≥1.5.

### Validation and *in vitro* experiments

#### Culture Conditions

KRJ-I cells, derived from a “typical” SI NET [18], [71], were cultured as floating aggregates at 37°C with 5% CO_2_. Cells were kept in Ham's F12 medium (Gibco™) containing 10% fetal bovine serum (FBS) (Sigma-Aldrich), penicillin 100 U/ml, and streptomycin 100 µg/ml [18], [25].

#### Real-Time PCR

To validate the presence of genes involved in cAMP-mediated transcription pathway, two approaches were undertaken. In the first approach, transcripts for selected CREs, *ADCY2*, and *PRKAR1A* were measured in an independent data set of neoplastic EC cell line KRJ-I (*n* = 10) and normal EC cell preparations (*n* = 8) using real-time PCR. In the second approach, the effect of forskolin (10^−5^ M and 10^−6^ M), isoproterenol (10^−5^ M), and BIM-53061 (10^−6^ M) was measured on target transcription in KRJ-I cells. KRJ-I cells (5×10^4^ cells/well, in triplicate) were stimulated for 2 hours and RNA was extracted from 1×10^6^ cells in log phase growth (TRIZOL®, Invitrogen, USA). Real time RT-PCR analysis was performed using Assays-on-Demand™ products and the ABI 7900 Sequence Detection System according to the manufacturer's suggestions. Cycling was performed under standard conditions (TaqMan® Universal PCR Master Mix Protocol) and data normalized using GeNorm [42] and expression of the novel house-keeping genes, *ALG9*, *TFCP2* and *ZNF410* [41].

#### cAMP and PKA Activation

To test whether KRJ-I cells were physiologically responsive to neural GPCR agonists, intracellular cAMP accumulation in response to the three stimulants after 20 mins was assayed using a cAMP ELISA assay (R&D Research, Minneapolis, MN). PKA activity was quantitated in the same samples (Enzo Life Sciences, Butler Pike, PA). Cells (5×10^4^ cells/well, in triplicate) were stimulated with forskolin (10^−5^ M, 10^−6^ M), isoproterenol (10^−5^ M), and BIM-53061 (10^−6^ M) after which cells were lysed with 0.1 N HCL and freezing. All samples and controls were acetylated prior to performing the cAMP ELISA (R&D cAMP ELISA handbook). PKA activity was determined according to the manufacturer's recommendations. Lysed samples were incubated with 20 µl PKA reaction mixture at 30°C for 30 min. The reaction was terminated and activity quantitated versus levels of a highly specific substrate using an ELISA protocol. Absorbance readings for either cAMP or PKA were measured at 450 nm on a microplate reader (Bio-Rad 3500).

#### pCREB quantitation - western Blotting

KRJ-I cells (4×10^5^ cells/ml) were seeded in 6 well plates (Falcon, BD, Franklin Lakes, NJ) and treated with each of the agents for 15 and 60 mins. After cells were harvested, whole-cell lysates were prepared by adding 200 µl of ice-cold cell lysis buffer (10× RIPA lysis buffer (Millipore, Billerica, MA), complete protease inhibitor [Roche, Indianapolis, IN], phosphatase inhibitor set 1&2 (Calbiochem, Gibbstown, NJ), 100 mM PMSF (Roche), 200 mM Na_3_VO_4_ (Acros Organics), 12.5 mg/ml SDS (American Bioanalytical, Natick, MA). Tubes were centrifuged at 12,000 g for 20 min and protein amount in the supernatant was quantified using the BCA protein assay kit (Thermo Fisher Scientific, Rockford, IL). For western blot, total protein lysates (20 µg) were denaturated in SDS sample buffer, separated on an SDS-PAGE gel (4, 10%) and transferred to a PVDF membrane (Bio-Rad, Hercules, CA, pore size 0.45 mm). After blocking (5% BSA for 60 min at room temperature) the membrane was incubated with the phospho-CREB (Ser133) primary antibody (Cell Signaling Technology, Danvers, MA) in 5% BSA/PBS/Tween 20 overnight at 4°C. The membranes were incubated with the horseradish peroxidase-conjugated secondary antibodies (Cell Signaling Technology) for 60 min at room temperature and immunodetection was performed using the Western Lightning™ Plus-ECL (PerkinElmer, MA). Blots were exposed on X-OMAT-AR films. The optical density of the appropriately sized bands was measured using ImageJ software (NIH, USA). The ratio between phospho-protein expression was reported relative to that of β-actin (Sigma-Aldrich, MO).

## Supporting Information

Figure S1 **SI NET network properties as functions of Pearson correlation coefficient (PCC).** For each PCC cutoff, the number of nodes, number of edges, number of connected components, and network density were measured. It was noted that at PCC≥0.94, the SI NET network was most modular while retaining a reasonable number of genes and links.
(TIF)

Figure S2 **cAMP/CREB signaling cascade.** Differentially expressed elements identified using gene network inference are highlighted in red and annotated.
(TIF)

Table S1 **SI NET interactome.**
(DOC)
