# Supplementary figures and images for: Gene Network Inference and Biochemical Assessment Delineates GPCR Pathways and CREB Targets in Small Intestinal Neuroendocrine Neoplasia
Source: PLoS One. 2011 Aug 11;6(8):e22457. doi: 10.1371/journal.pone.0022457 (PMC3154895; doi:10.1371/journal.pone.0022457)

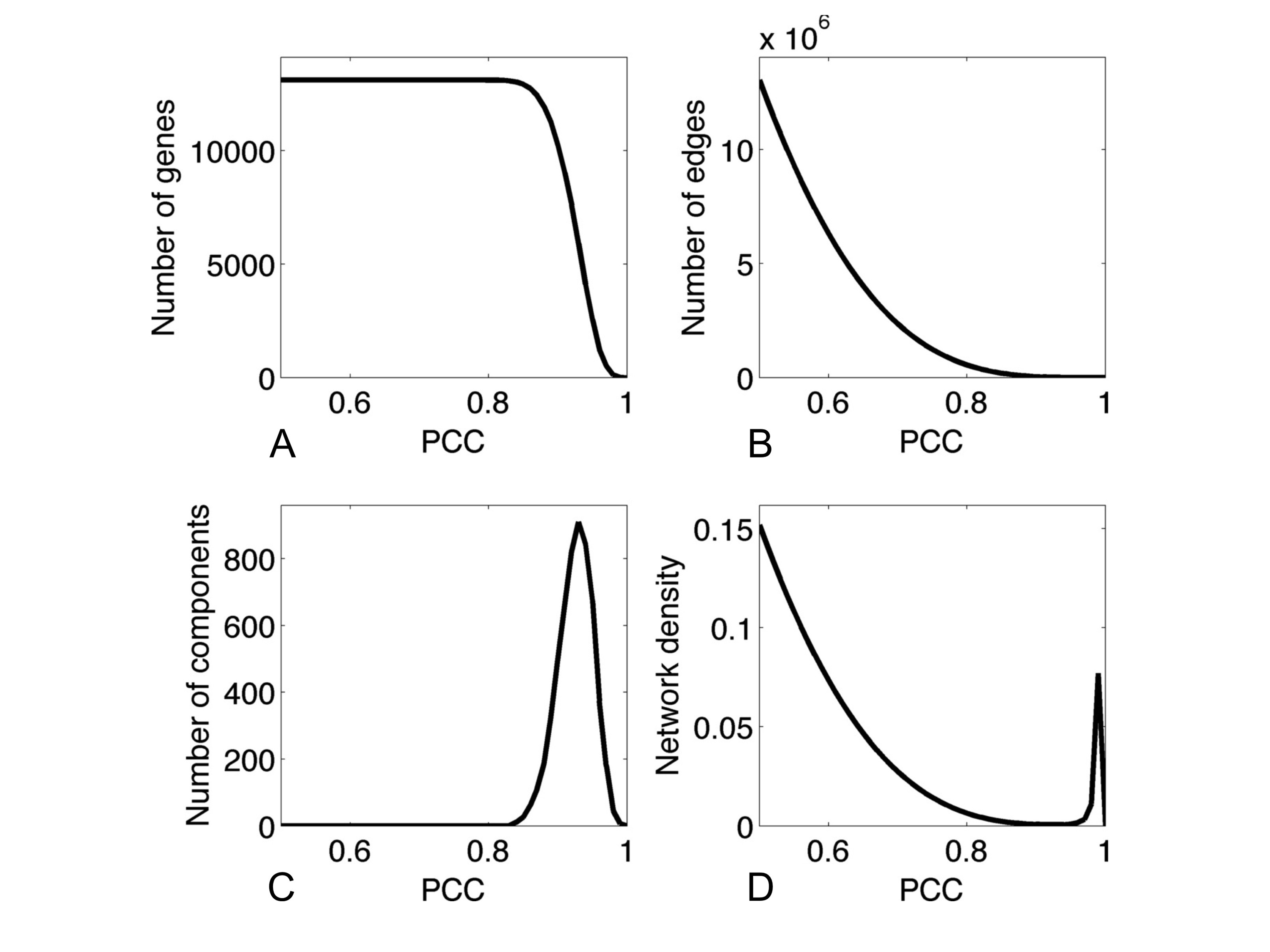

Supplement: Figure S1 — SI NET network properties as functions of Pearson correlation coefficient (PCC). For each PCC cutoff, the number of nodes, number of edges, number of connected components, and network density were measured. It was noted that at PCC≥0.94, the SI NET network was most modular while retaining a reasonable number of genes and links. (TIF) [file pone.0022457.s001.tif]

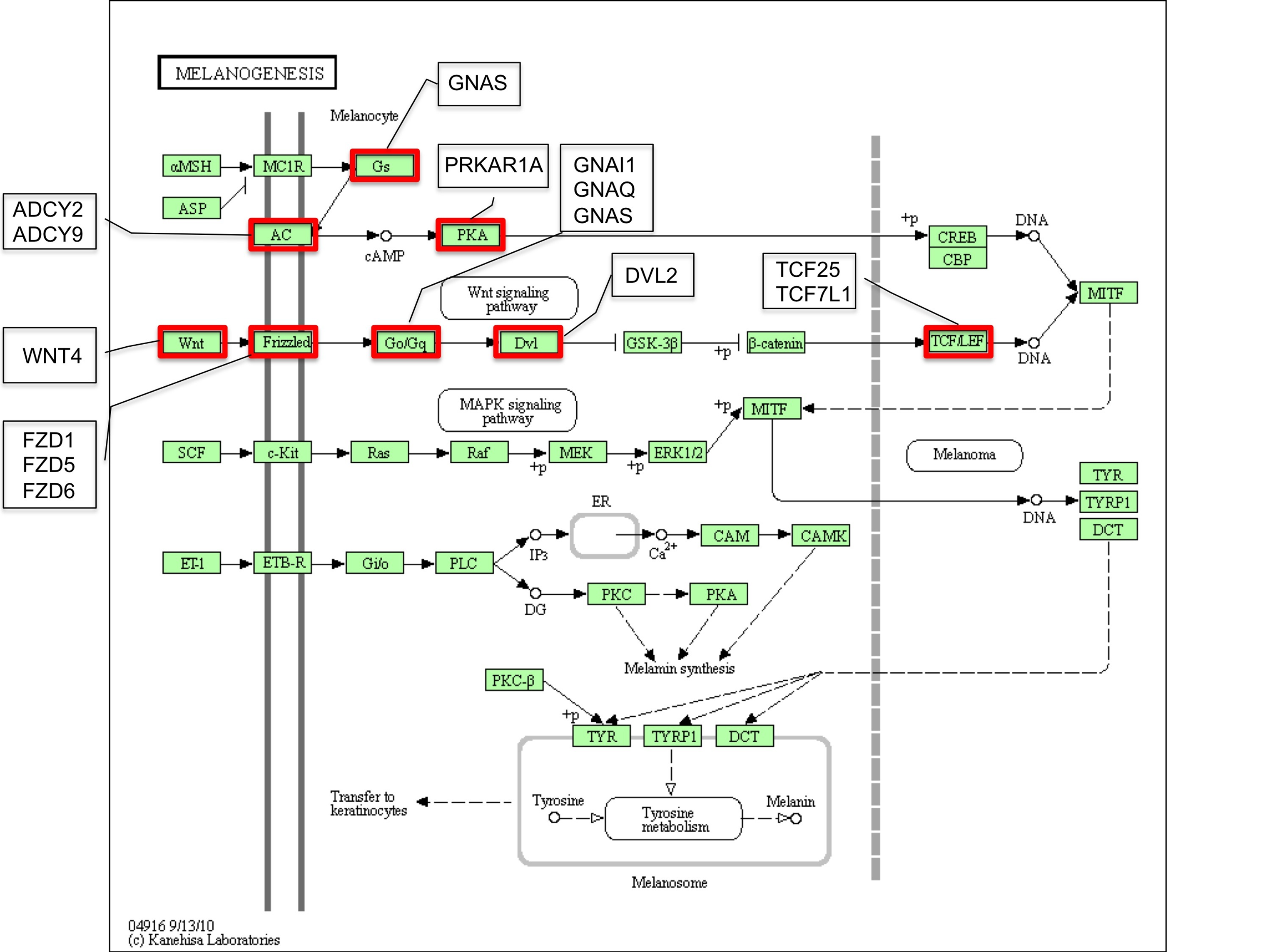

Supplement: Figure S2 — cAMP/CREB signaling cascade. Differentially expressed elements identified using gene network inference are highlighted in red and annotated. (TIF) [file pone.0022457.s002.tif]
