# Supplementary material for: Gene Network Inference and Biochemical Assessment Delineates GPCR Pathways and CREB Targets in Small Intestinal Neuroendocrine Neoplasia
Source: PLoS One. 2011 Aug 11;6(8):e22457. doi: 10.1371/journal.pone.0022457 (PMC3154895; doi:10.1371/journal.pone.0022457)
Supplement: Table S1 — SI NET interactome. (DOC) [file pone.0022457.s003.doc]

**TableS1. SI NET Interactome (excel spreadsheet).**

| Node A | Node B | PCC |
| --- | --- | --- |
| ENSG00000005187 | ENSG00000019169 | 0.96 |
| ENSG00000015413 | ENSG00000021488 | 0.97 |
| ENSG00000007933 | ENSG00000021852 | 0.96 |
| ENSG00000003436 | ENSG00000044090 | -0.95 |
| ENSG00000039537 | ENSG00000047457 | 0.97 |
| ENSG00000006611 | ENSG00000048740 | 0.94 |
| ENSG00000049249 | ENSG00000053702 | 0.96 |
| ENSG00000015676 | ENSG00000056558 | 0.96 |
| ENSG00000029534 | ENSG00000058404 | 0.95 |
| ENSG00000010810 | ENSG00000060069 | 0.96 |
| ENSG00000048740 | ENSG00000064763 | 0.95 |
| ENSG00000059573 | ENSG00000065154 | 0.95 |
| ENSG00000008735 | ENSG00000066032 | 0.95 |
| ENSG00000047457 | ENSG00000066813 | 0.96 |
| ENSG00000066322 | ENSG00000066813 | -0.95 |
| ENSG00000012817 | ENSG00000067048 | 0.95 |
| ENSG00000005187 | ENSG00000067057 | -0.95 |
| ENSG00000039537 | ENSG00000067057 | -0.97 |
| ENSG00000047457 | ENSG00000067057 | -0.95 |
| ENSG00000061676 | ENSG00000067248 | 0.96 |
| ENSG00000054356 | ENSG00000067836 | 0.95 |
| ENSG00000058404 | ENSG00000068971 | 0.95 |
| ENSG00000008735 | ENSG00000069122 | 0.95 |
| ENSG00000047249 | ENSG00000069399 | -0.96 |
| ENSG00000048740 | ENSG00000070019 | 0.97 |
| ENSG00000055118 | ENSG00000070444 | 0.96 |
| ENSG00000060709 | ENSG00000070444 | 0.95 |
| ENSG00000022267 | ENSG00000070501 | -0.96 |
| ENSG00000006611 | ENSG00000071073 | 0.96 |
| ENSG00000068438 | ENSG00000071282 | 0.95 |
| ENSG00000054356 | ENSG00000072041 | 0.96 |
| ENSG00000066032 | ENSG00000072041 | 0.98 |
| ENSG00000028116 | ENSG00000072864 | 0.95 |
| ENSG00000006116 | ENSG00000073350 | -0.95 |
| ENSG00000073711 | ENSG00000074696 | -0.94 |
| ENSG00000070019 | ENSG00000075340 | -0.95 |
| ENSG00000047249 | ENSG00000076258 | -0.95 |
| ENSG00000067369 | ENSG00000076650 | 0.95 |
| ENSG00000008838 | ENSG00000077254 | 0.95 |
| ENSG00000077254 | ENSG00000079102 | 0.95 |
| ENSG00000039537 | ENSG00000079557 | 0.97 |
| ENSG00000067167 | ENSG00000079689 | -0.96 |
| ENSG00000004975 | ENSG00000080823 | 0.97 |
| ENSG00000023330 | ENSG00000080854 | -0.95 |
| ENSG00000019169 | ENSG00000080910 | 0.96 |
| ENSG00000039537 | ENSG00000080910 | 0.96 |
| ENSG00000070081 | ENSG00000083535 | 0.96 |
| ENSG00000073111 | ENSG00000083720 | 0.94 |
| ENSG00000081760 | ENSG00000083720 | 0.95 |
| ENSG00000072080 | ENSG00000083807 | 0.97 |
| ENSG00000023839 | ENSG00000083896 | -0.97 |
| ENSG00000019169 | ENSG00000084110 | 0.96 |
| ENSG00000039537 | ENSG00000084110 | 0.94 |
| ENSG00000066336 | ENSG00000085365 | -0.95 |
| ENSG00000060709 | ENSG00000085511 | 0.97 |
| ENSG00000062598 | ENSG00000086475 | 0.95 |
| ENSG00000080910 | ENSG00000087237 | 0.94 |
| ENSG00000084754 | ENSG00000088682 | 0.96 |
| ENSG00000077279 | ENSG00000089053 | 0.97 |
| ENSG00000054356 | ENSG00000089199 | 0.96 |
| ENSG00000080986 | ENSG00000089685 | 0.95 |
| ENSG00000088448 | ENSG00000090013 | -0.97 |
| ENSG00000082014 | ENSG00000090863 | 0.95 |
| ENSG00000016490 | ENSG00000090920 | 0.95 |
| ENSG00000066455 | ENSG00000091527 | -0.95 |
| ENSG00000077279 | ENSG00000091972 | 0.95 |
| ENSG00000084207 | ENSG00000095002 | 0.97 |
| ENSG00000072163 | ENSG00000095637 | 0.95 |
| ENSG00000085514 | ENSG00000096006 | -0.97 |
| ENSG00000067369 | ENSG00000096093 | 0.95 |
| ENSG00000041515 | ENSG00000096696 | -0.96 |
| ENSG00000079257 | ENSG00000096872 | 0.97 |
| ENSG00000089902 | ENSG00000096872 | 0.95 |
| ENSG00000070087 | ENSG00000099326 | 0.95 |
| ENSG00000089091 | ENSG00000099326 | 0.95 |
| ENSG00000064102 | ENSG00000099341 | -0.96 |
| ENSG00000022267 | ENSG00000099834 | 0.95 |
| ENSG00000070501 | ENSG00000099834 | -0.97 |
| ENSG00000019169 | ENSG00000099937 | 0.96 |
| ENSG00000080910 | ENSG00000099937 | 0.95 |
| ENSG00000084110 | ENSG00000099937 | 0.96 |
| ENSG00000087237 | ENSG00000099937 | 0.94 |
| ENSG00000067836 | ENSG00000100014 | 0.96 |
| ENSG00000089053 | ENSG00000100014 | 0.95 |
| ENSG00000022277 | ENSG00000100029 | 0.95 |
| ENSG00000077809 | ENSG00000100029 | 0.95 |
| ENSG00000072121 | ENSG00000100292 | -0.95 |
| ENSG00000054356 | ENSG00000100505 | 0.95 |
| ENSG00000066279 | ENSG00000100526 | 0.96 |
| ENSG00000088325 | ENSG00000100526 | 0.98 |
| ENSG00000100292 | ENSG00000100554 | -0.95 |
| ENSG00000006327 | ENSG00000100650 | -0.95 |
| ENSG00000019169 | ENSG00000100652 | 0.95 |
| ENSG00000047457 | ENSG00000100652 | 0.95 |
| ENSG00000080910 | ENSG00000100652 | 0.96 |
| ENSG00000096654 | ENSG00000100652 | -0.95 |
| ENSG00000067057 | ENSG00000100665 | -0.95 |
| ENSG00000006625 | ENSG00000100731 | -0.97 |
| ENSG00000066813 | ENSG00000100908 | -0.96 |
| ENSG00000090054 | ENSG00000100908 | 0.97 |
| ENSG00000010404 | ENSG00000101084 | -0.95 |
| ENSG00000072121 | ENSG00000101191 | 0.96 |
| ENSG00000100296 | ENSG00000101216 | 0.95 |
| ENSG00000035115 | ENSG00000101333 | 0.97 |
| ENSG00000051825 | ENSG00000101361 | 0.95 |
| ENSG00000068615 | ENSG00000101384 | 0.94 |
| ENSG00000092969 | ENSG00000101448 | 0.94 |
| ENSG00000088035 | ENSG00000101546 | 0.96 |
| ENSG00000084207 | ENSG00000101596 | 0.95 |
| ENSG00000076864 | ENSG00000101940 | 0.95 |
| ENSG00000084463 | ENSG00000101981 | -0.97 |
| ENSG00000075073 | ENSG00000102038 | -0.95 |
| ENSG00000011304 | ENSG00000102054 | 0.95 |
| ENSG00000054793 | ENSG00000102078 | 0.95 |
| ENSG00000019505 | ENSG00000102109 | 0.94 |
| ENSG00000060709 | ENSG00000102109 | 0.97 |
| ENSG00000100889 | ENSG00000102226 | -0.95 |
| ENSG00000004487 | ENSG00000102241 | 0.95 |
| ENSG00000008838 | ENSG00000102678 | 0.96 |
| ENSG00000079102 | ENSG00000102678 | 0.97 |
| ENSG00000079739 | ENSG00000102710 | -0.95 |
| ENSG00000048052 | ENSG00000102743 | -0.95 |
| ENSG00000067606 | ENSG00000102886 | 0.95 |
| ENSG00000019169 | ENSG00000102967 | 0.96 |
| ENSG00000039537 | ENSG00000102967 | 0.94 |
| ENSG00000047457 | ENSG00000102967 | 0.96 |
| ENSG00000066813 | ENSG00000102967 | 0.96 |
| ENSG00000080910 | ENSG00000102967 | 0.96 |
| ENSG00000013275 | ENSG00000102974 | 0.96 |
| ENSG00000005020 | ENSG00000103037 | -0.95 |
| ENSG00000072041 | ENSG00000103154 | 0.97 |
| ENSG00000100504 | ENSG00000103175 | -0.95 |
| ENSG00000078043 | ENSG00000103184 | 0.96 |
| ENSG00000006059 | ENSG00000103202 | 0.95 |
| ENSG00000102554 | ENSG00000103202 | -0.95 |
| ENSG00000065802 | ENSG00000103365 | 0.98 |
| ENSG00000072786 | ENSG00000103381 | 0.96 |
| ENSG00000021300 | ENSG00000103460 | 0.95 |
| ENSG00000102241 | ENSG00000103460 | 0.95 |
| ENSG00000072832 | ENSG00000103723 | 0.96 |
| ENSG00000031823 | ENSG00000103876 | -0.96 |
| ENSG00000005421 | ENSG00000103942 | 0.95 |
| ENSG00000006128 | ENSG00000104093 | 0.95 |
| ENSG00000100441 | ENSG00000104093 | 0.96 |
| ENSG00000072832 | ENSG00000104112 | 0.97 |
| ENSG00000103723 | ENSG00000104112 | 0.95 |
| ENSG00000065833 | ENSG00000104327 | 0.95 |
| ENSG00000103335 | ENSG00000104375 | -0.98 |
| ENSG00000099326 | ENSG00000104381 | 0.95 |
| ENSG00000069020 | ENSG00000104450 | 0.95 |
| ENSG00000008056 | ENSG00000104723 | 0.95 |
| ENSG00000019169 | ENSG00000104760 | 0.95 |
| ENSG00000039537 | ENSG00000104760 | 0.95 |
| ENSG00000072080 | ENSG00000104760 | 0.97 |
| ENSG00000080910 | ENSG00000104760 | 0.98 |
| ENSG00000083807 | ENSG00000104760 | 0.95 |
| ENSG00000015475 | ENSG00000104765 | -0.96 |
| ENSG00000078549 | ENSG00000104897 | 0.95 |
| ENSG00000072080 | ENSG00000104915 | -0.95 |
| ENSG00000005187 | ENSG00000104938 | 0.97 |
| ENSG00000019169 | ENSG00000104938 | 0.98 |
| ENSG00000072080 | ENSG00000104938 | 0.95 |
| ENSG00000080910 | ENSG00000104938 | 0.97 |
| ENSG00000087237 | ENSG00000104938 | 0.97 |
| ENSG00000099937 | ENSG00000104938 | 0.95 |
| ENSG00000100652 | ENSG00000104938 | 0.96 |
| ENSG00000102967 | ENSG00000104938 | 0.95 |
| ENSG00000104760 | ENSG00000104938 | 0.96 |
| ENSG00000095261 | ENSG00000104960 | 0.95 |
| ENSG00000095637 | ENSG00000104960 | -0.95 |
| ENSG00000068028 | ENSG00000105202 | 0.95 |
| ENSG00000000971 | ENSG00000105216 | -0.97 |
| ENSG00000087157 | ENSG00000105216 | 0.94 |
| ENSG00000100288 | ENSG00000105270 | 0.97 |
| ENSG00000102241 | ENSG00000105287 | 0.95 |
| ENSG00000052344 | ENSG00000105289 | 0.94 |
| ENSG00000072274 | ENSG00000105289 | 0.96 |
| ENSG00000100170 | ENSG00000105289 | 0.95 |
| ENSG00000072832 | ENSG00000105290 | 0.96 |
| ENSG00000085063 | ENSG00000105290 | 0.95 |
| ENSG00000103723 | ENSG00000105290 | 0.97 |
| ENSG00000104112 | ENSG00000105290 | 0.96 |
| ENSG00000088448 | ENSG00000105379 | -0.97 |
| ENSG00000090013 | ENSG00000105379 | 0.98 |
| ENSG00000019169 | ENSG00000105697 | 0.95 |
| ENSG00000039537 | ENSG00000105697 | 0.95 |
| ENSG00000080910 | ENSG00000105697 | 0.96 |
| ENSG00000102967 | ENSG00000105697 | 0.96 |
| ENSG00000104760 | ENSG00000105697 | 0.95 |
| ENSG00000105402 | ENSG00000105928 | -0.95 |
| ENSG00000105552 | ENSG00000105971 | -0.98 |
| ENSG00000096006 | ENSG00000105991 | 0.95 |
| ENSG00000102109 | ENSG00000106089 | 0.96 |
| ENSG00000092203 | ENSG00000106278 | 0.95 |
| ENSG00000083807 | ENSG00000106327 | 0.95 |
| ENSG00000104760 | ENSG00000106327 | 0.95 |
| ENSG00000104413 | ENSG00000106538 | -0.94 |
| ENSG00000078269 | ENSG00000106546 | -0.96 |
| ENSG00000011275 | ENSG00000106565 | -0.95 |
| ENSG00000049246 | ENSG00000106772 | 0.96 |
| ENSG00000047457 | ENSG00000106804 | 0.96 |
| ENSG00000066322 | ENSG00000106804 | -0.96 |
| ENSG00000066813 | ENSG00000106804 | 0.96 |
| ENSG00000067057 | ENSG00000106804 | -0.95 |
| ENSG00000090054 | ENSG00000106804 | -0.95 |
| ENSG00000105835 | ENSG00000106804 | 0.95 |
| ENSG00000007933 | ENSG00000106927 | 0.95 |
| ENSG00000080910 | ENSG00000106927 | 0.95 |
| ENSG00000099769 | ENSG00000106927 | 0.96 |
| ENSG00000055118 | ENSG00000106976 | 0.95 |
| ENSG00000060709 | ENSG00000106976 | 0.95 |
| ENSG00000102109 | ENSG00000106976 | 0.95 |
| ENSG00000006747 | ENSG00000107020 | 0.94 |
| ENSG00000102241 | ENSG00000107104 | -0.94 |
| ENSG00000100028 | ENSG00000107223 | 0.95 |
| ENSG00000055118 | ENSG00000107281 | 0.95 |
| ENSG00000106976 | ENSG00000107281 | 0.96 |
| ENSG00000006712 | ENSG00000107611 | -0.94 |
| ENSG00000072163 | ENSG00000107611 | 0.95 |
| ENSG00000092096 | ENSG00000107758 | 0.96 |
| ENSG00000083807 | ENSG00000107779 | -0.95 |
| ENSG00000100227 | ENSG00000107937 | 0.95 |
| ENSG00000067955 | ENSG00000107957 | 0.98 |
| ENSG00000101955 | ENSG00000107968 | 0.96 |
| ENSG00000085063 | ENSG00000108021 | 0.94 |
| ENSG00000089053 | ENSG00000108021 | 0.96 |
| ENSG00000095585 | ENSG00000108242 | 0.95 |
| ENSG00000105576 | ENSG00000108242 | -0.95 |
| ENSG00000040199 | ENSG00000108272 | 0.95 |
| ENSG00000106404 | ENSG00000108272 | 0.97 |
| ENSG00000072832 | ENSG00000108309 | 0.96 |
| ENSG00000103723 | ENSG00000108309 | 0.94 |
| ENSG00000104112 | ENSG00000108309 | 0.98 |
| ENSG00000105290 | ENSG00000108309 | 0.97 |
| ENSG00000003756 | ENSG00000108349 | 0.95 |
| ENSG00000080031 | ENSG00000108352 | 0.96 |
| ENSG00000108272 | ENSG00000108352 | 0.96 |
| ENSG00000081760 | ENSG00000108666 | 0.96 |
| ENSG00000083720 | ENSG00000108666 | 0.95 |
| ENSG00000102967 | ENSG00000108666 | -0.95 |
| ENSG00000088986 | ENSG00000108733 | 0.95 |
| ENSG00000100292 | ENSG00000108799 | -0.95 |
| ENSG00000085063 | ENSG00000108840 | 0.95 |
| ENSG00000093144 | ENSG00000108840 | -0.95 |
| ENSG00000036565 | ENSG00000108852 | 0.95 |
| ENSG00000054356 | ENSG00000108852 | 0.96 |
| ENSG00000066032 | ENSG00000108852 | 0.96 |
| ENSG00000072041 | ENSG00000108852 | 0.95 |
| ENSG00000083799 | ENSG00000108852 | 0.94 |
| ENSG00000089199 | ENSG00000108852 | 0.96 |
| ENSG00000073111 | ENSG00000108924 | -0.95 |
| ENSG00000063177 | ENSG00000109062 | -0.95 |
| ENSG00000068971 | ENSG00000109066 | 0.96 |
| ENSG00000064393 | ENSG00000109103 | 0.95 |
| ENSG00000087494 | ENSG00000109158 | 0.95 |
| ENSG00000100605 | ENSG00000109158 | 0.96 |
| ENSG00000101126 | ENSG00000109189 | 0.96 |
| ENSG00000060709 | ENSG00000109339 | 0.95 |
| ENSG00000102109 | ENSG00000109339 | 0.95 |
| ENSG00000041982 | ENSG00000109466 | -0.96 |
| ENSG00000104812 | ENSG00000109466 | -0.94 |
| ENSG00000105290 | ENSG00000109472 | 0.95 |
| ENSG00000108309 | ENSG00000109472 | 0.95 |
| ENSG00000109339 | ENSG00000109472 | 0.95 |
| ENSG00000091972 | ENSG00000109501 | 0.96 |
| ENSG00000100714 | ENSG00000109572 | -0.95 |
| ENSG00000004487 | ENSG00000109667 | -0.95 |
| ENSG00000075239 | ENSG00000109736 | -0.95 |
| ENSG00000060140 | ENSG00000109756 | -0.95 |
| ENSG00000019169 | ENSG00000109758 | 0.97 |
| ENSG00000039537 | ENSG00000109758 | 0.95 |
| ENSG00000080910 | ENSG00000109758 | 0.98 |
| ENSG00000087237 | ENSG00000109758 | 0.97 |
| ENSG00000099937 | ENSG00000109758 | 0.96 |
| ENSG00000100652 | ENSG00000109758 | 0.95 |
| ENSG00000102967 | ENSG00000109758 | 0.96 |
| ENSG00000104760 | ENSG00000109758 | 0.97 |
| ENSG00000104938 | ENSG00000109758 | 0.98 |
| ENSG00000105697 | ENSG00000109758 | 0.95 |
| ENSG00000105497 | ENSG00000109929 | -0.96 |
| ENSG00000083799 | ENSG00000109956 | 0.95 |
| ENSG00000101825 | ENSG00000110042 | -0.96 |
| ENSG00000100156 | ENSG00000110092 | 0.94 |
| ENSG00000100053 | ENSG00000110148 | 0.95 |
| ENSG00000079557 | ENSG00000110169 | 0.95 |
| ENSG00000080910 | ENSG00000110169 | 0.98 |
| ENSG00000102967 | ENSG00000110169 | 0.96 |
| ENSG00000104760 | ENSG00000110169 | 0.95 |
| ENSG00000104938 | ENSG00000110169 | 0.95 |
| ENSG00000105697 | ENSG00000110169 | 0.96 |
| ENSG00000109758 | ENSG00000110169 | 0.98 |
| ENSG00000072518 | ENSG00000110367 | -0.97 |
| ENSG00000099956 | ENSG00000110436 | -0.97 |
| ENSG00000105568 | ENSG00000110492 | 0.96 |
| ENSG00000049246 | ENSG00000110497 | 0.95 |
| ENSG00000004399 | ENSG00000110514 | 0.95 |
| ENSG00000073050 | ENSG00000110514 | 0.96 |
| ENSG00000105398 | ENSG00000110514 | -0.95 |
| ENSG00000103876 | ENSG00000110583 | -0.96 |
| ENSG00000108576 | ENSG00000110628 | 0.96 |
| ENSG00000007062 | ENSG00000110651 | -0.95 |
| ENSG00000084764 | ENSG00000110880 | -0.96 |
| ENSG00000077279 | ENSG00000110888 | 0.97 |
| ENSG00000089053 | ENSG00000110888 | 0.96 |
| ENSG00000101199 | ENSG00000110925 | 0.96 |
| ENSG00000081059 | ENSG00000110934 | 0.96 |
| ENSG00000104499 | ENSG00000110987 | -0.95 |
| ENSG00000039537 | ENSG00000111181 | 0.95 |
| ENSG00000075240 | ENSG00000111181 | 0.95 |
| ENSG00000079557 | ENSG00000111181 | 0.96 |
| ENSG00000080910 | ENSG00000111181 | 0.96 |
| ENSG00000084110 | ENSG00000111181 | 0.94 |
| ENSG00000099937 | ENSG00000111181 | 0.95 |
| ENSG00000104760 | ENSG00000111181 | 0.94 |
| ENSG00000109758 | ENSG00000111181 | 0.96 |
| ENSG00000110169 | ENSG00000111181 | 0.95 |
| ENSG00000076003 | ENSG00000111206 | 0.96 |
| ENSG00000106462 | ENSG00000111206 | 0.96 |
| ENSG00000073111 | ENSG00000111247 | 0.96 |
| ENSG00000079739 | ENSG00000111275 | 0.95 |
| ENSG00000102710 | ENSG00000111275 | -0.97 |
| ENSG00000052344 | ENSG00000111319 | 0.95 |
| ENSG00000100554 | ENSG00000111540 | 0.95 |
| ENSG00000109339 | ENSG00000111674 | 0.95 |
| ENSG00000109472 | ENSG00000111674 | 0.95 |
| ENSG00000073584 | ENSG00000111679 | 0.95 |
| ENSG00000101333 | ENSG00000111679 | 0.96 |
| ENSG00000065618 | ENSG00000111701 | 0.95 |
| ENSG00000100028 | ENSG00000111801 | 0.95 |
| ENSG00000068745 | ENSG00000111885 | -0.95 |
| ENSG00000103254 | ENSG00000111907 | -0.94 |
| ENSG00000083290 | ENSG00000112096 | -0.95 |
| ENSG00000047621 | ENSG00000112200 | 0.95 |
| ENSG00000077044 | ENSG00000112210 | 0.95 |
| ENSG00000104973 | ENSG00000112249 | 0.94 |
| ENSG00000105186 | ENSG00000112282 | 0.95 |
| ENSG00000104388 | ENSG00000112306 | -0.95 |
| ENSG00000104938 | ENSG00000112337 | 0.94 |
| ENSG00000100201 | ENSG00000112378 | -0.95 |
| ENSG00000072832 | ENSG00000112511 | 0.96 |
| ENSG00000104112 | ENSG00000112511 | 0.96 |
| ENSG00000103426 | ENSG00000112531 | -0.95 |
| ENSG00000060709 | ENSG00000112561 | 0.95 |
| ENSG00000063180 | ENSG00000112576 | 0.97 |
| ENSG00000004779 | ENSG00000112697 | -0.95 |
| ENSG00000087586 | ENSG00000112742 | 0.95 |
| ENSG00000101003 | ENSG00000112742 | 0.98 |
| ENSG00000103335 | ENSG00000112992 | -0.95 |
| ENSG00000110777 | ENSG00000113083 | -0.95 |
| ENSG00000007384 | ENSG00000113108 | -0.96 |
| ENSG00000050393 | ENSG00000113108 | -0.96 |
| ENSG00000095713 | ENSG00000113119 | -0.96 |
| ENSG00000005187 | ENSG00000113141 | -0.95 |
| ENSG00000019169 | ENSG00000113141 | -0.95 |
| ENSG00000039537 | ENSG00000113141 | -0.95 |
| ENSG00000047457 | ENSG00000113141 | -0.97 |
| ENSG00000066813 | ENSG00000113141 | -0.95 |
| ENSG00000067057 | ENSG00000113141 | 0.97 |
| ENSG00000080910 | ENSG00000113141 | -0.94 |
| ENSG00000102967 | ENSG00000113141 | -0.96 |
| ENSG00000106804 | ENSG00000113141 | -0.97 |
| ENSG00000081177 | ENSG00000113163 | 0.96 |
| ENSG00000089248 | ENSG00000113231 | -0.96 |
| ENSG00000077498 | ENSG00000113249 | 0.94 |
| ENSG00000112559 | ENSG00000113249 | 0.95 |
| ENSG00000047249 | ENSG00000113360 | 0.96 |
| ENSG00000076258 | ENSG00000113360 | -0.94 |
| ENSG00000111206 | ENSG00000113368 | 0.95 |
| ENSG00000102935 | ENSG00000113407 | -0.95 |
| ENSG00000101448 | ENSG00000113552 | -0.95 |
| ENSG00000010256 | ENSG00000113555 | -0.96 |
| ENSG00000030110 | ENSG00000113555 | -0.95 |
| ENSG00000021852 | ENSG00000113600 | 0.98 |
| ENSG00000103351 | ENSG00000113615 | -0.95 |
| ENSG00000096433 | ENSG00000113648 | 0.97 |
| ENSG00000091138 | ENSG00000113722 | 0.95 |
| ENSG00000039537 | ENSG00000113889 | 0.96 |
| ENSG00000047457 | ENSG00000113889 | 0.96 |
| ENSG00000079557 | ENSG00000113889 | 0.95 |
| ENSG00000080910 | ENSG00000113889 | 0.96 |
| ENSG00000100652 | ENSG00000113889 | 0.95 |
| ENSG00000101981 | ENSG00000113889 | 0.95 |
| ENSG00000007933 | ENSG00000113905 | 0.95 |
| ENSG00000019169 | ENSG00000113905 | 0.96 |
| ENSG00000039537 | ENSG00000113905 | 0.94 |
| ENSG00000080910 | ENSG00000113905 | 0.97 |
| ENSG00000084110 | ENSG00000113905 | 0.97 |
| ENSG00000099937 | ENSG00000113905 | 0.95 |
| ENSG00000104760 | ENSG00000113905 | 0.96 |
| ENSG00000104938 | ENSG00000113905 | 0.95 |
| ENSG00000106927 | ENSG00000113905 | 0.97 |
| ENSG00000109758 | ENSG00000113905 | 0.95 |
| ENSG00000110169 | ENSG00000113905 | 0.95 |
| ENSG00000111181 | ENSG00000113905 | 0.95 |
| ENSG00000089356 | ENSG00000114098 | 0.95 |
| ENSG00000106993 | ENSG00000114098 | -0.94 |
| ENSG00000077279 | ENSG00000114279 | 0.96 |
| ENSG00000091972 | ENSG00000114279 | 0.95 |
| ENSG00000110888 | ENSG00000114279 | 0.96 |
| ENSG00000113361 | ENSG00000114279 | 0.96 |
| ENSG00000090889 | ENSG00000114346 | 0.95 |
| ENSG00000012817 | ENSG00000114374 | 0.95 |
| ENSG00000067048 | ENSG00000114374 | 0.95 |
| ENSG00000100889 | ENSG00000114405 | -0.96 |
| ENSG00000091138 | ENSG00000114455 | 0.95 |
| ENSG00000108272 | ENSG00000114455 | 0.96 |
| ENSG00000017427 | ENSG00000114529 | -0.94 |
| ENSG00000104419 | ENSG00000114529 | 0.97 |
| ENSG00000106993 | ENSG00000114529 | -0.96 |
| ENSG00000063177 | ENSG00000114544 | 0.96 |
| ENSG00000004399 | ENSG00000114631 | 0.95 |
| ENSG00000072832 | ENSG00000114631 | 0.96 |
| ENSG00000091129 | ENSG00000114631 | 0.97 |
| ENSG00000102003 | ENSG00000114631 | 0.95 |
| ENSG00000104112 | ENSG00000114631 | 0.97 |
| ENSG00000108309 | ENSG00000114631 | 0.95 |
| ENSG00000112511 | ENSG00000114631 | 0.96 |
| ENSG00000104872 | ENSG00000114735 | 0.94 |
| ENSG00000010322 | ENSG00000114770 | 0.96 |
| ENSG00000100083 | ENSG00000114786 | 0.96 |
| ENSG00000079102 | ENSG00000114933 | -0.96 |
| ENSG00000096093 | ENSG00000114956 | 0.95 |
| ENSG00000103024 | ENSG00000114956 | 0.96 |
| ENSG00000101191 | ENSG00000114988 | 0.96 |
| ENSG00000019505 | ENSG00000115020 | 0.96 |
| ENSG00000058404 | ENSG00000115020 | 0.95 |
| ENSG00000060709 | ENSG00000115020 | 0.95 |
| ENSG00000069020 | ENSG00000115020 | 0.96 |
| ENSG00000102109 | ENSG00000115020 | 0.96 |
| ENSG00000104450 | ENSG00000115020 | 0.95 |
| ENSG00000105146 | ENSG00000115194 | 0.94 |
| ENSG00000106571 | ENSG00000115211 | 0.96 |
| ENSG00000100116 | ENSG00000115226 | 0.96 |
| ENSG00000106541 | ENSG00000115226 | -0.95 |
| ENSG00000106993 | ENSG00000115226 | 0.98 |
| ENSG00000114098 | ENSG00000115226 | -0.95 |
| ENSG00000114529 | ENSG00000115226 | -0.95 |
| ENSG00000106404 | ENSG00000115263 | 0.97 |
| ENSG00000108272 | ENSG00000115263 | 0.95 |
| ENSG00000113722 | ENSG00000115263 | 0.96 |
| ENSG00000095321 | ENSG00000115310 | 0.94 |
| ENSG00000115263 | ENSG00000115386 | 0.96 |
| ENSG00000107186 | ENSG00000115414 | 0.95 |
| ENSG00000102882 | ENSG00000115457 | -0.95 |
| ENSG00000101213 | ENSG00000115474 | 0.95 |
| ENSG00000075914 | ENSG00000115484 | 0.96 |
| ENSG00000087237 | ENSG00000115561 | -0.96 |
| ENSG00000095794 | ENSG00000115602 | 0.96 |
| ENSG00000096093 | ENSG00000115806 | 0.95 |
| ENSG00000072121 | ENSG00000115840 | 0.95 |
| ENSG00000072080 | ENSG00000115942 | -0.95 |
| ENSG00000089234 | ENSG00000115942 | 0.94 |
| ENSG00000111602 | ENSG00000115966 | 0.96 |
| ENSG00000112245 | ENSG00000116016 | 0.95 |
| ENSG00000049541 | ENSG00000116062 | 0.95 |
| ENSG00000092758 | ENSG00000116106 | 0.96 |
| ENSG00000023839 | ENSG00000116128 | -0.95 |
| ENSG00000055118 | ENSG00000116128 | 0.95 |
| ENSG00000099326 | ENSG00000116128 | 0.97 |
| ENSG00000107281 | ENSG00000116128 | 0.95 |
| ENSG00000107295 | ENSG00000116128 | 0.95 |
| ENSG00000100156 | ENSG00000116157 | 0.95 |
| ENSG00000073331 | ENSG00000116191 | 0.95 |
| ENSG00000014257 | ENSG00000116212 | 0.94 |
| ENSG00000056972 | ENSG00000116212 | 0.95 |
| ENSG00000033327 | ENSG00000116266 | 0.94 |
| ENSG00000091972 | ENSG00000116337 | 0.96 |
| ENSG00000105697 | ENSG00000116353 | -0.95 |
| ENSG00000113889 | ENSG00000116353 | -0.96 |
| ENSG00000114107 | ENSG00000116406 | 0.95 |
| ENSG00000111049 | ENSG00000116580 | 0.96 |
| ENSG00000079112 | ENSG00000116667 | 0.96 |
| ENSG00000108349 | ENSG00000116675 | 0.95 |
| ENSG00000039537 | ENSG00000116678 | 0.95 |
| ENSG00000047457 | ENSG00000116678 | 0.97 |
| ENSG00000067057 | ENSG00000116678 | -0.95 |
| ENSG00000067369 | ENSG00000116678 | -0.95 |
| ENSG00000102967 | ENSG00000116678 | 0.95 |
| ENSG00000105697 | ENSG00000116678 | 0.95 |
| ENSG00000113141 | ENSG00000116678 | -0.95 |
| ENSG00000116016 | ENSG00000116678 | 0.97 |
| ENSG00000113302 | ENSG00000116752 | 0.96 |
| ENSG00000084674 | ENSG00000116771 | 0.95 |
| ENSG00000021826 | ENSG00000116833 | 0.95 |
| ENSG00000080910 | ENSG00000116882 | 0.96 |
| ENSG00000100652 | ENSG00000116882 | 0.95 |
| ENSG00000104760 | ENSG00000116882 | 0.94 |
| ENSG00000104938 | ENSG00000116882 | 0.94 |
| ENSG00000113600 | ENSG00000116882 | 0.95 |
| ENSG00000085511 | ENSG00000117020 | 0.94 |
| ENSG00000054356 | ENSG00000117069 | 0.95 |
| ENSG00000108852 | ENSG00000117069 | 0.94 |
| ENSG00000103152 | ENSG00000117115 | -0.97 |
| ENSG00000103381 | ENSG00000117133 | -0.95 |
| ENSG00000112182 | ENSG00000117151 | 0.95 |
| ENSG00000096093 | ENSG00000117153 | 0.95 |
| ENSG00000100263 | ENSG00000117153 | 0.95 |
| ENSG00000112996 | ENSG00000117155 | 0.95 |
| ENSG00000106344 | ENSG00000117385 | 0.95 |
| ENSG00000090013 | ENSG00000117411 | -0.95 |
| ENSG00000002726 | ENSG00000117472 | 0.95 |
| ENSG00000111537 | ENSG00000117560 | 0.95 |
| ENSG00000100632 | ENSG00000117569 | 0.97 |
| ENSG00000039537 | ENSG00000117594 | 0.96 |
| ENSG00000055957 | ENSG00000117594 | 0.95 |
| ENSG00000079557 | ENSG00000117594 | 0.96 |
| ENSG00000080910 | ENSG00000117594 | 0.98 |
| ENSG00000104760 | ENSG00000117594 | 0.97 |
| ENSG00000105697 | ENSG00000117594 | 0.94 |
| ENSG00000109758 | ENSG00000117594 | 0.97 |
| ENSG00000110169 | ENSG00000117594 | 0.97 |
| ENSG00000111181 | ENSG00000117594 | 0.97 |
| ENSG00000039537 | ENSG00000117601 | 0.96 |
| ENSG00000080910 | ENSG00000117601 | 0.97 |
| ENSG00000087237 | ENSG00000117601 | 0.95 |
| ENSG00000102967 | ENSG00000117601 | 0.95 |
| ENSG00000104760 | ENSG00000117601 | 0.96 |
| ENSG00000104938 | ENSG00000117601 | 0.95 |
| ENSG00000106927 | ENSG00000117601 | 0.95 |
| ENSG00000109758 | ENSG00000117601 | 0.97 |
| ENSG00000110169 | ENSG00000117601 | 0.96 |
| ENSG00000111181 | ENSG00000117601 | 0.96 |
| ENSG00000113905 | ENSG00000117601 | 0.95 |
| ENSG00000117594 | ENSG00000117601 | 0.96 |
| ENSG00000108576 | ENSG00000117620 | 0.95 |
| ENSG00000108187 | ENSG00000117632 | -0.95 |
| ENSG00000115163 | ENSG00000117650 | 0.95 |
| ENSG00000052344 | ENSG00000117676 | 0.96 |
| ENSG00000102934 | ENSG00000117676 | 0.96 |
| ENSG00000100526 | ENSG00000117724 | 0.95 |
| ENSG00000023839 | ENSG00000117791 | 0.94 |
| ENSG00000105254 | ENSG00000117791 | -0.95 |
| ENSG00000070081 | ENSG00000118137 | -0.95 |
| ENSG00000083535 | ENSG00000118137 | -0.95 |
| ENSG00000087237 | ENSG00000118520 | 0.95 |
| ENSG00000099769 | ENSG00000118520 | 0.95 |
| ENSG00000113905 | ENSG00000118520 | 0.94 |
| ENSG00000117601 | ENSG00000118520 | 0.96 |
| ENSG00000115234 | ENSG00000118557 | -0.96 |
| ENSG00000092531 | ENSG00000118729 | 0.95 |
| ENSG00000107554 | ENSG00000118777 | 0.95 |
| ENSG00000117620 | ENSG00000118777 | 0.96 |
| ENSG00000055732 | ENSG00000118898 | 0.95 |
| ENSG00000066032 | ENSG00000118898 | 0.94 |
| ENSG00000073969 | ENSG00000118898 | 0.95 |
| ENSG00000100652 | ENSG00000119185 | -0.94 |
| ENSG00000080618 | ENSG00000119401 | -0.95 |
| ENSG00000106927 | ENSG00000119401 | -0.95 |
| ENSG00000087237 | ENSG00000119514 | -0.95 |
| ENSG00000115561 | ENSG00000119514 | 0.95 |
| ENSG00000117009 | ENSG00000119514 | -0.94 |
| ENSG00000073350 | ENSG00000119537 | -0.97 |
| ENSG00000109832 | ENSG00000119630 | 0.95 |
| ENSG00000081760 | ENSG00000119640 | 0.96 |
| ENSG00000113532 | ENSG00000119655 | 0.97 |
| ENSG00000006327 | ENSG00000119682 | -0.95 |
| ENSG00000101417 | ENSG00000119684 | 0.96 |
| ENSG00000119682 | ENSG00000119685 | -0.94 |
| ENSG00000073111 | ENSG00000119711 | -0.95 |
| ENSG00000075239 | ENSG00000119711 | 0.95 |
| ENSG00000083720 | ENSG00000119711 | -0.95 |
| ENSG00000111247 | ENSG00000119711 | -0.95 |
| ENSG00000055732 | ENSG00000119782 | 0.95 |
| ENSG00000118898 | ENSG00000119782 | 0.98 |
| ENSG00000100116 | ENSG00000119888 | -0.95 |
| ENSG00000106541 | ENSG00000119888 | 0.97 |
| ENSG00000106993 | ENSG00000119888 | -0.95 |
| ENSG00000114529 | ENSG00000119888 | 0.94 |
| ENSG00000115226 | ENSG00000119888 | -0.98 |
| ENSG00000102977 | ENSG00000119965 | 0.96 |
| ENSG00000116761 | ENSG00000119965 | -0.95 |
| ENSG00000115461 | ENSG00000119969 | 0.94 |
| ENSG00000064601 | ENSG00000120029 | 0.96 |
| ENSG00000092068 | ENSG00000120029 | 0.95 |
| ENSG00000070087 | ENSG00000120053 | -0.95 |
| ENSG00000096093 | ENSG00000120053 | -0.96 |
| ENSG00000036565 | ENSG00000120251 | 0.95 |
| ENSG00000058404 | ENSG00000120251 | 0.97 |
| ENSG00000069020 | ENSG00000120251 | 0.95 |
| ENSG00000089199 | ENSG00000120251 | 0.96 |
| ENSG00000108852 | ENSG00000120251 | 0.96 |
| ENSG00000115020 | ENSG00000120251 | 0.97 |
| ENSG00000104723 | ENSG00000120645 | 0.95 |
| ENSG00000119630 | ENSG00000120645 | 0.96 |
| ENSG00000101955 | ENSG00000120693 | -0.95 |
| ENSG00000112039 | ENSG00000120705 | -0.98 |
| ENSG00000099812 | ENSG00000120756 | 0.95 |
| ENSG00000103089 | ENSG00000120756 | 0.96 |
| ENSG00000110583 | ENSG00000120885 | -0.96 |
| ENSG00000005810 | ENSG00000120899 | 0.95 |
| ENSG00000100979 | ENSG00000120925 | 0.94 |
| ENSG00000028310 | ENSG00000120948 | 0.95 |
| ENSG00000095002 | ENSG00000121031 | 0.95 |
| ENSG00000006634 | ENSG00000121621 | 0.94 |
| ENSG00000109016 | ENSG00000121634 | -0.96 |
| ENSG00000054356 | ENSG00000121671 | 0.95 |
| ENSG00000058404 | ENSG00000121671 | 0.94 |
| ENSG00000089199 | ENSG00000121671 | 0.95 |
| ENSG00000102109 | ENSG00000121671 | 0.96 |
| ENSG00000106089 | ENSG00000121671 | 0.98 |
| ENSG00000115020 | ENSG00000121671 | 0.95 |
| ENSG00000120251 | ENSG00000121671 | 0.96 |
| ENSG00000076650 | ENSG00000121691 | -0.95 |
| ENSG00000100802 | ENSG00000121691 | -0.96 |
| ENSG00000116539 | ENSG00000121764 | 0.97 |
| ENSG00000100307 | ENSG00000121905 | 0.95 |
| ENSG00000116745 | ENSG00000122025 | 0.94 |
| ENSG00000115474 | ENSG00000122121 | 0.96 |
| ENSG00000019169 | ENSG00000122194 | 0.97 |
| ENSG00000039537 | ENSG00000122194 | 0.95 |
| ENSG00000072080 | ENSG00000122194 | 0.95 |
| ENSG00000080910 | ENSG00000122194 | 0.96 |
| ENSG00000084110 | ENSG00000122194 | 0.96 |
| ENSG00000099937 | ENSG00000122194 | 0.95 |
| ENSG00000100652 | ENSG00000122194 | 0.96 |
| ENSG00000104760 | ENSG00000122194 | 0.97 |
| ENSG00000104938 | ENSG00000122194 | 0.97 |
| ENSG00000109758 | ENSG00000122194 | 0.97 |
| ENSG00000110169 | ENSG00000122194 | 0.95 |
| ENSG00000113905 | ENSG00000122194 | 0.96 |
| ENSG00000119965 | ENSG00000122194 | -0.94 |
| ENSG00000104517 | ENSG00000122224 | -0.94 |
| ENSG00000100983 | ENSG00000122254 | -0.94 |
| ENSG00000110429 | ENSG00000122299 | 0.96 |
| ENSG00000004948 | ENSG00000122678 | 0.95 |
| ENSG00000064225 | ENSG00000122705 | -0.96 |
| ENSG00000113722 | ENSG00000122711 | 0.94 |
| ENSG00000069399 | ENSG00000122729 | 0.94 |
| ENSG00000076258 | ENSG00000122729 | 0.95 |
| ENSG00000120910 | ENSG00000122729 | -0.96 |
| ENSG00000072080 | ENSG00000122787 | 0.97 |
| ENSG00000080910 | ENSG00000122787 | 0.97 |
| ENSG00000100652 | ENSG00000122787 | 0.96 |
| ENSG00000104760 | ENSG00000122787 | 0.97 |
| ENSG00000104915 | ENSG00000122787 | -0.96 |
| ENSG00000104938 | ENSG00000122787 | 0.96 |
| ENSG00000109758 | ENSG00000122787 | 0.95 |
| ENSG00000112337 | ENSG00000122787 | 0.95 |
| ENSG00000113905 | ENSG00000122787 | 0.95 |
| ENSG00000116882 | ENSG00000122787 | 0.97 |
| ENSG00000122194 | ENSG00000122787 | 0.96 |
| ENSG00000075785 | ENSG00000122873 | 0.95 |
| ENSG00000077942 | ENSG00000123096 | 0.95 |
| ENSG00000103540 | ENSG00000123143 | 0.95 |
| ENSG00000094914 | ENSG00000123415 | 0.95 |
| ENSG00000117748 | ENSG00000123444 | 0.95 |
| ENSG00000105835 | ENSG00000123453 | 0.96 |
| ENSG00000055957 | ENSG00000123473 | -0.95 |
| ENSG00000079557 | ENSG00000123561 | 0.95 |
| ENSG00000109758 | ENSG00000123561 | 0.97 |
| ENSG00000110169 | ENSG00000123561 | 0.95 |
| ENSG00000111181 | ENSG00000123561 | 0.95 |
| ENSG00000117594 | ENSG00000123561 | 0.95 |
| ENSG00000117601 | ENSG00000123561 | 0.96 |
| ENSG00000120949 | ENSG00000123561 | 0.95 |
| ENSG00000108395 | ENSG00000123562 | 0.95 |
| ENSG00000108848 | ENSG00000123562 | 0.95 |
| ENSG00000114631 | ENSG00000123562 | 0.95 |
| ENSG00000103067 | ENSG00000123610 | -0.95 |
| ENSG00000015479 | ENSG00000123636 | 0.96 |
| ENSG00000104517 | ENSG00000123636 | 0.97 |
| ENSG00000122224 | ENSG00000123636 | -0.95 |
| ENSG00000103490 | ENSG00000123643 | 0.95 |
| ENSG00000090659 | ENSG00000123737 | -0.94 |
| ENSG00000067057 | ENSG00000123838 | -0.94 |
| ENSG00000080910 | ENSG00000123838 | 0.95 |
| ENSG00000084110 | ENSG00000123838 | 0.95 |
| ENSG00000099937 | ENSG00000123838 | 0.96 |
| ENSG00000104760 | ENSG00000123838 | 0.95 |
| ENSG00000109758 | ENSG00000123838 | 0.95 |
| ENSG00000111181 | ENSG00000123838 | 0.97 |
| ENSG00000117594 | ENSG00000123838 | 0.96 |
| ENSG00000122194 | ENSG00000123838 | 0.96 |
| ENSG00000079277 | ENSG00000124232 | 0.95 |
| ENSG00000042445 | ENSG00000124253 | 0.95 |
| ENSG00000104325 | ENSG00000124253 | 0.95 |
| ENSG00000079112 | ENSG00000124570 | 0.95 |
| ENSG00000096080 | ENSG00000124688 | 0.95 |
| ENSG00000004478 | ENSG00000124701 | -0.95 |
| ENSG00000101825 | ENSG00000124749 | 0.95 |
| ENSG00000110042 | ENSG00000124749 | -0.95 |
| ENSG00000115963 | ENSG00000124766 | -0.95 |
| ENSG00000112651 | ENSG00000124935 | 0.95 |
| ENSG00000110245 | ENSG00000125144 | 0.96 |
| ENSG00000110696 | ENSG00000125144 | -0.95 |
| ENSG00000125144 | ENSG00000125148 | 0.98 |
| ENSG00000029993 | ENSG00000125170 | -0.95 |
| ENSG00000064692 | ENSG00000125170 | -0.95 |
| ENSG00000115461 | ENSG00000125170 | -0.95 |
| ENSG00000007062 | ENSG00000125520 | -0.95 |
| ENSG00000067646 | ENSG00000125522 | 0.95 |
| ENSG00000058404 | ENSG00000125675 | 0.94 |
| ENSG00000121691 | ENSG00000125676 | -0.95 |
| ENSG00000117054 | ENSG00000125695 | -0.95 |
| ENSG00000087157 | ENSG00000125730 | -0.95 |
| ENSG00000105216 | ENSG00000125730 | -0.95 |
| ENSG00000067057 | ENSG00000125735 | -0.96 |
| ENSG00000100665 | ENSG00000125735 | 0.96 |
| ENSG00000116678 | ENSG00000125735 | 0.97 |
| ENSG00000105202 | ENSG00000125743 | 0.94 |
| ENSG00000103024 | ENSG00000125744 | 0.94 |
| ENSG00000105254 | ENSG00000125744 | 0.95 |
| ENSG00000117791 | ENSG00000125744 | -0.96 |
| ENSG00000109472 | ENSG00000125755 | 0.95 |
| ENSG00000114631 | ENSG00000125755 | 0.95 |
| ENSG00000100292 | ENSG00000125779 | -0.96 |
| ENSG00000117114 | ENSG00000125810 | 0.95 |
| ENSG00000123094 | ENSG00000125815 | 0.97 |
| ENSG00000123415 | ENSG00000125827 | 0.95 |
| ENSG00000054356 | ENSG00000125851 | 0.95 |
| ENSG00000100505 | ENSG00000125851 | 0.95 |
| ENSG00000125877 | ENSG00000125970 | 0.97 |
| ENSG00000036565 | ENSG00000126106 | 0.96 |
| ENSG00000074964 | ENSG00000126106 | 0.95 |
| ENSG00000080910 | ENSG00000126231 | 0.95 |
| ENSG00000070444 | ENSG00000126243 | 0.95 |
| ENSG00000118058 | ENSG00000126746 | 0.95 |
| ENSG00000019169 | ENSG00000126759 | 0.95 |
| ENSG00000039537 | ENSG00000126759 | 0.95 |
| ENSG00000047457 | ENSG00000126759 | 0.95 |
| ENSG00000079557 | ENSG00000126759 | 0.95 |
| ENSG00000080910 | ENSG00000126759 | 0.96 |
| ENSG00000100652 | ENSG00000126759 | 0.96 |
| ENSG00000102967 | ENSG00000126759 | 0.97 |
| ENSG00000104938 | ENSG00000126759 | 0.96 |
| ENSG00000109758 | ENSG00000126759 | 0.98 |
| ENSG00000110169 | ENSG00000126759 | 0.97 |
| ENSG00000111181 | ENSG00000126759 | 0.95 |
| ENSG00000113889 | ENSG00000126759 | 0.95 |
| ENSG00000117601 | ENSG00000126759 | 0.96 |
| ENSG00000122194 | ENSG00000126759 | 0.96 |
| ENSG00000123561 | ENSG00000126759 | 0.96 |
| ENSG00000066279 | ENSG00000126787 | 0.96 |
| ENSG00000088325 | ENSG00000126787 | 0.95 |
| ENSG00000100526 | ENSG00000126787 | 0.95 |
| ENSG00000117650 | ENSG00000126787 | 0.95 |
| ENSG00000002822 | ENSG00000126814 | 0.94 |
| ENSG00000073734 | ENSG00000126821 | 0.96 |
| ENSG00000108946 | ENSG00000126822 | 0.94 |
| ENSG00000076650 | ENSG00000126858 | 0.96 |
| ENSG00000104938 | ENSG00000126858 | -0.95 |
| ENSG00000116678 | ENSG00000126858 | -0.95 |
| ENSG00000126759 | ENSG00000126858 | -0.96 |
| ENSG00000074695 | ENSG00000126947 | -0.97 |
| ENSG00000064115 | ENSG00000126950 | -0.95 |
| ENSG00000101180 | ENSG00000126970 | -0.95 |
| ENSG00000120875 | ENSG00000127129 | -0.95 |
| ENSG00000008735 | ENSG00000127252 | 0.96 |
| ENSG00000054356 | ENSG00000127252 | 0.98 |
| ENSG00000058404 | ENSG00000127252 | 0.95 |
| ENSG00000066032 | ENSG00000127252 | 0.95 |
| ENSG00000072041 | ENSG00000127252 | 0.96 |
| ENSG00000089199 | ENSG00000127252 | 0.97 |
| ENSG00000103154 | ENSG00000127252 | 0.96 |
| ENSG00000108852 | ENSG00000127252 | 0.97 |
| ENSG00000120251 | ENSG00000127252 | 0.96 |
| ENSG00000121671 | ENSG00000127252 | 0.95 |
| ENSG00000025434 | ENSG00000127511 | -0.96 |
| ENSG00000040341 | ENSG00000127527 | 0.95 |
| ENSG00000121361 | ENSG00000127616 | -0.95 |
| ENSG00000064763 | ENSG00000127831 | 0.96 |
| ENSG00000079112 | ENSG00000127831 | 0.95 |
| ENSG00000116667 | ENSG00000127831 | 0.97 |
| ENSG00000124570 | ENSG00000127831 | 0.96 |
| ENSG00000056050 | ENSG00000127922 | 0.96 |
| ENSG00000070087 | ENSG00000127948 | -0.95 |
| ENSG00000087903 | ENSG00000128185 | 0.96 |
| ENSG00000085117 | ENSG00000128242 | 0.95 |
| ENSG00000087157 | ENSG00000128283 | -0.94 |
| ENSG00000092964 | ENSG00000128563 | 0.95 |
| ENSG00000113716 | ENSG00000128563 | 0.96 |
| ENSG00000111913 | ENSG00000128564 | 0.94 |
| ENSG00000100365 | ENSG00000128606 | -0.95 |
| ENSG00000004487 | ENSG00000128656 | 0.96 |
| ENSG00000116016 | ENSG00000128656 | -0.96 |
| ENSG00000123201 | ENSG00000128683 | 0.97 |
| ENSG00000066813 | ENSG00000128731 | -0.95 |
| ENSG00000067225 | ENSG00000128731 | 0.95 |
| ENSG00000102967 | ENSG00000128731 | -0.95 |
| ENSG00000103024 | ENSG00000128731 | 0.95 |
| ENSG00000054356 | ENSG00000128918 | 0.94 |
| ENSG00000077279 | ENSG00000128918 | 0.94 |
| ENSG00000089053 | ENSG00000128918 | 0.95 |
| ENSG00000091972 | ENSG00000128918 | 0.95 |
| ENSG00000116337 | ENSG00000128918 | 0.95 |
| ENSG00000117069 | ENSG00000128918 | 0.97 |
| ENSG00000107798 | ENSG00000129028 | -0.94 |
| ENSG00000082014 | ENSG00000129038 | 0.95 |
| ENSG00000110514 | ENSG00000129158 | 0.96 |
| ENSG00000110628 | ENSG00000129187 | -0.96 |
| ENSG00000109320 | ENSG00000129195 | -0.95 |
| ENSG00000005187 | ENSG00000129214 | 0.95 |
| ENSG00000019169 | ENSG00000129214 | 0.95 |
| ENSG00000109758 | ENSG00000129214 | 0.95 |
| ENSG00000122194 | ENSG00000129214 | 0.95 |
| ENSG00000081041 | ENSG00000129351 | -0.95 |
| ENSG00000104413 | ENSG00000129354 | 0.95 |
| ENSG00000104419 | ENSG00000129354 | 0.95 |
| ENSG00000106993 | ENSG00000129354 | -0.97 |
| ENSG00000114529 | ENSG00000129354 | 0.98 |
| ENSG00000115226 | ENSG00000129354 | -0.96 |
| ENSG00000126243 | ENSG00000129422 | -0.94 |
| ENSG00000006611 | ENSG00000129473 | 0.96 |
| ENSG00000100065 | ENSG00000129473 | 0.94 |
| ENSG00000105492 | ENSG00000129559 | -0.96 |
| ENSG00000110092 | ENSG00000129675 | 0.94 |
| ENSG00000120645 | ENSG00000129682 | 0.95 |
| ENSG00000071564 | ENSG00000129757 | 0.94 |
| ENSG00000012817 | ENSG00000129824 | 0.97 |
| ENSG00000067048 | ENSG00000129824 | 0.96 |
| ENSG00000114374 | ENSG00000129824 | 0.96 |
| ENSG00000047457 | ENSG00000129965 | 0.95 |
| ENSG00000099956 | ENSG00000129965 | -0.96 |
| ENSG00000106804 | ENSG00000129965 | 0.95 |
| ENSG00000110436 | ENSG00000129965 | 0.96 |
| ENSG00000113141 | ENSG00000129965 | -0.97 |
| ENSG00000019169 | ENSG00000129988 | 0.95 |
| ENSG00000039537 | ENSG00000129988 | 0.96 |
| ENSG00000067057 | ENSG00000129988 | -0.95 |
| ENSG00000080910 | ENSG00000129988 | 0.99 |
| ENSG00000084110 | ENSG00000129988 | 0.95 |
| ENSG00000087237 | ENSG00000129988 | 0.95 |
| ENSG00000099937 | ENSG00000129988 | 0.97 |
| ENSG00000104760 | ENSG00000129988 | 0.97 |
| ENSG00000104938 | ENSG00000129988 | 0.96 |
| ENSG00000106927 | ENSG00000129988 | 0.97 |
| ENSG00000109758 | ENSG00000129988 | 0.97 |
| ENSG00000110169 | ENSG00000129988 | 0.97 |
| ENSG00000111181 | ENSG00000129988 | 0.97 |
| ENSG00000113889 | ENSG00000129988 | 0.95 |
| ENSG00000113905 | ENSG00000129988 | 0.98 |
| ENSG00000117594 | ENSG00000129988 | 0.97 |
| ENSG00000117601 | ENSG00000129988 | 0.96 |
| ENSG00000122194 | ENSG00000129988 | 0.96 |
| ENSG00000122787 | ENSG00000129988 | 0.95 |
| ENSG00000123838 | ENSG00000129988 | 0.97 |
| ENSG00000126759 | ENSG00000129988 | 0.95 |
| ENSG00000115414 | ENSG00000130024 | -0.95 |
| ENSG00000108039 | ENSG00000130176 | 0.96 |
| ENSG00000113905 | ENSG00000130208 | 0.95 |
| ENSG00000100170 | ENSG00000130234 | 0.97 |
| ENSG00000108272 | ENSG00000130234 | 0.95 |
| ENSG00000111701 | ENSG00000130234 | 0.95 |
| ENSG00000114455 | ENSG00000130234 | 0.96 |
| ENSG00000115474 | ENSG00000130234 | 0.95 |
| ENSG00000122121 | ENSG00000130234 | 0.97 |
| ENSG00000104888 | ENSG00000130309 | 0.95 |
| ENSG00000114204 | ENSG00000130368 | 0.95 |
| ENSG00000103723 | ENSG00000130540 | 0.96 |
| ENSG00000006530 | ENSG00000130559 | 0.95 |
| ENSG00000087269 | ENSG00000130561 | 0.95 |
| ENSG00000108821 | ENSG00000130635 | 0.96 |
| ENSG00000036565 | ENSG00000130643 | 0.95 |
| ENSG00000083799 | ENSG00000130643 | 0.96 |
| ENSG00000121671 | ENSG00000130643 | 0.96 |
| ENSG00000019169 | ENSG00000130649 | 0.95 |
| ENSG00000066813 | ENSG00000130649 | 0.95 |
| ENSG00000072080 | ENSG00000130649 | 0.96 |
| ENSG00000080910 | ENSG00000130649 | 0.96 |
| ENSG00000083807 | ENSG00000130649 | 0.96 |
| ENSG00000104760 | ENSG00000130649 | 0.96 |
| ENSG00000104938 | ENSG00000130649 | 0.95 |
| ENSG00000106327 | ENSG00000130649 | 0.95 |
| ENSG00000109758 | ENSG00000130649 | 0.97 |
| ENSG00000117601 | ENSG00000130649 | 0.98 |
| ENSG00000123561 | ENSG00000130649 | 0.96 |
| ENSG00000081913 | ENSG00000130684 | -0.94 |
| ENSG00000117143 | ENSG00000130684 | -0.96 |
| ENSG00000079999 | ENSG00000130703 | 0.96 |
| ENSG00000111275 | ENSG00000130707 | 0.94 |
| ENSG00000124253 | ENSG00000130707 | 0.95 |
| ENSG00000125148 | ENSG00000130707 | 0.95 |
| ENSG00000023839 | ENSG00000130723 | -0.95 |
| ENSG00000071991 | ENSG00000130723 | -0.95 |
| ENSG00000054523 | ENSG00000130787 | 0.95 |
| ENSG00000119888 | ENSG00000130787 | 0.95 |
| ENSG00000130176 | ENSG00000130821 | 0.97 |
| ENSG00000106785 | ENSG00000130827 | -0.95 |
| ENSG00000103266 | ENSG00000130985 | 0.95 |
| ENSG00000115263 | ENSG00000131096 | 0.95 |
| ENSG00000035687 | ENSG00000131149 | 0.96 |
| ENSG00000035115 | ENSG00000131165 | 0.95 |
| ENSG00000111679 | ENSG00000131165 | 0.96 |
| ENSG00000091129 | ENSG00000131238 | 0.95 |
| ENSG00000102003 | ENSG00000131238 | 0.95 |
| ENSG00000104112 | ENSG00000131238 | 0.94 |
| ENSG00000108848 | ENSG00000131238 | 0.95 |
| ENSG00000089723 | ENSG00000131435 | 0.94 |
| ENSG00000101004 | ENSG00000131437 | 0.95 |
| ENSG00000084674 | ENSG00000131482 | 0.96 |
| ENSG00000110011 | ENSG00000131626 | 0.96 |
| ENSG00000091129 | ENSG00000131669 | -0.95 |
| ENSG00000020633 | ENSG00000131730 | 0.97 |
| ENSG00000105655 | ENSG00000131773 | 0.96 |
| ENSG00000107798 | ENSG00000131773 | -0.95 |
| ENSG00000070087 | ENSG00000131779 | 0.96 |
| ENSG00000085433 | ENSG00000131779 | 0.95 |
| ENSG00000089091 | ENSG00000131779 | 0.95 |
| ENSG00000070087 | ENSG00000131788 | 0.95 |
| ENSG00000103540 | ENSG00000131788 | 0.95 |
| ENSG00000109794 | ENSG00000131831 | -0.95 |
| ENSG00000116171 | ENSG00000131845 | -0.96 |
| ENSG00000131482 | ENSG00000131845 | -0.97 |
| ENSG00000103485 | ENSG00000132170 | 0.95 |
| ENSG00000100364 | ENSG00000132182 | 0.96 |
| ENSG00000019505 | ENSG00000132359 | 0.96 |
| ENSG00000060709 | ENSG00000132359 | 0.95 |
| ENSG00000102109 | ENSG00000132359 | 0.98 |
| ENSG00000115020 | ENSG00000132359 | 0.96 |
| ENSG00000118194 | ENSG00000132429 | 0.95 |
| ENSG00000073350 | ENSG00000132437 | 0.95 |
| ENSG00000125386 | ENSG00000132466 | 0.95 |
| ENSG00000101977 | ENSG00000132514 | -0.95 |
| ENSG00000103490 | ENSG00000132517 | 0.95 |
| ENSG00000123643 | ENSG00000132517 | 0.96 |
| ENSG00000005187 | ENSG00000132541 | 0.95 |
| ENSG00000075239 | ENSG00000132541 | 0.97 |
| ENSG00000087237 | ENSG00000132541 | 0.95 |
| ENSG00000102967 | ENSG00000132541 | 0.94 |
| ENSG00000104938 | ENSG00000132541 | 0.97 |
| ENSG00000108666 | ENSG00000132541 | -0.96 |
| ENSG00000109758 | ENSG00000132541 | 0.97 |
| ENSG00000110169 | ENSG00000132541 | 0.95 |
| ENSG00000122194 | ENSG00000132541 | 0.95 |
| ENSG00000126759 | ENSG00000132541 | 0.97 |
| ENSG00000126858 | ENSG00000132541 | -0.97 |
| ENSG00000129214 | ENSG00000132541 | 0.96 |
| ENSG00000106089 | ENSG00000132639 | 0.95 |
| ENSG00000119630 | ENSG00000132639 | 0.95 |
| ENSG00000120645 | ENSG00000132639 | 0.97 |
| ENSG00000106772 | ENSG00000132670 | 0.95 |
| ENSG00000122224 | ENSG00000132680 | -0.95 |
| ENSG00000039537 | ENSG00000132693 | 0.96 |
| ENSG00000075240 | ENSG00000132693 | 0.95 |
| ENSG00000079557 | ENSG00000132693 | 0.95 |
| ENSG00000080910 | ENSG00000132693 | 0.94 |
| ENSG00000106927 | ENSG00000132693 | 0.95 |
| ENSG00000111181 | ENSG00000132693 | 0.96 |
| ENSG00000113889 | ENSG00000132693 | 0.95 |
| ENSG00000113905 | ENSG00000132693 | 0.97 |
| ENSG00000119401 | ENSG00000132693 | -0.96 |
| ENSG00000125970 | ENSG00000132693 | -0.95 |
| ENSG00000129988 | ENSG00000132693 | 0.95 |
| ENSG00000104413 | ENSG00000132698 | 0.95 |
| ENSG00000104419 | ENSG00000132698 | 0.98 |
| ENSG00000106541 | ENSG00000132698 | 0.97 |
| ENSG00000106993 | ENSG00000132698 | -0.96 |
| ENSG00000114529 | ENSG00000132698 | 0.98 |
| ENSG00000115226 | ENSG00000132698 | -0.96 |
| ENSG00000119888 | ENSG00000132698 | 0.95 |
| ENSG00000129354 | ENSG00000132698 | 0.98 |
| ENSG00000101966 | ENSG00000132702 | 0.95 |
| ENSG00000039537 | ENSG00000132703 | 0.97 |
| ENSG00000072080 | ENSG00000132703 | 0.96 |
| ENSG00000079557 | ENSG00000132703 | 0.95 |
| ENSG00000080910 | ENSG00000132703 | 0.96 |
| ENSG00000104760 | ENSG00000132703 | 0.97 |
| ENSG00000111181 | ENSG00000132703 | 0.96 |
| ENSG00000113889 | ENSG00000132703 | 0.95 |
| ENSG00000113905 | ENSG00000132703 | 0.95 |
| ENSG00000117594 | ENSG00000132703 | 0.96 |
| ENSG00000117601 | ENSG00000132703 | 0.96 |
| ENSG00000120949 | ENSG00000132703 | 0.96 |
| ENSG00000122787 | ENSG00000132703 | 0.96 |
| ENSG00000129988 | ENSG00000132703 | 0.96 |
| ENSG00000130649 | ENSG00000132703 | 0.95 |
| ENSG00000132693 | ENSG00000132703 | 0.97 |
| ENSG00000100028 | ENSG00000132716 | -0.96 |
| ENSG00000102109 | ENSG00000132718 | 0.95 |
| ENSG00000103723 | ENSG00000132718 | 0.95 |
| ENSG00000106089 | ENSG00000132718 | 0.95 |
| ENSG00000109339 | ENSG00000132718 | 0.95 |
| ENSG00000109472 | ENSG00000132718 | 0.94 |
| ENSG00000132639 | ENSG00000132718 | 0.94 |
| ENSG00000123561 | ENSG00000132840 | 0.96 |
| ENSG00000068796 | ENSG00000132855 | -0.95 |
| ENSG00000128268 | ENSG00000132964 | 0.96 |
| ENSG00000112992 | ENSG00000133030 | -0.96 |
| ENSG00000060237 | ENSG00000133114 | 0.95 |
| ENSG00000118058 | ENSG00000133114 | 0.95 |
| ENSG00000101825 | ENSG00000133135 | -0.95 |
| ENSG00000123201 | ENSG00000133135 | -0.94 |
| ENSG00000128683 | ENSG00000133135 | -0.95 |
| ENSG00000008056 | ENSG00000133169 | 0.97 |
| ENSG00000074695 | ENSG00000133169 | -0.95 |
| ENSG00000099949 | ENSG00000133169 | 0.95 |
| ENSG00000104723 | ENSG00000133169 | 0.96 |
| ENSG00000120645 | ENSG00000133169 | 0.96 |
| ENSG00000132639 | ENSG00000133169 | 0.95 |
| ENSG00000112624 | ENSG00000133318 | 0.96 |
| ENSG00000010438 | ENSG00000133392 | 0.95 |
| ENSG00000015413 | ENSG00000133392 | 0.96 |
| ENSG00000073050 | ENSG00000133475 | -0.95 |
| ENSG00000114405 | ENSG00000133624 | 0.94 |
| ENSG00000004455 | ENSG00000133661 | -0.94 |
| ENSG00000011243 | ENSG00000133835 | -0.96 |
| ENSG00000070087 | ENSG00000133835 | -0.95 |
| ENSG00000105227 | ENSG00000134013 | 0.96 |
| ENSG00000088325 | ENSG00000134057 | 0.94 |
| ENSG00000106462 | ENSG00000134057 | 0.95 |
| ENSG00000128710 | ENSG00000134160 | 0.98 |
| ENSG00000115263 | ENSG00000134215 | 0.95 |
| ENSG00000084463 | ENSG00000134287 | 0.98 |
| ENSG00000101981 | ENSG00000134287 | -0.97 |
| ENSG00000112561 | ENSG00000134333 | -0.95 |
| ENSG00000039537 | ENSG00000134365 | 0.97 |
| ENSG00000072080 | ENSG00000134365 | 0.96 |
| ENSG00000080910 | ENSG00000134365 | 0.96 |
| ENSG00000102967 | ENSG00000134365 | 0.95 |
| ENSG00000104760 | ENSG00000134365 | 0.97 |
| ENSG00000105697 | ENSG00000134365 | 0.95 |
| ENSG00000106327 | ENSG00000134365 | 0.95 |
| ENSG00000106927 | ENSG00000134365 | 0.95 |
| ENSG00000109758 | ENSG00000134365 | 0.96 |
| ENSG00000110887 | ENSG00000134365 | 0.95 |
| ENSG00000111181 | ENSG00000134365 | 0.96 |
| ENSG00000113905 | ENSG00000134365 | 0.96 |
| ENSG00000117594 | ENSG00000134365 | 0.95 |
| ENSG00000117601 | ENSG00000134365 | 0.98 |
| ENSG00000118520 | ENSG00000134365 | 0.95 |
| ENSG00000120949 | ENSG00000134365 | 0.95 |
| ENSG00000123561 | ENSG00000134365 | 0.95 |
| ENSG00000126759 | ENSG00000134365 | 0.95 |
| ENSG00000129988 | ENSG00000134365 | 0.96 |
| ENSG00000130649 | ENSG00000134365 | 0.96 |
| ENSG00000132693 | ENSG00000134365 | 0.96 |
| ENSG00000132703 | ENSG00000134365 | 0.98 |
| ENSG00000019169 | ENSG00000134389 | 0.96 |
| ENSG00000039537 | ENSG00000134389 | 0.97 |
| ENSG00000047457 | ENSG00000134389 | 0.95 |
| ENSG00000067057 | ENSG00000134389 | -0.94 |
| ENSG00000072080 | ENSG00000134389 | 0.97 |
| ENSG00000079557 | ENSG00000134389 | 0.95 |
| ENSG00000080910 | ENSG00000134389 | 0.97 |
| ENSG00000102967 | ENSG00000134389 | 0.96 |
| ENSG00000104760 | ENSG00000134389 | 0.98 |
| ENSG00000104938 | ENSG00000134389 | 0.97 |
| ENSG00000105697 | ENSG00000134389 | 0.95 |
| ENSG00000109758 | ENSG00000134389 | 0.97 |
| ENSG00000110169 | ENSG00000134389 | 0.96 |
| ENSG00000113141 | ENSG00000134389 | -0.95 |
| ENSG00000113905 | ENSG00000134389 | 0.95 |
| ENSG00000117594 | ENSG00000134389 | 0.97 |
| ENSG00000117601 | ENSG00000134389 | 0.96 |
| ENSG00000122194 | ENSG00000134389 | 0.96 |
| ENSG00000122787 | ENSG00000134389 | 0.97 |
| ENSG00000126759 | ENSG00000134389 | 0.95 |
| ENSG00000129988 | ENSG00000134389 | 0.95 |
| ENSG00000130649 | ENSG00000134389 | 0.95 |
| ENSG00000132541 | ENSG00000134389 | 0.95 |
| ENSG00000132703 | ENSG00000134389 | 0.97 |
| ENSG00000134365 | ENSG00000134389 | 0.96 |
| ENSG00000087237 | ENSG00000134538 | 0.95 |
| ENSG00000106772 | ENSG00000134548 | -0.94 |
| ENSG00000100889 | ENSG00000134716 | 0.95 |
| ENSG00000087460 | ENSG00000134769 | -0.95 |
| ENSG00000116791 | ENSG00000134779 | -0.97 |
| ENSG00000119537 | ENSG00000134809 | 0.95 |
| ENSG00000126460 | ENSG00000134812 | 0.94 |
| ENSG00000114125 | ENSG00000134905 | 0.95 |
| ENSG00000039537 | ENSG00000135094 | 0.94 |
| ENSG00000072080 | ENSG00000135094 | 0.97 |
| ENSG00000104760 | ENSG00000135094 | 0.96 |
| ENSG00000113905 | ENSG00000135094 | 0.95 |
| ENSG00000115942 | ENSG00000135094 | -0.96 |
| ENSG00000130649 | ENSG00000135094 | 0.95 |
| ENSG00000132703 | ENSG00000135094 | 0.97 |
| ENSG00000134365 | ENSG00000135094 | 0.97 |
| ENSG00000134389 | ENSG00000135094 | 0.95 |
| ENSG00000080493 | ENSG00000135108 | -0.95 |
| ENSG00000133661 | ENSG00000135108 | 0.97 |
| ENSG00000078043 | ENSG00000135111 | -0.96 |
| ENSG00000073350 | ENSG00000135114 | -0.95 |
| ENSG00000132437 | ENSG00000135114 | -0.95 |
| ENSG00000065609 | ENSG00000135164 | 0.96 |
| ENSG00000115648 | ENSG00000135298 | 0.95 |
| ENSG00000006530 | ENSG00000135318 | -0.97 |
| ENSG00000122971 | ENSG00000135318 | 0.94 |
| ENSG00000133135 | ENSG00000135363 | -0.96 |
| ENSG00000067369 | ENSG00000135365 | 0.96 |
| ENSG00000085741 | ENSG00000135374 | 0.96 |
| ENSG00000120697 | ENSG00000135374 | 0.95 |
| ENSG00000124155 | ENSG00000135387 | 0.96 |
| ENSG00000020181 | ENSG00000135406 | 0.95 |
| ENSG00000109084 | ENSG00000135424 | -0.95 |
| ENSG00000117115 | ENSG00000135480 | -0.95 |
| ENSG00000096696 | ENSG00000135525 | 0.97 |
| ENSG00000130675 | ENSG00000135596 | 0.97 |
| ENSG00000103197 | ENSG00000135636 | -0.95 |
| ENSG00000109606 | ENSG00000135636 | -0.95 |
| ENSG00000128283 | ENSG00000135678 | 0.95 |
| ENSG00000007516 | ENSG00000135749 | 0.96 |
| ENSG00000124564 | ENSG00000135801 | 0.95 |
| ENSG00000125462 | ENSG00000135823 | 0.96 |
| ENSG00000111325 | ENSG00000135842 | -0.97 |
| ENSG00000067225 | ENSG00000135924 | 0.94 |
| ENSG00000067836 | ENSG00000135924 | 0.95 |
| ENSG00000069764 | ENSG00000135924 | 0.95 |
| ENSG00000092096 | ENSG00000135924 | 0.96 |
| ENSG00000119906 | ENSG00000135924 | 0.94 |
| ENSG00000135404 | ENSG00000135926 | 0.95 |
| ENSG00000115129 | ENSG00000135956 | 0.96 |
| ENSG00000119431 | ENSG00000135956 | 0.98 |
| ENSG00000089356 | ENSG00000135968 | 0.96 |
| ENSG00000117069 | ENSG00000136040 | 0.96 |
| ENSG00000053371 | ENSG00000136068 | 0.97 |
| ENSG00000021355 | ENSG00000136099 | -0.95 |
| ENSG00000114346 | ENSG00000136108 | 0.94 |
| ENSG00000103024 | ENSG00000136193 | 0.95 |
| ENSG00000105254 | ENSG00000136193 | 0.95 |
| ENSG00000114956 | ENSG00000136193 | 0.95 |
| ENSG00000116675 | ENSG00000136193 | 0.96 |
| ENSG00000090263 | ENSG00000136205 | -0.96 |
| ENSG00000102882 | ENSG00000136238 | 0.95 |
| ENSG00000001497 | ENSG00000136273 | 0.97 |
| ENSG00000077235 | ENSG00000136305 | -0.95 |
| ENSG00000104907 | ENSG00000136305 | -0.96 |
| ENSG00000064692 | ENSG00000136352 | 0.95 |
| ENSG00000072786 | ENSG00000136352 | -0.96 |
| ENSG00000013275 | ENSG00000136367 | -0.95 |
| ENSG00000060709 | ENSG00000136383 | 0.95 |
| ENSG00000109339 | ENSG00000136383 | 0.97 |
| ENSG00000131148 | ENSG00000136404 | -0.95 |
| ENSG00000116898 | ENSG00000136436 | -0.96 |
| ENSG00000112624 | ENSG00000136451 | 0.96 |
| ENSG00000133318 | ENSG00000136451 | 0.96 |
| ENSG00000121905 | ENSG00000136457 | 0.97 |
| ENSG00000103485 | ENSG00000136463 | 0.96 |
| ENSG00000114904 | ENSG00000136463 | -0.95 |
| ENSG00000116580 | ENSG00000136463 | -0.95 |
| ENSG00000132170 | ENSG00000136463 | 0.96 |
| ENSG00000100220 | ENSG00000136535 | -0.97 |
| ENSG00000035115 | ENSG00000136631 | 0.94 |
| ENSG00000076650 | ENSG00000136631 | 0.96 |
| ENSG00000108255 | ENSG00000136754 | -0.94 |
| ENSG00000136108 | ENSG00000136824 | 0.97 |
| ENSG00000005187 | ENSG00000136828 | -0.95 |
| ENSG00000067057 | ENSG00000136828 | 0.96 |
| ENSG00000112282 | ENSG00000136828 | 0.95 |
| ENSG00000125735 | ENSG00000136828 | -0.97 |
| ENSG00000060709 | ENSG00000136842 | -0.94 |
| ENSG00000085511 | ENSG00000136842 | -0.97 |
| ENSG00000112561 | ENSG00000136842 | -0.95 |
| ENSG00000117020 | ENSG00000136842 | -0.96 |
| ENSG00000023839 | ENSG00000136854 | -0.96 |
| ENSG00000060709 | ENSG00000136854 | 0.95 |
| ENSG00000083896 | ENSG00000136854 | 0.96 |
| ENSG00000106089 | ENSG00000136854 | 0.95 |
| ENSG00000106976 | ENSG00000136854 | 0.97 |
| ENSG00000107281 | ENSG00000136854 | 0.96 |
| ENSG00000100196 | ENSG00000136859 | 0.95 |
| ENSG00000086696 | ENSG00000136872 | 0.96 |
| ENSG00000005187 | ENSG00000136881 | 0.95 |
| ENSG00000019169 | ENSG00000136881 | 0.96 |
| ENSG00000039537 | ENSG00000136881 | 0.96 |
| ENSG00000047457 | ENSG00000136881 | 0.96 |
| ENSG00000067057 | ENSG00000136881 | -0.97 |
| ENSG00000080910 | ENSG00000136881 | 0.97 |
| ENSG00000084110 | ENSG00000136881 | 0.96 |
| ENSG00000099937 | ENSG00000136881 | 0.95 |
| ENSG00000100652 | ENSG00000136881 | 0.97 |
| ENSG00000100665 | ENSG00000136881 | 0.96 |
| ENSG00000104760 | ENSG00000136881 | 0.95 |
| ENSG00000104938 | ENSG00000136881 | 0.96 |
| ENSG00000109758 | ENSG00000136881 | 0.95 |
| ENSG00000113141 | ENSG00000136881 | -0.96 |
| ENSG00000113889 | ENSG00000136881 | 0.96 |
| ENSG00000113905 | ENSG00000136881 | 0.96 |
| ENSG00000116678 | ENSG00000136881 | 0.95 |
| ENSG00000116882 | ENSG00000136881 | 0.95 |
| ENSG00000122194 | ENSG00000136881 | 0.97 |
| ENSG00000122787 | ENSG00000136881 | 0.95 |
| ENSG00000123838 | ENSG00000136881 | 0.96 |
| ENSG00000126759 | ENSG00000136881 | 0.94 |
| ENSG00000129988 | ENSG00000136881 | 0.97 |
| ENSG00000134389 | ENSG00000136881 | 0.95 |
| ENSG00000085998 | ENSG00000136888 | 0.95 |
| ENSG00000101843 | ENSG00000136940 | 0.95 |
| ENSG00000104325 | ENSG00000136940 | -0.95 |
| ENSG00000130643 | ENSG00000136960 | 0.95 |
| ENSG00000125656 | ENSG00000136986 | 0.95 |
| ENSG00000131652 | ENSG00000137076 | -0.95 |
| ENSG00000006704 | ENSG00000137100 | 0.96 |
| ENSG00000075239 | ENSG00000137106 | 0.97 |
| ENSG00000132541 | ENSG00000137106 | 0.97 |
| ENSG00000105854 | ENSG00000137161 | -0.96 |
| ENSG00000065609 | ENSG00000137193 | -0.95 |
| ENSG00000113263 | ENSG00000137193 | 0.95 |
| ENSG00000115963 | ENSG00000137409 | -0.95 |
| ENSG00000018236 | ENSG00000137478 | 0.95 |
| ENSG00000039537 | ENSG00000137497 | -0.96 |
| ENSG00000079557 | ENSG00000137497 | -0.95 |
| ENSG00000113889 | ENSG00000137497 | -0.97 |
| ENSG00000102935 | ENSG00000137513 | -0.95 |
| ENSG00000101189 | ENSG00000137574 | 0.95 |
| ENSG00000077522 | ENSG00000137642 | -0.95 |
| ENSG00000052344 | ENSG00000137648 | 0.95 |
| ENSG00000008083 | ENSG00000137700 | -0.95 |
| ENSG00000112818 | ENSG00000137710 | -0.95 |
| ENSG00000113758 | ENSG00000137713 | -0.95 |
| ENSG00000066279 | ENSG00000137804 | 0.95 |
| ENSG00000085719 | ENSG00000137815 | 0.95 |
| ENSG00000123191 | ENSG00000137817 | -0.95 |
| ENSG00000126947 | ENSG00000137817 | 0.96 |
| ENSG00000124209 | ENSG00000137819 | 0.96 |
| ENSG00000091844 | ENSG00000137824 | -0.95 |
| ENSG00000133475 | ENSG00000137824 | 0.96 |
| ENSG00000114812 | ENSG00000137843 | -0.95 |
| ENSG00000065357 | ENSG00000137860 | 0.95 |
| ENSG00000100220 | ENSG00000137860 | 0.96 |
| ENSG00000095713 | ENSG00000137936 | -0.95 |
| ENSG00000113119 | ENSG00000137936 | 0.96 |
| ENSG00000103089 | ENSG00000137960 | 0.95 |
| ENSG00000112297 | ENSG00000137960 | 0.96 |
| ENSG00000120756 | ENSG00000137960 | 0.97 |
| ENSG00000100889 | ENSG00000138030 | 0.95 |
| ENSG00000134716 | ENSG00000138030 | 0.95 |
| ENSG00000010322 | ENSG00000138032 | 0.95 |
| ENSG00000075239 | ENSG00000138074 | 0.95 |
| ENSG00000040199 | ENSG00000138079 | 0.95 |
| ENSG00000122121 | ENSG00000138079 | 0.96 |
| ENSG00000130234 | ENSG00000138079 | 0.97 |
| ENSG00000112245 | ENSG00000138109 | 0.95 |
| ENSG00000105707 | ENSG00000138115 | 0.96 |
| ENSG00000087586 | ENSG00000138160 | 0.98 |
| ENSG00000101003 | ENSG00000138160 | 0.96 |
| ENSG00000112742 | ENSG00000138160 | 0.96 |
| ENSG00000085662 | ENSG00000138175 | 0.95 |
| ENSG00000115163 | ENSG00000138180 | 0.97 |
| ENSG00000133142 | ENSG00000138375 | 0.96 |
| ENSG00000100012 | ENSG00000138435 | 0.96 |
| ENSG00000107779 | ENSG00000138594 | 0.96 |
| ENSG00000129680 | ENSG00000138613 | 0.95 |
| ENSG00000065833 | ENSG00000138622 | 0.96 |
| ENSG00000104888 | ENSG00000138653 | 0.95 |
| ENSG00000106113 | ENSG00000138738 | 0.95 |
| ENSG00000036672 | ENSG00000138757 | 0.95 |
| ENSG00000122121 | ENSG00000138792 | 0.94 |
| ENSG00000033030 | ENSG00000138801 | 0.96 |
| ENSG00000036473 | ENSG00000138823 | 0.94 |
| ENSG00000075643 | ENSG00000138823 | 0.95 |
| ENSG00000136960 | ENSG00000139163 | 0.95 |
| ENSG00000109586 | ENSG00000139209 | -0.95 |
| ENSG00000006128 | ENSG00000139211 | 0.95 |
| ENSG00000115423 | ENSG00000139289 | 0.95 |
| ENSG00000136205 | ENSG00000139292 | -0.95 |
| ENSG00000099785 | ENSG00000139352 | -0.95 |
| ENSG00000075651 | ENSG00000139436 | 0.95 |
| ENSG00000019169 | ENSG00000139547 | 0.96 |
| ENSG00000039537 | ENSG00000139547 | 0.97 |
| ENSG00000067057 | ENSG00000139547 | -0.96 |
| ENSG00000079557 | ENSG00000139547 | 0.95 |
| ENSG00000080910 | ENSG00000139547 | 0.97 |
| ENSG00000084110 | ENSG00000139547 | 0.97 |
| ENSG00000099937 | ENSG00000139547 | 0.95 |
| ENSG00000100665 | ENSG00000139547 | 0.97 |
| ENSG00000104760 | ENSG00000139547 | 0.97 |
| ENSG00000104938 | ENSG00000139547 | 0.96 |
| ENSG00000106927 | ENSG00000139547 | 0.96 |
| ENSG00000109758 | ENSG00000139547 | 0.96 |
| ENSG00000110169 | ENSG00000139547 | 0.95 |
| ENSG00000111181 | ENSG00000139547 | 0.96 |
| ENSG00000113905 | ENSG00000139547 | 0.98 |
| ENSG00000117594 | ENSG00000139547 | 0.96 |
| ENSG00000117601 | ENSG00000139547 | 0.95 |
| ENSG00000122194 | ENSG00000139547 | 0.97 |
| ENSG00000122787 | ENSG00000139547 | 0.95 |
| ENSG00000123838 | ENSG00000139547 | 0.96 |
| ENSG00000126759 | ENSG00000139547 | 0.94 |
| ENSG00000129988 | ENSG00000139547 | 0.99 |
| ENSG00000132693 | ENSG00000139547 | 0.96 |
| ENSG00000132703 | ENSG00000139547 | 0.96 |
| ENSG00000134365 | ENSG00000139547 | 0.96 |
| ENSG00000134389 | ENSG00000139547 | 0.97 |
| ENSG00000135094 | ENSG00000139547 | 0.95 |
| ENSG00000136881 | ENSG00000139547 | 0.98 |
| ENSG00000138778 | ENSG00000139618 | 0.95 |
| ENSG00000100288 | ENSG00000139625 | 0.95 |
| ENSG00000116337 | ENSG00000139625 | 0.94 |
| ENSG00000111481 | ENSG00000139719 | 0.95 |
| ENSG00000117906 | ENSG00000139793 | -0.96 |
| ENSG00000123416 | ENSG00000139793 | -0.95 |
| ENSG00000112561 | ENSG00000139910 | 0.96 |
| ENSG00000115020 | ENSG00000139970 | 0.96 |
| ENSG00000131089 | ENSG00000139970 | 0.95 |
| ENSG00000136108 | ENSG00000140262 | 0.95 |
| ENSG00000079950 | ENSG00000140263 | -0.95 |
| ENSG00000099812 | ENSG00000140297 | 0.97 |
| ENSG00000042445 | ENSG00000140374 | 0.95 |
| ENSG00000123143 | ENSG00000140374 | -0.95 |
| ENSG00000131788 | ENSG00000140374 | -0.95 |
| ENSG00000138029 | ENSG00000140374 | 0.98 |
| ENSG00000122787 | ENSG00000140403 | 0.95 |
| ENSG00000112208 | ENSG00000140416 | 0.95 |
| ENSG00000130176 | ENSG00000140416 | 0.97 |
| ENSG00000133935 | ENSG00000140463 | 0.95 |
| ENSG00000080910 | ENSG00000140505 | 0.98 |
| ENSG00000104760 | ENSG00000140505 | 0.96 |
| ENSG00000109758 | ENSG00000140505 | 0.95 |
| ENSG00000110169 | ENSG00000140505 | 0.97 |
| ENSG00000113600 | ENSG00000140505 | 0.95 |
| ENSG00000113889 | ENSG00000140505 | 0.94 |
| ENSG00000117594 | ENSG00000140505 | 0.95 |
| ENSG00000117601 | ENSG00000140505 | 0.94 |
| ENSG00000126759 | ENSG00000140505 | 0.94 |
| ENSG00000129988 | ENSG00000140505 | 0.96 |
| ENSG00000113441 | ENSG00000140506 | -0.95 |
| ENSG00000128694 | ENSG00000140506 | -0.96 |
| ENSG00000088325 | ENSG00000140525 | 0.96 |
| ENSG00000100526 | ENSG00000140525 | 0.94 |
| ENSG00000134057 | ENSG00000140525 | 0.94 |
| ENSG00000115840 | ENSG00000140575 | 0.95 |
| ENSG00000101335 | ENSG00000140682 | 0.96 |
| ENSG00000069764 | ENSG00000140750 | 0.96 |
| ENSG00000079257 | ENSG00000140750 | 0.95 |
| ENSG00000139719 | ENSG00000140750 | 0.95 |
| ENSG00000081913 | ENSG00000140937 | -0.95 |
| ENSG00000072071 | ENSG00000140939 | 0.95 |
| ENSG00000109472 | ENSG00000140939 | 0.95 |
| ENSG00000137193 | ENSG00000140939 | -0.97 |
| ENSG00000075399 | ENSG00000140941 | 0.94 |
| ENSG00000105198 | ENSG00000140986 | 0.95 |
| ENSG00000100983 | ENSG00000141027 | -0.96 |
| ENSG00000122254 | ENSG00000141027 | 0.96 |
| ENSG00000086827 | ENSG00000141084 | -0.95 |
| ENSG00000112320 | ENSG00000141127 | 0.95 |
| ENSG00000136928 | ENSG00000141127 | 0.95 |
| ENSG00000072832 | ENSG00000141258 | 0.95 |
| ENSG00000103426 | ENSG00000141380 | -0.95 |
| ENSG00000130675 | ENSG00000141380 | -0.95 |
| ENSG00000102409 | ENSG00000141401 | -0.96 |
| ENSG00000100170 | ENSG00000141434 | 0.94 |
| ENSG00000111701 | ENSG00000141434 | 0.95 |
| ENSG00000115474 | ENSG00000141434 | 0.95 |
| ENSG00000117620 | ENSG00000141434 | 0.95 |
| ENSG00000122121 | ENSG00000141434 | 0.99 |
| ENSG00000130234 | ENSG00000141434 | 0.98 |
| ENSG00000138079 | ENSG00000141434 | 0.96 |
| ENSG00000110680 | ENSG00000141448 | -0.95 |
| ENSG00000110048 | ENSG00000141480 | 0.95 |
| ENSG00000122367 | ENSG00000141524 | 0.95 |
| ENSG00000012504 | ENSG00000141556 | -0.95 |
| ENSG00000084674 | ENSG00000141556 | -0.94 |
| ENSG00000111666 | ENSG00000141556 | -0.94 |
| ENSG00000133800 | ENSG00000141556 | -0.95 |
| ENSG00000107562 | ENSG00000141568 | -0.94 |
| ENSG00000112655 | ENSG00000141568 | 0.98 |
| ENSG00000133318 | ENSG00000141568 | 0.97 |
| ENSG00000082068 | ENSG00000141577 | 0.96 |
| ENSG00000135346 | ENSG00000141664 | 0.94 |
| ENSG00000103460 | ENSG00000141753 | -0.94 |
| ENSG00000135052 | ENSG00000141753 | -0.95 |
| ENSG00000011465 | ENSG00000142173 | 0.94 |
| ENSG00000137040 | ENSG00000142192 | 0.95 |
| ENSG00000137409 | ENSG00000142230 | 0.96 |
| ENSG00000099834 | ENSG00000142484 | 0.97 |
| ENSG00000019169 | ENSG00000142494 | 0.97 |
| ENSG00000066813 | ENSG00000142494 | 0.98 |
| ENSG00000072080 | ENSG00000142494 | 0.96 |
| ENSG00000080910 | ENSG00000142494 | 0.96 |
| ENSG00000083807 | ENSG00000142494 | 0.95 |
| ENSG00000100652 | ENSG00000142494 | 0.96 |
| ENSG00000102967 | ENSG00000142494 | 0.97 |
| ENSG00000104760 | ENSG00000142494 | 0.96 |
| ENSG00000104938 | ENSG00000142494 | 0.95 |
| ENSG00000105697 | ENSG00000142494 | 0.95 |
| ENSG00000109758 | ENSG00000142494 | 0.97 |
| ENSG00000119965 | ENSG00000142494 | -0.94 |
| ENSG00000122194 | ENSG00000142494 | 0.96 |
| ENSG00000126759 | ENSG00000142494 | 0.95 |
| ENSG00000128731 | ENSG00000142494 | -0.95 |
| ENSG00000130649 | ENSG00000142494 | 0.97 |
| ENSG00000134389 | ENSG00000142494 | 0.95 |
| ENSG00000136881 | ENSG00000142494 | 0.95 |
| ENSG00000078269 | ENSG00000142675 | 0.95 |
| ENSG00000013588 | ENSG00000142677 | 0.95 |
| ENSG00000075785 | ENSG00000142677 | 0.94 |
| ENSG00000109084 | ENSG00000142677 | -0.96 |
| ENSG00000138172 | ENSG00000142694 | 0.95 |
| ENSG00000081019 | ENSG00000142910 | -0.97 |
| ENSG00000126267 | ENSG00000143106 | 0.95 |
| ENSG00000081237 | ENSG00000143119 | 0.95 |
| ENSG00000117091 | ENSG00000143119 | 0.97 |
| ENSG00000103197 | ENSG00000143126 | 0.95 |
| ENSG00000090402 | ENSG00000143167 | 0.97 |
| ENSG00000108272 | ENSG00000143167 | 0.96 |
| ENSG00000113722 | ENSG00000143167 | 0.96 |
| ENSG00000114455 | ENSG00000143167 | 0.95 |
| ENSG00000010810 | ENSG00000143337 | 0.96 |
| ENSG00000060069 | ENSG00000143337 | 0.95 |
| ENSG00000110955 | ENSG00000143368 | -0.95 |
| ENSG00000112245 | ENSG00000143384 | 0.95 |
| ENSG00000084731 | ENSG00000143409 | 0.95 |
| ENSG00000096093 | ENSG00000143409 | 0.97 |
| ENSG00000101191 | ENSG00000143409 | 0.95 |
| ENSG00000126858 | ENSG00000143409 | 0.95 |
| ENSG00000134851 | ENSG00000143418 | -0.95 |
| ENSG00000129351 | ENSG00000143436 | 0.94 |
| ENSG00000137814 | ENSG00000143442 | 0.96 |
| ENSG00000115561 | ENSG00000143457 | 0.95 |
| ENSG00000138180 | ENSG00000143476 | 0.97 |
| ENSG00000116030 | ENSG00000143499 | -0.96 |
| ENSG00000109919 | ENSG00000143502 | -0.97 |
| ENSG00000133119 | ENSG00000143514 | 0.95 |
| ENSG00000137076 | ENSG00000143514 | -0.97 |
| ENSG00000134825 | ENSG00000143621 | 0.95 |
| ENSG00000042429 | ENSG00000143627 | -0.95 |
| ENSG00000137776 | ENSG00000143627 | -0.95 |
| ENSG00000143171 | ENSG00000143756 | 0.96 |
| ENSG00000063127 | ENSG00000143761 | -0.94 |
| ENSG00000035687 | ENSG00000143797 | 0.96 |
| ENSG00000003147 | ENSG00000143842 | 0.95 |
| ENSG00000111907 | ENSG00000143842 | -0.95 |
| ENSG00000134809 | ENSG00000143842 | -0.95 |
| ENSG00000036565 | ENSG00000143850 | 0.95 |
| ENSG00000100307 | ENSG00000143850 | 0.98 |
| ENSG00000123485 | ENSG00000143919 | 0.95 |
| ENSG00000100351 | ENSG00000144043 | -0.95 |
| ENSG00000059573 | ENSG00000144063 | 0.95 |
| ENSG00000108039 | ENSG00000144063 | 0.97 |
| ENSG00000108272 | ENSG00000144063 | 0.96 |
| ENSG00000130176 | ENSG00000144063 | 0.95 |
| ENSG00000143167 | ENSG00000144063 | 0.97 |
| ENSG00000117602 | ENSG00000144191 | 0.95 |
| ENSG00000089123 | ENSG00000144381 | -0.95 |
| ENSG00000134333 | ENSG00000144381 | 0.95 |
| ENSG00000106128 | ENSG00000144452 | 0.94 |
| ENSG00000132975 | ENSG00000144452 | 0.95 |
| ENSG00000111275 | ENSG00000144524 | -0.96 |
| ENSG00000103266 | ENSG00000144567 | 0.95 |
| ENSG00000074695 | ENSG00000144677 | -0.95 |
| ENSG00000105270 | ENSG00000144677 | 0.95 |
| ENSG00000054356 | ENSG00000144711 | 0.95 |
| ENSG00000111962 | ENSG00000144711 | -0.95 |
| ENSG00000125851 | ENSG00000144711 | 0.97 |
| ENSG00000073417 | ENSG00000144736 | -0.96 |
| ENSG00000141141 | ENSG00000144785 | -0.95 |
| ENSG00000060709 | ENSG00000144834 | 0.96 |
| ENSG00000112561 | ENSG00000144834 | 0.98 |
| ENSG00000117020 | ENSG00000144834 | 0.94 |
| ENSG00000136842 | ENSG00000144834 | -0.95 |
| ENSG00000085563 | ENSG00000144852 | 0.95 |
| ENSG00000112640 | ENSG00000145020 | -0.96 |
| ENSG00000067836 | ENSG00000145022 | 0.96 |
| ENSG00000081041 | ENSG00000145191 | -0.95 |
| ENSG00000117594 | ENSG00000145192 | 0.96 |
| ENSG00000060718 | ENSG00000145246 | 0.95 |
| ENSG00000095203 | ENSG00000145246 | -0.95 |
| ENSG00000139921 | ENSG00000145495 | 0.96 |
| ENSG00000142864 | ENSG00000145604 | 0.96 |
| ENSG00000096433 | ENSG00000145632 | -0.95 |
| ENSG00000019169 | ENSG00000145692 | 0.96 |
| ENSG00000084110 | ENSG00000145692 | 0.95 |
| ENSG00000113905 | ENSG00000145692 | 0.97 |
| ENSG00000130208 | ENSG00000145692 | 0.94 |
| ENSG00000139547 | ENSG00000145692 | 0.95 |
| ENSG00000105290 | ENSG00000145730 | 0.95 |
| ENSG00000106976 | ENSG00000145730 | 0.95 |
| ENSG00000108309 | ENSG00000145730 | 0.97 |
| ENSG00000109472 | ENSG00000145730 | 0.96 |
| ENSG00000111674 | ENSG00000145730 | 0.97 |
| ENSG00000007062 | ENSG00000145781 | 0.95 |
| ENSG00000110651 | ENSG00000145781 | -0.95 |
| ENSG00000019169 | ENSG00000145826 | 0.95 |
| ENSG00000080910 | ENSG00000145826 | 0.94 |
| ENSG00000104760 | ENSG00000145826 | 0.96 |
| ENSG00000105697 | ENSG00000145826 | 0.95 |
| ENSG00000109758 | ENSG00000145826 | 0.97 |
| ENSG00000117594 | ENSG00000145826 | 0.95 |
| ENSG00000122194 | ENSG00000145826 | 0.97 |
| ENSG00000123561 | ENSG00000145826 | 0.98 |
| ENSG00000123838 | ENSG00000145826 | 0.95 |
| ENSG00000128731 | ENSG00000145826 | -0.95 |
| ENSG00000130649 | ENSG00000145826 | 0.95 |
| ENSG00000132840 | ENSG00000145826 | 0.95 |
| ENSG00000134365 | ENSG00000145826 | 0.95 |
| ENSG00000134389 | ENSG00000145826 | 0.95 |
| ENSG00000142494 | ENSG00000145826 | 0.96 |
| ENSG00000032389 | ENSG00000145868 | 0.94 |
| ENSG00000127948 | ENSG00000145882 | -0.96 |
| ENSG00000065320 | ENSG00000145911 | 0.95 |
| ENSG00000103494 | ENSG00000146021 | 0.95 |
| ENSG00000103381 | ENSG00000146039 | 0.95 |
| ENSG00000133135 | ENSG00000146242 | -0.94 |
| ENSG00000140575 | ENSG00000146247 | 0.94 |
| ENSG00000128513 | ENSG00000146463 | 0.95 |
| ENSG00000130208 | ENSG00000146648 | 0.95 |
| ENSG00000087157 | ENSG00000146678 | -0.97 |
| ENSG00000128283 | ENSG00000146678 | 0.98 |
| ENSG00000137497 | ENSG00000146678 | -0.95 |
| ENSG00000105202 | ENSG00000146731 | 0.95 |
| ENSG00000111325 | ENSG00000147100 | 0.95 |
| ENSG00000112640 | ENSG00000147100 | -0.94 |
| ENSG00000131849 | ENSG00000147100 | 0.96 |
| ENSG00000067177 | ENSG00000147130 | 0.96 |
| ENSG00000141837 | ENSG00000147180 | 0.95 |
| ENSG00000127445 | ENSG00000147255 | 0.94 |
| ENSG00000135924 | ENSG00000147416 | 0.95 |
| ENSG00000091583 | ENSG00000147459 | -0.95 |
| ENSG00000091129 | ENSG00000147488 | 0.94 |
| ENSG00000131238 | ENSG00000147488 | 0.96 |
| ENSG00000032389 | ENSG00000147536 | -0.94 |
| ENSG00000131634 | ENSG00000147536 | 0.95 |
| ENSG00000131737 | ENSG00000147588 | 0.95 |
| ENSG00000145692 | ENSG00000147647 | 0.96 |
| ENSG00000101194 | ENSG00000147804 | -0.95 |
| ENSG00000119139 | ENSG00000148082 | -0.94 |
| ENSG00000008735 | ENSG00000148123 | 0.95 |
| ENSG00000058404 | ENSG00000148123 | 0.96 |
| ENSG00000066032 | ENSG00000148123 | 0.95 |
| ENSG00000108852 | ENSG00000148123 | 0.96 |
| ENSG00000120251 | ENSG00000148123 | 0.97 |
| ENSG00000126106 | ENSG00000148123 | 0.95 |
| ENSG00000127252 | ENSG00000148123 | 0.96 |
| ENSG00000132640 | ENSG00000148175 | -0.95 |
| ENSG00000135052 | ENSG00000148175 | -0.96 |
| ENSG00000112343 | ENSG00000148180 | 0.95 |
| ENSG00000083814 | ENSG00000148297 | 0.95 |
| ENSG00000144567 | ENSG00000148297 | 0.96 |
| ENSG00000039537 | ENSG00000148300 | -0.95 |
| ENSG00000047457 | ENSG00000148300 | -0.95 |
| ENSG00000067057 | ENSG00000148300 | 0.98 |
| ENSG00000106804 | ENSG00000148300 | -0.96 |
| ENSG00000112305 | ENSG00000148300 | 0.95 |
| ENSG00000113141 | ENSG00000148300 | 0.97 |
| ENSG00000116478 | ENSG00000148300 | 0.96 |
| ENSG00000119514 | ENSG00000148300 | 0.94 |
| ENSG00000134389 | ENSG00000148300 | -0.95 |
| ENSG00000136828 | ENSG00000148300 | 0.95 |
| ENSG00000070087 | ENSG00000148572 | -0.96 |
| ENSG00000107295 | ENSG00000148572 | -0.96 |
| ENSG00000139546 | ENSG00000148572 | -0.95 |
| ENSG00000130707 | ENSG00000148672 | 0.95 |
| ENSG00000141556 | ENSG00000148672 | -0.95 |
| ENSG00000135094 | ENSG00000148702 | 0.97 |
| ENSG00000090889 | ENSG00000148773 | 0.95 |
| ENSG00000104147 | ENSG00000148773 | 0.95 |
| ENSG00000123094 | ENSG00000148795 | 0.95 |
| ENSG00000008056 | ENSG00000148798 | 0.96 |
| ENSG00000034713 | ENSG00000148798 | 0.96 |
| ENSG00000089199 | ENSG00000148798 | 0.96 |
| ENSG00000104723 | ENSG00000148798 | 0.98 |
| ENSG00000120645 | ENSG00000148798 | 0.95 |
| ENSG00000121671 | ENSG00000148798 | 0.96 |
| ENSG00000133169 | ENSG00000148798 | 0.96 |
| ENSG00000105976 | ENSG00000148834 | 0.97 |
| ENSG00000088002 | ENSG00000148848 | -0.95 |
| ENSG00000102967 | ENSG00000148965 | 0.95 |
| ENSG00000104760 | ENSG00000148965 | 0.96 |
| ENSG00000106327 | ENSG00000148965 | 0.98 |
| ENSG00000115896 | ENSG00000148965 | -0.95 |
| ENSG00000117601 | ENSG00000148965 | 0.95 |
| ENSG00000119514 | ENSG00000148965 | -0.95 |
| ENSG00000126231 | ENSG00000148965 | 0.94 |
| ENSG00000134365 | ENSG00000148965 | 0.95 |
| ENSG00000134389 | ENSG00000148965 | 0.96 |
| ENSG00000134538 | ENSG00000148965 | 0.95 |
| ENSG00000148300 | ENSG00000148965 | -0.96 |
| ENSG00000101746 | ENSG00000149090 | -0.95 |
| ENSG00000075239 | ENSG00000149091 | -0.97 |
| ENSG00000119711 | ENSG00000149091 | -0.94 |
| ENSG00000143457 | ENSG00000149091 | 0.94 |
| ENSG00000005187 | ENSG00000149124 | 0.95 |
| ENSG00000019169 | ENSG00000149124 | 0.97 |
| ENSG00000039537 | ENSG00000149124 | 0.96 |
| ENSG00000047457 | ENSG00000149124 | 0.96 |
| ENSG00000066813 | ENSG00000149124 | 0.96 |
| ENSG00000072080 | ENSG00000149124 | 0.95 |
| ENSG00000080910 | ENSG00000149124 | 0.99 |
| ENSG00000099937 | ENSG00000149124 | 0.96 |
| ENSG00000100652 | ENSG00000149124 | 0.97 |
| ENSG00000102967 | ENSG00000149124 | 0.98 |
| ENSG00000104760 | ENSG00000149124 | 0.97 |
| ENSG00000104938 | ENSG00000149124 | 0.96 |
| ENSG00000105697 | ENSG00000149124 | 0.96 |
| ENSG00000109758 | ENSG00000149124 | 0.98 |
| ENSG00000110169 | ENSG00000149124 | 0.96 |
| ENSG00000111181 | ENSG00000149124 | 0.95 |
| ENSG00000113141 | ENSG00000149124 | -0.96 |
| ENSG00000113889 | ENSG00000149124 | 0.97 |
| ENSG00000113905 | ENSG00000149124 | 0.96 |
| ENSG00000116353 | ENSG00000149124 | -0.96 |
| ENSG00000116882 | ENSG00000149124 | 0.95 |
| ENSG00000117594 | ENSG00000149124 | 0.95 |
| ENSG00000117601 | ENSG00000149124 | 0.96 |
| ENSG00000122194 | ENSG00000149124 | 0.97 |
| ENSG00000122787 | ENSG00000149124 | 0.95 |
| ENSG00000123838 | ENSG00000149124 | 0.94 |
| ENSG00000126759 | ENSG00000149124 | 0.97 |
| ENSG00000129988 | ENSG00000149124 | 0.98 |
| ENSG00000130649 | ENSG00000149124 | 0.96 |
| ENSG00000132703 | ENSG00000149124 | 0.95 |
| ENSG00000134365 | ENSG00000149124 | 0.96 |
| ENSG00000134389 | ENSG00000149124 | 0.96 |
| ENSG00000136881 | ENSG00000149124 | 0.98 |
| ENSG00000139547 | ENSG00000149124 | 0.96 |
| ENSG00000140505 | ENSG00000149124 | 0.96 |
| ENSG00000142494 | ENSG00000149124 | 0.98 |
| ENSG00000145826 | ENSG00000149124 | 0.95 |
| ENSG00000116478 | ENSG00000149150 | -0.96 |
| ENSG00000004487 | ENSG00000149179 | 0.95 |
| ENSG00000081760 | ENSG00000149179 | 0.97 |
| ENSG00000102241 | ENSG00000149179 | 0.95 |
| ENSG00000108666 | ENSG00000149179 | 0.96 |
| ENSG00000119640 | ENSG00000149179 | 0.95 |
| ENSG00000128656 | ENSG00000149179 | 0.96 |
| ENSG00000136928 | ENSG00000149182 | 0.95 |
| ENSG00000117069 | ENSG00000149218 | 0.94 |
| ENSG00000124557 | ENSG00000149305 | 0.95 |
| ENSG00000006530 | ENSG00000149476 | -0.96 |
| ENSG00000135318 | ENSG00000149476 | 0.95 |
| ENSG00000021355 | ENSG00000149485 | -0.96 |
| ENSG00000126787 | ENSG00000149554 | 0.96 |
| ENSG00000101335 | ENSG00000149591 | 0.98 |
| ENSG00000107796 | ENSG00000149591 | 0.94 |
| ENSG00000140682 | ENSG00000149591 | 0.97 |
| ENSG00000130723 | ENSG00000149654 | 0.94 |
| ENSG00000054267 | ENSG00000149929 | 0.95 |
| ENSG00000061273 | ENSG00000149970 | 0.95 |
| ENSG00000105372 | ENSG00000150526 | -0.95 |
| ENSG00000130255 | ENSG00000150526 | -0.96 |
| ENSG00000142534 | ENSG00000150526 | -0.94 |
| ENSG00000006607 | ENSG00000150540 | -0.95 |
| ENSG00000103876 | ENSG00000150540 | 0.95 |
| ENSG00000112365 | ENSG00000150540 | -0.95 |
| ENSG00000067445 | ENSG00000150625 | 0.96 |
| ENSG00000118564 | ENSG00000150625 | 0.96 |
| ENSG00000103184 | ENSG00000151012 | 0.95 |
| ENSG00000126243 | ENSG00000151079 | 0.95 |
| ENSG00000068796 | ENSG00000151224 | -0.96 |
| ENSG00000147804 | ENSG00000151224 | -0.94 |
| ENSG00000055732 | ENSG00000151364 | 0.95 |
| ENSG00000114405 | ENSG00000151376 | 0.95 |
| ENSG00000138801 | ENSG00000151376 | 0.96 |
| ENSG00000096717 | ENSG00000151445 | 0.95 |
| ENSG00000117281 | ENSG00000151445 | -0.94 |
| ENSG00000055957 | ENSG00000151632 | 0.96 |
| ENSG00000110169 | ENSG00000151632 | 0.95 |
| ENSG00000123473 | ENSG00000151632 | -0.95 |
| ENSG00000039537 | ENSG00000151655 | 0.98 |
| ENSG00000080910 | ENSG00000151655 | 0.95 |
| ENSG00000084110 | ENSG00000151655 | 0.96 |
| ENSG00000099937 | ENSG00000151655 | 0.95 |
| ENSG00000104760 | ENSG00000151655 | 0.95 |
| ENSG00000106927 | ENSG00000151655 | 0.96 |
| ENSG00000110887 | ENSG00000151655 | 0.96 |
| ENSG00000111181 | ENSG00000151655 | 0.98 |
| ENSG00000113889 | ENSG00000151655 | 0.95 |
| ENSG00000113905 | ENSG00000151655 | 0.96 |
| ENSG00000117594 | ENSG00000151655 | 0.95 |
| ENSG00000117601 | ENSG00000151655 | 0.97 |
| ENSG00000123838 | ENSG00000151655 | 0.96 |
| ENSG00000129988 | ENSG00000151655 | 0.97 |
| ENSG00000132693 | ENSG00000151655 | 0.97 |
| ENSG00000132703 | ENSG00000151655 | 0.97 |
| ENSG00000134365 | ENSG00000151655 | 0.98 |
| ENSG00000135094 | ENSG00000151655 | 0.95 |
| ENSG00000135744 | ENSG00000151655 | 0.96 |
| ENSG00000136881 | ENSG00000151655 | 0.95 |
| ENSG00000139547 | ENSG00000151655 | 0.97 |
| ENSG00000149124 | ENSG00000151655 | 0.96 |
| ENSG00000077380 | ENSG00000151665 | 0.95 |
| ENSG00000117601 | ENSG00000151665 | -0.94 |
| ENSG00000130675 | ENSG00000151690 | 0.94 |
| ENSG00000112667 | ENSG00000151694 | -0.95 |
| ENSG00000131724 | ENSG00000151725 | -0.95 |
| ENSG00000103966 | ENSG00000151746 | -0.95 |
| ENSG00000108021 | ENSG00000151746 | 0.94 |
| ENSG00000080910 | ENSG00000151790 | 0.95 |
| ENSG00000109758 | ENSG00000151790 | 0.95 |
| ENSG00000110169 | ENSG00000151790 | 0.95 |
| ENSG00000111181 | ENSG00000151790 | 0.96 |
| ENSG00000113889 | ENSG00000151790 | 0.95 |
| ENSG00000117601 | ENSG00000151790 | 0.95 |
| ENSG00000126759 | ENSG00000151790 | 0.97 |
| ENSG00000129988 | ENSG00000151790 | 0.95 |
| ENSG00000132703 | ENSG00000151790 | 0.95 |
| ENSG00000134365 | ENSG00000151790 | 0.95 |
| ENSG00000140505 | ENSG00000151790 | 0.97 |
| ENSG00000149124 | ENSG00000151790 | 0.96 |
| ENSG00000132640 | ENSG00000151929 | -0.94 |
| ENSG00000133878 | ENSG00000151929 | -0.95 |
| ENSG00000148175 | ENSG00000151929 | 0.97 |
| ENSG00000057019 | ENSG00000152104 | 0.95 |
| ENSG00000105974 | ENSG00000152137 | 0.96 |
| ENSG00000105146 | ENSG00000152254 | 0.95 |
| ENSG00000122679 | ENSG00000152256 | -0.95 |
| ENSG00000074755 | ENSG00000152266 | -0.95 |
| ENSG00000100300 | ENSG00000152268 | 0.95 |
| ENSG00000108961 | ENSG00000152284 | -0.97 |
| ENSG00000147168 | ENSG00000152484 | 0.95 |
| ENSG00000107758 | ENSG00000152556 | 0.98 |
| ENSG00000115839 | ENSG00000152556 | 0.95 |
| ENSG00000144677 | ENSG00000152556 | 0.95 |
| ENSG00000149591 | ENSG00000152583 | 0.96 |
| ENSG00000075142 | ENSG00000152642 | 0.97 |
| ENSG00000122359 | ENSG00000152642 | 0.96 |
| ENSG00000084674 | ENSG00000152700 | 0.95 |
| ENSG00000116771 | ENSG00000152700 | 0.96 |
| ENSG00000072518 | ENSG00000152763 | -0.96 |
| ENSG00000146648 | ENSG00000152804 | 0.95 |
| ENSG00000115414 | ENSG00000152952 | 0.95 |
| ENSG00000106541 | ENSG00000152990 | -0.94 |
| ENSG00000115226 | ENSG00000152990 | 0.95 |
| ENSG00000119888 | ENSG00000152990 | -0.95 |
| ENSG00000130787 | ENSG00000152990 | -0.96 |
| ENSG00000132698 | ENSG00000152990 | -0.94 |
| ENSG00000122965 | ENSG00000153037 | 0.95 |
| ENSG00000101138 | ENSG00000153046 | 0.95 |
| ENSG00000011201 | ENSG00000153132 | 0.95 |
| ENSG00000081019 | ENSG00000153140 | 0.95 |
| ENSG00000130826 | ENSG00000153187 | 0.95 |
| ENSG00000072415 | ENSG00000153233 | 0.97 |
| ENSG00000131238 | ENSG00000153253 | 0.96 |
| ENSG00000107438 | ENSG00000153317 | -0.96 |
| ENSG00000101888 | ENSG00000153339 | 0.95 |
| ENSG00000012963 | ENSG00000153395 | 0.95 |
| ENSG00000092201 | ENSG00000153395 | 0.94 |
| ENSG00000112624 | ENSG00000153395 | 0.95 |
| ENSG00000140939 | ENSG00000153558 | 0.96 |
| ENSG00000089091 | ENSG00000153560 | 0.95 |
| ENSG00000123636 | ENSG00000153560 | 0.94 |
| ENSG00000005102 | ENSG00000153574 | 0.95 |
| ENSG00000067445 | ENSG00000153666 | 0.95 |
| ENSG00000150625 | ENSG00000153666 | 0.95 |
| ENSG00000130821 | ENSG00000153774 | 0.95 |
| ENSG00000102001 | ENSG00000153802 | 0.95 |
| ENSG00000082068 | ENSG00000153827 | 0.95 |
| ENSG00000141577 | ENSG00000153827 | 0.96 |
| ENSG00000112624 | ENSG00000153936 | 0.95 |
| ENSG00000115694 | ENSG00000153936 | 0.95 |
| ENSG00000056661 | ENSG00000154146 | 0.97 |
| ENSG00000104327 | ENSG00000154153 | 0.95 |
| ENSG00000128989 | ENSG00000154153 | 0.94 |
| ENSG00000028277 | ENSG00000154165 | 0.95 |
| ENSG00000081041 | ENSG00000154262 | 0.97 |
| ENSG00000065518 | ENSG00000154265 | -0.95 |
| ENSG00000099834 | ENSG00000154274 | 0.95 |
| ENSG00000142484 | ENSG00000154274 | 0.96 |
| ENSG00000077157 | ENSG00000154330 | 0.95 |
| ENSG00000122121 | ENSG00000154330 | 0.95 |
| ENSG00000141434 | ENSG00000154330 | 0.96 |
| ENSG00000147224 | ENSG00000154330 | -0.95 |
| ENSG00000092201 | ENSG00000154473 | 0.95 |
| ENSG00000108468 | ENSG00000154473 | 0.95 |
| ENSG00000133318 | ENSG00000154473 | 0.97 |
| ENSG00000107758 | ENSG00000154710 | 0.97 |
| ENSG00000130714 | ENSG00000154710 | 0.96 |
| ENSG00000124688 | ENSG00000154721 | -0.95 |
| ENSG00000126216 | ENSG00000154721 | -0.94 |
| ENSG00000140374 | ENSG00000154723 | 0.95 |
| ENSG00000105143 | ENSG00000154864 | 0.95 |
| ENSG00000006704 | ENSG00000154978 | 0.95 |
| ENSG00000138107 | ENSG00000154978 | 0.95 |
| ENSG00000105401 | ENSG00000155087 | -0.95 |
| ENSG00000133878 | ENSG00000155093 | 0.97 |
| ENSG00000136842 | ENSG00000155093 | -0.95 |
| ENSG00000147488 | ENSG00000155093 | 0.94 |
| ENSG00000139546 | ENSG00000155130 | 0.95 |
| ENSG00000028310 | ENSG00000155304 | 0.95 |
| ENSG00000108924 | ENSG00000155304 | -0.95 |
| ENSG00000120948 | ENSG00000155304 | 0.95 |
| ENSG00000100324 | ENSG00000155324 | 0.95 |
| ENSG00000139146 | ENSG00000155463 | -0.95 |
| ENSG00000138079 | ENSG00000155465 | 0.94 |
| ENSG00000141002 | ENSG00000155506 | 0.94 |
| ENSG00000138162 | ENSG00000155666 | 0.96 |
| ENSG00000085465 | ENSG00000155760 | -0.95 |
| ENSG00000005187 | ENSG00000155868 | -0.96 |
| ENSG00000019169 | ENSG00000155868 | -0.97 |
| ENSG00000076650 | ENSG00000155868 | 0.95 |
| ENSG00000102967 | ENSG00000155868 | -0.95 |
| ENSG00000102977 | ENSG00000155868 | 0.95 |
| ENSG00000104938 | ENSG00000155868 | -0.96 |
| ENSG00000119965 | ENSG00000155868 | 0.95 |
| ENSG00000122194 | ENSG00000155868 | -0.95 |
| ENSG00000126759 | ENSG00000155868 | -0.94 |
| ENSG00000126858 | ENSG00000155868 | 0.95 |
| ENSG00000129214 | ENSG00000155868 | -0.95 |
| ENSG00000132541 | ENSG00000155868 | -0.95 |
| ENSG00000136631 | ENSG00000155868 | 0.95 |
| ENSG00000143409 | ENSG00000155868 | 0.96 |
| ENSG00000105677 | ENSG00000155876 | 0.95 |
| ENSG00000077238 | ENSG00000155959 | -0.95 |
| ENSG00000086696 | ENSG00000156006 | 0.97 |
| ENSG00000103024 | ENSG00000156030 | 0.95 |
| ENSG00000106976 | ENSG00000156030 | 0.96 |
| ENSG00000135924 | ENSG00000156030 | 0.96 |
| ENSG00000095637 | ENSG00000156222 | 0.96 |
| ENSG00000105398 | ENSG00000156222 | 0.96 |
| ENSG00000110514 | ENSG00000156222 | -0.98 |
| ENSG00000126549 | ENSG00000156261 | -0.96 |
| ENSG00000137561 | ENSG00000156269 | 0.95 |
| ENSG00000105254 | ENSG00000156273 | -0.95 |
| ENSG00000130723 | ENSG00000156273 | -0.95 |
| ENSG00000100417 | ENSG00000156298 | 0.95 |
| ENSG00000119431 | ENSG00000156299 | -0.97 |
| ENSG00000129515 | ENSG00000156313 | -0.97 |
| ENSG00000119681 | ENSG00000156413 | -0.95 |
| ENSG00000143486 | ENSG00000156482 | 0.95 |
| ENSG00000066322 | ENSG00000156642 | 0.97 |
| ENSG00000066813 | ENSG00000156642 | -0.94 |
| ENSG00000072080 | ENSG00000156642 | -0.94 |
| ENSG00000083807 | ENSG00000156642 | -0.95 |
| ENSG00000090054 | ENSG00000156642 | 0.94 |
| ENSG00000117009 | ENSG00000156642 | -0.96 |
| ENSG00000119514 | ENSG00000156642 | 0.94 |
| ENSG00000130649 | ENSG00000156642 | -0.97 |
| ENSG00000135094 | ENSG00000156642 | -0.94 |
| ENSG00000117594 | ENSG00000156711 | -0.95 |
| ENSG00000154262 | ENSG00000156711 | -0.95 |
| ENSG00000100823 | ENSG00000156802 | 0.94 |
| ENSG00000121413 | ENSG00000157064 | 0.96 |
| ENSG00000136717 | ENSG00000157064 | 0.96 |
| ENSG00000154188 | ENSG00000157077 | 0.94 |
| ENSG00000115361 | ENSG00000157103 | 0.95 |
| ENSG00000105379 | ENSG00000157110 | 0.94 |
| ENSG00000085117 | ENSG00000157150 | 0.97 |
| ENSG00000128242 | ENSG00000157150 | 0.96 |
| ENSG00000023839 | ENSG00000157152 | -0.95 |
| ENSG00000105398 | ENSG00000157152 | -0.95 |
| ENSG00000117791 | ENSG00000157152 | -0.95 |
| ENSG00000130723 | ENSG00000157152 | 0.96 |
| ENSG00000147421 | ENSG00000157216 | -0.94 |
| ENSG00000152413 | ENSG00000157240 | 0.96 |
| ENSG00000010322 | ENSG00000157388 | 0.96 |
| ENSG00000134216 | ENSG00000157404 | -0.97 |
| ENSG00000135905 | ENSG00000157450 | -0.94 |
| ENSG00000102393 | ENSG00000157456 | 0.94 |
| ENSG00000126787 | ENSG00000157456 | 0.96 |
| ENSG00000149554 | ENSG00000157456 | 0.96 |
| ENSG00000124702 | ENSG00000157483 | -0.96 |
| ENSG00000135090 | ENSG00000157540 | -0.97 |
| ENSG00000108797 | ENSG00000157554 | 0.95 |
| ENSG00000147548 | ENSG00000157765 | 0.96 |
| ENSG00000110680 | ENSG00000157873 | -0.95 |
| ENSG00000146122 | ENSG00000157978 | 0.95 |
| ENSG00000150995 | ENSG00000158019 | 0.96 |
| ENSG00000044090 | ENSG00000158042 | 0.96 |
| ENSG00000078596 | ENSG00000158050 | 0.96 |
| ENSG00000152953 | ENSG00000158050 | 0.96 |
| ENSG00000019169 | ENSG00000158104 | 0.95 |
| ENSG00000039537 | ENSG00000158104 | 0.96 |
| ENSG00000080910 | ENSG00000158104 | 0.97 |
| ENSG00000104760 | ENSG00000158104 | 0.96 |
| ENSG00000105697 | ENSG00000158104 | 0.98 |
| ENSG00000109758 | ENSG00000158104 | 0.97 |
| ENSG00000110169 | ENSG00000158104 | 0.97 |
| ENSG00000116678 | ENSG00000158104 | 0.94 |
| ENSG00000117594 | ENSG00000158104 | 0.98 |
| ENSG00000117601 | ENSG00000158104 | 0.95 |
| ENSG00000122194 | ENSG00000158104 | 0.95 |
| ENSG00000123561 | ENSG00000158104 | 0.94 |
| ENSG00000123838 | ENSG00000158104 | 0.96 |
| ENSG00000129988 | ENSG00000158104 | 0.96 |
| ENSG00000134365 | ENSG00000158104 | 0.95 |
| ENSG00000134389 | ENSG00000158104 | 0.96 |
| ENSG00000139547 | ENSG00000158104 | 0.95 |
| ENSG00000145826 | ENSG00000158104 | 0.96 |
| ENSG00000149124 | ENSG00000158104 | 0.96 |
| ENSG00000151655 | ENSG00000158104 | 0.95 |
| ENSG00000156711 | ENSG00000158104 | -0.95 |
| ENSG00000103657 | ENSG00000158402 | -0.94 |
| ENSG00000110841 | ENSG00000158445 | 0.95 |
| ENSG00000139182 | ENSG00000158445 | 0.96 |
| ENSG00000074621 | ENSG00000158473 | -0.94 |
| ENSG00000129845 | ENSG00000158477 | 0.94 |
| ENSG00000117602 | ENSG00000158480 | 0.96 |
| ENSG00000101890 | ENSG00000158516 | 0.96 |
| ENSG00000058404 | ENSG00000158560 | 0.95 |
| ENSG00000141505 | ENSG00000158571 | 0.98 |
| ENSG00000116962 | ENSG00000158869 | 0.95 |
| ENSG00000075240 | ENSG00000158874 | 0.97 |
| ENSG00000096395 | ENSG00000158874 | 0.96 |
| ENSG00000111181 | ENSG00000158874 | 0.95 |
| ENSG00000132693 | ENSG00000158874 | 0.95 |
| ENSG00000135744 | ENSG00000158874 | 0.95 |
| ENSG00000151655 | ENSG00000158874 | 0.95 |
| ENSG00000101188 | ENSG00000159131 | 0.96 |
| ENSG00000112182 | ENSG00000159131 | 0.96 |
| ENSG00000117151 | ENSG00000159131 | 0.97 |
| ENSG00000108309 | ENSG00000159164 | 0.95 |
| ENSG00000128563 | ENSG00000159164 | 0.95 |
| ENSG00000149654 | ENSG00000159164 | 0.94 |
| ENSG00000112208 | ENSG00000159176 | 0.95 |
| ENSG00000130176 | ENSG00000159176 | 0.96 |
| ENSG00000140416 | ENSG00000159176 | 0.97 |
| ENSG00000116183 | ENSG00000159184 | 0.95 |
| ENSG00000140320 | ENSG00000159202 | 0.98 |
| ENSG00000087302 | ENSG00000159231 | 0.95 |
| ENSG00000141027 | ENSG00000159231 | -0.98 |
| ENSG00000108953 | ENSG00000159346 | 0.95 |
| ENSG00000090661 | ENSG00000159348 | 0.95 |
| ENSG00000073111 | ENSG00000159403 | -0.96 |
| ENSG00000107562 | ENSG00000159403 | 0.94 |
| ENSG00000108924 | ENSG00000159403 | 0.97 |
| ENSG00000115020 | ENSG00000159409 | 0.97 |
| ENSG00000023228 | ENSG00000159450 | -0.95 |
| ENSG00000157150 | ENSG00000159720 | 0.96 |
| ENSG00000113916 | ENSG00000159842 | -0.98 |
| ENSG00000129596 | ENSG00000159842 | -0.95 |
| ENSG00000067560 | ENSG00000159961 | -0.95 |
| ENSG00000137807 | ENSG00000160199 | 0.95 |
| ENSG00000116141 | ENSG00000160293 | 0.96 |
| ENSG00000130055 | ENSG00000160307 | 0.95 |
| ENSG00000035664 | ENSG00000160325 | -0.96 |
| ENSG00000089876 | ENSG00000160325 | 0.96 |
| ENSG00000127955 | ENSG00000160325 | 0.96 |
| ENSG00000029534 | ENSG00000160460 | 0.95 |
| ENSG00000058404 | ENSG00000160460 | 0.95 |
| ENSG00000102109 | ENSG00000160460 | 0.95 |
| ENSG00000103723 | ENSG00000160460 | 0.97 |
| ENSG00000105290 | ENSG00000160460 | 0.98 |
| ENSG00000106089 | ENSG00000160460 | 0.95 |
| ENSG00000109472 | ENSG00000160460 | 0.94 |
| ENSG00000119630 | ENSG00000160460 | 0.95 |
| ENSG00000130540 | ENSG00000160460 | 0.95 |
| ENSG00000132639 | ENSG00000160460 | 0.96 |
| ENSG00000132718 | ENSG00000160460 | 0.96 |
| ENSG00000101901 | ENSG00000160633 | 0.95 |
| ENSG00000145864 | ENSG00000160654 | 0.96 |
| ENSG00000075142 | ENSG00000160746 | 0.96 |
| ENSG00000076650 | ENSG00000160753 | 0.95 |
| ENSG00000109736 | ENSG00000160753 | 0.95 |
| ENSG00000121691 | ENSG00000160753 | -0.95 |
| ENSG00000137106 | ENSG00000160753 | -0.95 |
| ENSG00000081320 | ENSG00000160783 | -0.95 |
| ENSG00000114784 | ENSG00000160785 | 0.95 |
| ENSG00000141098 | ENSG00000160785 | 0.95 |
| ENSG00000122194 | ENSG00000160796 | -0.94 |
| ENSG00000125735 | ENSG00000160796 | -0.95 |
| ENSG00000134389 | ENSG00000160796 | -0.96 |
| ENSG00000160445 | ENSG00000160796 | 0.95 |
| ENSG00000096395 | ENSG00000160801 | 0.95 |
| ENSG00000134365 | ENSG00000160801 | 0.95 |
| ENSG00000135094 | ENSG00000160801 | 0.95 |
| ENSG00000151655 | ENSG00000160801 | 0.96 |
| ENSG00000128040 | ENSG00000160808 | -0.95 |
| ENSG00000142227 | ENSG00000160808 | 0.95 |
| ENSG00000103248 | ENSG00000160862 | -0.95 |
| ENSG00000147526 | ENSG00000160862 | -0.95 |
| ENSG00000081148 | ENSG00000160867 | 0.94 |
| ENSG00000135823 | ENSG00000160908 | -0.96 |
| ENSG00000139641 | ENSG00000160908 | 0.97 |
| ENSG00000141027 | ENSG00000160948 | -0.95 |
| ENSG00000122254 | ENSG00000160967 | -0.94 |
| ENSG00000139289 | ENSG00000160967 | -0.96 |
| ENSG00000072121 | ENSG00000161010 | 0.96 |
| ENSG00000096093 | ENSG00000161010 | 0.95 |
| ENSG00000143409 | ENSG00000161010 | 0.95 |
| ENSG00000155868 | ENSG00000161010 | 0.95 |
| ENSG00000155465 | ENSG00000161013 | 0.96 |
| ENSG00000115204 | ENSG00000161203 | 0.95 |
| ENSG00000105613 | ENSG00000161270 | 0.95 |
| ENSG00000143631 | ENSG00000161509 | -0.96 |
| ENSG00000107798 | ENSG00000161533 | 0.94 |
| ENSG00000158104 | ENSG00000161572 | -0.94 |
| ENSG00000022267 | ENSG00000161905 | -0.95 |
| ENSG00000151224 | ENSG00000161944 | 0.97 |
| ENSG00000144821 | ENSG00000162066 | -0.96 |
| ENSG00000114395 | ENSG00000162104 | 0.95 |
| ENSG00000105486 | ENSG00000162174 | 0.96 |
| ENSG00000108381 | ENSG00000162174 | -0.97 |
| ENSG00000124613 | ENSG00000162188 | 0.95 |
| ENSG00000076650 | ENSG00000162365 | -0.94 |
| ENSG00000079557 | ENSG00000162365 | 0.97 |
| ENSG00000096654 | ENSG00000162365 | -0.95 |
| ENSG00000101323 | ENSG00000162365 | 0.96 |
| ENSG00000104938 | ENSG00000162365 | 0.94 |
| ENSG00000109758 | ENSG00000162365 | 0.95 |
| ENSG00000110169 | ENSG00000162365 | 0.95 |
| ENSG00000117594 | ENSG00000162365 | 0.95 |
| ENSG00000126759 | ENSG00000162365 | 0.95 |
| ENSG00000126858 | ENSG00000162365 | -0.97 |
| ENSG00000134389 | ENSG00000162365 | 0.95 |
| ENSG00000132640 | ENSG00000162367 | -0.98 |
| ENSG00000077235 | ENSG00000162373 | 0.97 |
| ENSG00000100889 | ENSG00000162373 | -0.96 |
| ENSG00000114405 | ENSG00000162373 | 0.95 |
| ENSG00000136305 | ENSG00000162373 | -0.95 |
| ENSG00000161533 | ENSG00000162373 | -0.95 |
| ENSG00000113384 | ENSG00000162384 | -0.96 |
| ENSG00000130714 | ENSG00000162407 | -0.96 |
| ENSG00000154710 | ENSG00000162407 | -0.96 |
| ENSG00000130675 | ENSG00000162433 | -0.96 |
| ENSG00000106479 | ENSG00000162434 | 0.95 |
| ENSG00000161570 | ENSG00000162434 | -0.95 |
| ENSG00000134716 | ENSG00000162482 | 0.95 |
| ENSG00000138801 | ENSG00000162482 | -0.96 |
| ENSG00000004487 | ENSG00000162517 | 0.96 |
| ENSG00000087237 | ENSG00000162551 | 0.95 |
| ENSG00000149091 | ENSG00000162551 | -0.95 |
| ENSG00000108064 | ENSG00000162552 | -0.95 |
| ENSG00000144711 | ENSG00000162552 | 0.96 |
| ENSG00000106624 | ENSG00000162576 | 0.95 |
| ENSG00000142871 | ENSG00000162576 | 0.97 |
| ENSG00000154330 | ENSG00000162645 | 0.95 |
| ENSG00000102384 | ENSG00000162775 | 0.95 |
| ENSG00000061337 | ENSG00000162817 | -0.94 |
| ENSG00000105655 | ENSG00000162817 | -0.96 |
| ENSG00000155011 | ENSG00000162849 | 0.96 |
| ENSG00000008083 | ENSG00000162873 | 0.95 |
| ENSG00000126106 | ENSG00000162889 | 0.97 |
| ENSG00000104894 | ENSG00000162894 | 0.97 |
| ENSG00000160310 | ENSG00000162928 | -0.94 |
| ENSG00000006611 | ENSG00000162989 | 0.95 |
| ENSG00000129473 | ENSG00000162989 | 0.95 |
| ENSG00000099326 | ENSG00000163013 | 0.96 |
| ENSG00000114405 | ENSG00000163013 | 0.95 |
| ENSG00000116128 | ENSG00000163013 | 0.95 |
| ENSG00000126005 | ENSG00000163050 | 0.95 |
| ENSG00000114770 | ENSG00000163125 | 0.95 |
| ENSG00000158473 | ENSG00000163131 | 0.95 |
| ENSG00000156140 | ENSG00000163132 | 0.95 |
| ENSG00000077809 | ENSG00000163161 | 0.95 |
| ENSG00000137547 | ENSG00000163171 | -0.95 |
| ENSG00000157764 | ENSG00000163191 | -0.94 |
| ENSG00000105143 | ENSG00000163206 | 0.94 |
| ENSG00000116539 | ENSG00000163206 | 0.95 |
| ENSG00000067836 | ENSG00000163214 | 0.95 |
| ENSG00000089053 | ENSG00000163214 | 0.96 |
| ENSG00000136854 | ENSG00000163214 | 0.95 |
| ENSG00000156030 | ENSG00000163214 | 0.96 |
| ENSG00000117151 | ENSG00000163218 | 0.96 |
| ENSG00000105576 | ENSG00000163251 | -0.95 |
| ENSG00000110244 | ENSG00000163295 | 0.96 |
| ENSG00000111701 | ENSG00000163295 | 0.97 |
| ENSG00000102409 | ENSG00000163346 | 0.95 |
| ENSG00000158019 | ENSG00000163346 | 0.95 |
| ENSG00000067606 | ENSG00000163347 | -0.97 |
| ENSG00000016490 | ENSG00000163362 | 0.96 |
| ENSG00000090402 | ENSG00000163362 | 0.97 |
| ENSG00000143167 | ENSG00000163362 | 0.95 |
| ENSG00000144063 | ENSG00000163362 | 0.95 |
| ENSG00000123444 | ENSG00000163374 | 0.95 |
| ENSG00000089472 | ENSG00000163431 | 0.95 |
| ENSG00000114698 | ENSG00000163444 | -0.96 |
| ENSG00000115963 | ENSG00000163479 | -0.97 |
| ENSG00000124766 | ENSG00000163479 | 0.95 |
| ENSG00000019505 | ENSG00000163497 | 0.96 |
| ENSG00000058404 | ENSG00000163497 | 0.95 |
| ENSG00000083799 | ENSG00000163497 | 0.94 |
| ENSG00000102109 | ENSG00000163497 | 0.98 |
| ENSG00000106089 | ENSG00000163497 | 0.96 |
| ENSG00000115020 | ENSG00000163497 | 0.98 |
| ENSG00000120251 | ENSG00000163497 | 0.95 |
| ENSG00000121671 | ENSG00000163497 | 0.97 |
| ENSG00000130643 | ENSG00000163497 | 0.96 |
| ENSG00000132359 | ENSG00000163497 | 0.98 |
| ENSG00000136960 | ENSG00000163497 | 0.95 |
| ENSG00000159409 | ENSG00000163497 | 0.96 |
| ENSG00000160460 | ENSG00000163497 | 0.95 |
| ENSG00000019505 | ENSG00000163499 | 0.95 |
| ENSG00000069020 | ENSG00000163499 | 0.95 |
| ENSG00000100604 | ENSG00000163499 | 0.95 |
| ENSG00000104450 | ENSG00000163499 | 0.95 |
| ENSG00000115020 | ENSG00000163499 | 0.98 |
| ENSG00000120251 | ENSG00000163499 | 0.95 |
| ENSG00000132359 | ENSG00000163499 | 0.96 |
| ENSG00000139970 | ENSG00000163499 | 0.95 |
| ENSG00000159409 | ENSG00000163499 | 0.95 |
| ENSG00000163497 | ENSG00000163499 | 0.97 |
| ENSG00000117724 | ENSG00000163501 | -0.95 |
| ENSG00000130643 | ENSG00000163531 | 0.96 |
| ENSG00000159409 | ENSG00000163531 | 0.95 |
| ENSG00000084731 | ENSG00000163539 | 0.95 |
| ENSG00000060138 | ENSG00000163565 | 0.95 |
| ENSG00000084674 | ENSG00000163581 | 0.98 |
| ENSG00000116771 | ENSG00000163581 | 0.95 |
| ENSG00000131482 | ENSG00000163581 | 0.94 |
| ENSG00000152700 | ENSG00000163581 | 0.94 |
| ENSG00000086696 | ENSG00000163586 | 0.95 |
| ENSG00000136872 | ENSG00000163586 | 0.98 |
| ENSG00000158747 | ENSG00000163624 | 0.97 |
| ENSG00000135094 | ENSG00000163631 | 0.94 |
| ENSG00000087274 | ENSG00000163655 | -0.96 |
| ENSG00000115884 | ENSG00000163687 | 0.95 |
| ENSG00000143158 | ENSG00000163719 | 0.95 |
| ENSG00000103034 | ENSG00000163743 | 0.95 |
| ENSG00000100599 | ENSG00000163781 | -0.94 |
| ENSG00000121031 | ENSG00000163781 | 0.95 |
| ENSG00000127152 | ENSG00000163812 | 0.96 |
| ENSG00000143409 | ENSG00000163814 | 0.95 |
| ENSG00000117151 | ENSG00000163833 | 0.95 |
| ENSG00000135407 | ENSG00000163833 | 0.96 |
| ENSG00000116151 | ENSG00000163874 | 0.94 |
| ENSG00000136504 | ENSG00000163875 | 0.95 |
| ENSG00000157542 | ENSG00000163879 | 0.95 |
| ENSG00000148584 | ENSG00000163918 | -0.96 |
| ENSG00000135486 | ENSG00000163930 | 0.95 |
| ENSG00000106546 | ENSG00000163932 | -0.96 |
| ENSG00000111907 | ENSG00000163932 | -0.95 |
| ENSG00000116299 | ENSG00000163932 | 0.96 |
| ENSG00000134809 | ENSG00000163932 | -0.96 |
| ENSG00000143842 | ENSG00000163932 | 0.96 |
| ENSG00000084731 | ENSG00000163946 | 0.94 |
| ENSG00000118596 | ENSG00000163956 | -0.95 |
| ENSG00000036530 | ENSG00000164010 | 0.96 |
| ENSG00000137936 | ENSG00000164040 | 0.94 |
| ENSG00000099795 | ENSG00000164056 | -0.96 |
| ENSG00000137285 | ENSG00000164068 | -0.96 |
| ENSG00000110925 | ENSG00000164070 | 0.95 |
| ENSG00000136521 | ENSG00000164070 | -0.94 |
| ENSG00000133216 | ENSG00000164078 | 0.94 |
| ENSG00000100652 | ENSG00000164089 | 0.95 |
| ENSG00000113889 | ENSG00000164089 | 0.94 |
| ENSG00000116882 | ENSG00000164089 | 0.95 |
| ENSG00000136881 | ENSG00000164089 | 0.97 |
| ENSG00000006128 | ENSG00000164093 | 0.96 |
| ENSG00000149218 | ENSG00000164093 | 0.96 |
| ENSG00000134057 | ENSG00000164109 | 0.95 |
| ENSG00000139625 | ENSG00000164114 | 0.96 |
| ENSG00000011523 | ENSG00000164120 | -0.95 |
| ENSG00000163586 | ENSG00000164120 | 0.95 |
| ENSG00000114120 | ENSG00000164134 | 0.94 |
| ENSG00000112029 | ENSG00000164151 | 0.95 |
| ENSG00000084652 | ENSG00000164171 | -0.95 |
| ENSG00000137100 | ENSG00000164171 | -0.95 |
| ENSG00000157765 | ENSG00000164175 | 0.95 |
| ENSG00000072422 | ENSG00000164220 | 0.96 |
| ENSG00000091138 | ENSG00000164251 | 0.94 |
| ENSG00000143198 | ENSG00000164251 | 0.95 |
| ENSG00000122711 | ENSG00000164266 | 0.96 |
| ENSG00000120690 | ENSG00000164307 | 0.95 |
| ENSG00000114503 | ENSG00000164344 | -0.95 |
| ENSG00000136960 | ENSG00000164402 | 0.95 |
| ENSG00000162390 | ENSG00000164402 | 0.95 |
| ENSG00000029993 | ENSG00000164458 | 0.97 |
| ENSG00000101216 | ENSG00000164542 | 0.98 |
| ENSG00000088325 | ENSG00000164611 | 0.94 |
| ENSG00000126787 | ENSG00000164611 | 0.96 |
| ENSG00000137078 | ENSG00000164647 | -0.96 |
| ENSG00000124702 | ENSG00000164654 | 0.96 |
| ENSG00000139352 | ENSG00000164654 | 0.96 |
| ENSG00000157483 | ENSG00000164654 | -0.96 |
| ENSG00000046653 | ENSG00000164715 | 0.95 |
| ENSG00000004948 | ENSG00000164761 | 0.95 |
| ENSG00000116353 | ENSG00000164808 | 0.94 |
| ENSG00000090402 | ENSG00000164816 | 0.94 |
| ENSG00000115263 | ENSG00000164816 | 0.95 |
| ENSG00000115386 | ENSG00000164816 | 0.96 |
| ENSG00000134215 | ENSG00000164816 | 0.95 |
| ENSG00000104907 | ENSG00000164818 | 0.95 |
| ENSG00000115263 | ENSG00000164822 | 0.95 |
| ENSG00000115386 | ENSG00000164822 | 0.97 |
| ENSG00000134215 | ENSG00000164822 | 0.96 |
| ENSG00000164816 | ENSG00000164822 | 1 |
| ENSG00000130414 | ENSG00000164823 | -0.97 |
| ENSG00000125877 | ENSG00000164825 | -0.96 |
| ENSG00000152952 | ENSG00000164867 | 0.95 |
| ENSG00000141756 | ENSG00000164879 | -0.94 |
| ENSG00000008517 | ENSG00000164930 | -0.95 |
| ENSG00000012504 | ENSG00000164930 | -0.96 |
| ENSG00000110245 | ENSG00000164930 | -0.95 |
| ENSG00000114771 | ENSG00000164930 | -0.95 |
| ENSG00000125144 | ENSG00000164930 | -0.96 |
| ENSG00000141556 | ENSG00000164930 | 0.95 |
| ENSG00000124444 | ENSG00000164961 | 0.95 |
| ENSG00000135916 | ENSG00000165030 | -0.96 |
| ENSG00000119139 | ENSG00000165092 | 0.97 |
| ENSG00000101442 | ENSG00000165140 | -0.96 |
| ENSG00000112290 | ENSG00000165140 | -0.96 |
| ENSG00000136305 | ENSG00000165140 | 0.95 |
| ENSG00000162373 | ENSG00000165140 | -0.95 |
| ENSG00000105967 | ENSG00000165197 | 0.95 |
| ENSG00000130055 | ENSG00000165197 | 0.95 |
| ENSG00000162777 | ENSG00000165215 | 0.95 |
| ENSG00000163694 | ENSG00000165215 | 0.97 |
| ENSG00000122966 | ENSG00000165219 | 0.95 |
| ENSG00000070501 | ENSG00000165272 | -0.95 |
| ENSG00000099834 | ENSG00000165272 | 0.97 |
| ENSG00000142484 | ENSG00000165272 | 0.98 |
| ENSG00000154274 | ENSG00000165272 | 0.96 |
| ENSG00000106688 | ENSG00000165458 | -0.95 |
| ENSG00000106688 | ENSG00000165475 | 0.97 |
| ENSG00000111666 | ENSG00000165475 | 0.96 |
| ENSG00000165458 | ENSG00000165475 | -0.96 |
| ENSG00000131788 | ENSG00000165487 | 0.97 |
| ENSG00000135315 | ENSG00000165487 | 0.94 |
| ENSG00000163904 | ENSG00000165525 | -0.96 |
| ENSG00000013588 | ENSG00000165556 | 0.95 |
| ENSG00000065361 | ENSG00000165556 | 0.95 |
| ENSG00000079112 | ENSG00000165556 | 0.95 |
| ENSG00000122873 | ENSG00000165556 | 0.97 |
| ENSG00000137710 | ENSG00000165556 | -0.95 |
| ENSG00000152763 | ENSG00000165583 | 0.95 |
| ENSG00000115648 | ENSG00000165646 | 0.94 |
| ENSG00000115461 | ENSG00000165650 | -0.95 |
| ENSG00000107745 | ENSG00000165675 | -0.96 |
| ENSG00000151632 | ENSG00000165682 | 0.94 |
| ENSG00000133878 | ENSG00000165731 | 0.96 |
| ENSG00000112964 | ENSG00000165795 | 0.95 |
| ENSG00000034713 | ENSG00000165801 | 0.94 |
| ENSG00000072041 | ENSG00000165801 | 0.94 |
| ENSG00000108852 | ENSG00000165801 | 0.95 |
| ENSG00000151552 | ENSG00000165801 | 0.95 |
| ENSG00000100170 | ENSG00000165862 | 0.97 |
| ENSG00000111701 | ENSG00000165862 | 0.96 |
| ENSG00000130234 | ENSG00000165862 | 0.96 |
| ENSG00000122034 | ENSG00000165886 | 0.95 |
| ENSG00000105968 | ENSG00000165949 | 0.94 |
| ENSG00000123201 | ENSG00000165973 | 0.95 |
| ENSG00000128683 | ENSG00000165973 | 0.97 |
| ENSG00000135363 | ENSG00000165973 | 0.95 |
| ENSG00000089053 | ENSG00000165983 | -0.96 |
| ENSG00000124762 | ENSG00000166012 | -0.95 |
| ENSG00000142192 | ENSG00000166012 | 0.95 |
| ENSG00000149418 | ENSG00000166033 | -0.96 |
| ENSG00000019169 | ENSG00000166035 | 0.95 |
| ENSG00000039537 | ENSG00000166035 | 0.95 |
| ENSG00000067057 | ENSG00000166035 | -0.95 |
| ENSG00000072080 | ENSG00000166035 | 0.97 |
| ENSG00000080910 | ENSG00000166035 | 0.98 |
| ENSG00000100652 | ENSG00000166035 | 0.96 |
| ENSG00000102967 | ENSG00000166035 | 0.94 |
| ENSG00000104760 | ENSG00000166035 | 0.98 |
| ENSG00000104915 | ENSG00000166035 | -0.95 |
| ENSG00000104938 | ENSG00000166035 | 0.97 |
| ENSG00000109758 | ENSG00000166035 | 0.97 |
| ENSG00000110169 | ENSG00000166035 | 0.95 |
| ENSG00000111181 | ENSG00000166035 | 0.95 |
| ENSG00000113889 | ENSG00000166035 | 0.95 |
| ENSG00000113905 | ENSG00000166035 | 0.94 |
| ENSG00000116882 | ENSG00000166035 | 0.98 |
| ENSG00000117594 | ENSG00000166035 | 0.96 |
| ENSG00000122194 | ENSG00000166035 | 0.97 |
| ENSG00000122787 | ENSG00000166035 | 0.99 |
| ENSG00000126759 | ENSG00000166035 | 0.96 |
| ENSG00000129988 | ENSG00000166035 | 0.97 |
| ENSG00000130649 | ENSG00000166035 | 0.95 |
| ENSG00000132541 | ENSG00000166035 | 0.96 |
| ENSG00000132703 | ENSG00000166035 | 0.97 |
| ENSG00000134365 | ENSG00000166035 | 0.95 |
| ENSG00000134389 | ENSG00000166035 | 0.97 |
| ENSG00000135094 | ENSG00000166035 | 0.95 |
| ENSG00000136881 | ENSG00000166035 | 0.97 |
| ENSG00000139547 | ENSG00000166035 | 0.96 |
| ENSG00000140505 | ENSG00000166035 | 0.96 |
| ENSG00000142494 | ENSG00000166035 | 0.97 |
| ENSG00000145826 | ENSG00000166035 | 0.94 |
| ENSG00000149124 | ENSG00000166035 | 0.97 |
| ENSG00000151790 | ENSG00000166035 | 0.96 |
| ENSG00000132640 | ENSG00000166086 | 0.95 |
| ENSG00000137817 | ENSG00000166135 | 0.97 |
| ENSG00000021355 | ENSG00000166143 | 0.95 |
| ENSG00000149485 | ENSG00000166143 | -0.97 |
| ENSG00000002726 | ENSG00000166145 | 0.97 |
| ENSG00000099812 | ENSG00000166145 | 0.97 |
| ENSG00000134940 | ENSG00000166160 | 0.96 |
| ENSG00000064102 | ENSG00000166173 | 0.96 |
| ENSG00000109339 | ENSG00000166206 | 0.96 |
| ENSG00000132718 | ENSG00000166206 | 0.95 |
| ENSG00000135968 | ENSG00000166224 | 0.95 |
| ENSG00000144848 | ENSG00000166260 | -0.95 |
| ENSG00000127995 | ENSG00000166266 | 0.97 |
| ENSG00000108963 | ENSG00000166313 | 0.96 |
| ENSG00000144283 | ENSG00000166326 | 0.95 |
| ENSG00000124529 | ENSG00000166396 | 0.96 |
| ENSG00000019505 | ENSG00000166398 | 0.95 |
| ENSG00000067445 | ENSG00000166405 | 0.95 |
| ENSG00000118160 | ENSG00000166405 | 0.94 |
| ENSG00000153666 | ENSG00000166405 | 0.95 |
| ENSG00000117115 | ENSG00000166482 | 0.96 |
| ENSG00000118557 | ENSG00000166482 | -0.96 |
| ENSG00000020633 | ENSG00000166501 | 0.96 |
| ENSG00000131730 | ENSG00000166501 | 0.95 |
| ENSG00000074695 | ENSG00000166503 | -0.95 |
| ENSG00000126947 | ENSG00000166503 | 0.95 |
| ENSG00000153666 | ENSG00000166548 | 0.95 |
| ENSG00000126698 | ENSG00000166579 | 0.96 |
| ENSG00000002933 | ENSG00000166619 | -0.95 |
| ENSG00000163221 | ENSG00000166681 | -0.95 |
| ENSG00000005469 | ENSG00000166704 | -0.95 |
| ENSG00000005187 | ENSG00000166747 | -0.95 |
| ENSG00000019169 | ENSG00000166747 | -0.96 |
| ENSG00000104938 | ENSG00000166747 | -0.96 |
| ENSG00000112337 | ENSG00000166747 | -0.94 |
| ENSG00000122194 | ENSG00000166747 | -0.95 |
| ENSG00000067445 | ENSG00000166780 | 0.97 |
| ENSG00000104723 | ENSG00000166780 | 0.96 |
| ENSG00000148798 | ENSG00000166780 | 0.97 |
| ENSG00000150625 | ENSG00000166780 | 0.95 |
| ENSG00000153666 | ENSG00000166780 | 0.95 |
| ENSG00000166548 | ENSG00000166780 | 0.95 |
| ENSG00000010219 | ENSG00000166783 | 0.95 |
| ENSG00000114742 | ENSG00000166783 | 0.95 |
| ENSG00000119965 | ENSG00000166787 | -0.95 |
| ENSG00000115263 | ENSG00000166825 | 0.95 |
| ENSG00000132326 | ENSG00000166833 | 0.94 |
| ENSG00000101199 | ENSG00000166848 | 0.96 |
| ENSG00000130540 | ENSG00000166848 | 0.95 |
| ENSG00000138279 | ENSG00000166848 | 0.96 |
| ENSG00000108187 | ENSG00000166863 | 0.95 |
| ENSG00000013588 | ENSG00000166866 | 0.95 |
| ENSG00000112818 | ENSG00000166866 | 0.96 |
| ENSG00000137710 | ENSG00000166866 | -0.96 |
| ENSG00000090402 | ENSG00000166869 | 0.95 |
| ENSG00000101213 | ENSG00000166869 | 0.94 |
| ENSG00000108272 | ENSG00000166869 | 0.96 |
| ENSG00000115474 | ENSG00000166869 | 0.95 |
| ENSG00000130234 | ENSG00000166869 | 0.94 |
| ENSG00000143167 | ENSG00000166869 | 0.97 |
| ENSG00000144063 | ENSG00000166869 | 0.96 |
| ENSG00000164816 | ENSG00000166869 | 0.95 |
| ENSG00000164822 | ENSG00000166869 | 0.95 |
| ENSG00000165272 | ENSG00000166881 | -0.95 |
| ENSG00000008056 | ENSG00000166922 | 0.96 |
| ENSG00000074695 | ENSG00000166922 | -0.95 |
| ENSG00000099949 | ENSG00000166922 | 0.95 |
| ENSG00000104723 | ENSG00000166922 | 0.95 |
| ENSG00000120645 | ENSG00000166922 | 0.96 |
| ENSG00000132639 | ENSG00000166922 | 0.95 |
| ENSG00000133169 | ENSG00000166922 | 0.99 |
| ENSG00000148798 | ENSG00000166922 | 0.95 |
| ENSG00000115204 | ENSG00000166971 | 0.95 |
| ENSG00000136754 | ENSG00000166971 | 0.95 |
| ENSG00000154133 | ENSG00000166971 | -0.95 |
| ENSG00000136870 | ENSG00000166986 | 0.94 |
| ENSG00000162711 | ENSG00000166997 | 0.97 |
| ENSG00000100079 | ENSG00000167117 | 0.96 |
| ENSG00000102934 | ENSG00000167130 | 0.95 |
| ENSG00000064763 | ENSG00000167183 | 0.96 |
| ENSG00000106025 | ENSG00000167183 | 0.95 |
| ENSG00000127831 | ENSG00000167183 | 0.95 |
| ENSG00000123091 | ENSG00000167193 | -0.95 |
| ENSG00000141127 | ENSG00000167193 | -0.95 |
| ENSG00000165731 | ENSG00000167193 | -0.97 |
| ENSG00000135052 | ENSG00000167244 | -0.96 |
| ENSG00000155304 | ENSG00000167244 | -0.95 |
| ENSG00000155307 | ENSG00000167286 | 0.96 |
| ENSG00000164128 | ENSG00000167286 | -0.95 |
| ENSG00000132780 | ENSG00000167325 | 0.95 |
| ENSG00000085741 | ENSG00000167332 | 0.95 |
| ENSG00000120697 | ENSG00000167332 | 0.97 |
| ENSG00000124935 | ENSG00000167332 | 0.97 |
| ENSG00000135374 | ENSG00000167332 | 0.96 |
| ENSG00000105866 | ENSG00000167363 | 0.95 |
| ENSG00000006607 | ENSG00000167378 | 0.95 |
| ENSG00000120910 | ENSG00000167378 | 0.95 |
| ENSG00000162517 | ENSG00000167378 | 0.95 |
| ENSG00000056661 | ENSG00000167393 | 0.95 |
| ENSG00000108840 | ENSG00000167393 | 0.95 |
| ENSG00000165006 | ENSG00000167434 | 0.96 |
| ENSG00000007944 | ENSG00000167487 | 0.95 |
| ENSG00000157077 | ENSG00000167526 | 0.94 |
| ENSG00000072121 | ENSG00000167535 | 0.96 |
| ENSG00000079102 | ENSG00000167535 | 0.95 |
| ENSG00000103024 | ENSG00000167535 | 0.96 |
| ENSG00000106976 | ENSG00000167535 | 0.96 |
| ENSG00000111674 | ENSG00000167535 | 0.97 |
| ENSG00000114956 | ENSG00000167535 | 0.96 |
| ENSG00000116675 | ENSG00000167535 | 0.98 |
| ENSG00000136193 | ENSG00000167535 | 0.97 |
| ENSG00000156030 | ENSG00000167535 | 0.96 |
| ENSG00000101323 | ENSG00000167555 | -0.97 |
| ENSG00000006607 | ENSG00000167578 | 0.96 |
| ENSG00000107185 | ENSG00000167578 | 0.95 |
| ENSG00000013583 | ENSG00000167588 | 0.94 |
| ENSG00000122971 | ENSG00000167635 | -0.95 |
| ENSG00000129596 | ENSG00000167642 | -0.99 |
| ENSG00000159842 | ENSG00000167642 | 0.96 |
| ENSG00000068971 | ENSG00000167685 | 0.95 |
| ENSG00000108840 | ENSG00000167685 | 0.95 |
| ENSG00000127252 | ENSG00000167685 | 0.95 |
| ENSG00000167393 | ENSG00000167685 | 0.95 |
| ENSG00000140093 | ENSG00000167711 | 0.97 |
| ENSG00000122718 | ENSG00000167751 | 0.95 |
| ENSG00000163833 | ENSG00000167751 | 0.95 |
| ENSG00000101365 | ENSG00000167754 | -0.95 |
| ENSG00000065183 | ENSG00000167759 | 0.95 |
| ENSG00000135407 | ENSG00000167759 | 0.95 |
| ENSG00000164961 | ENSG00000167770 | 0.95 |
| ENSG00000100462 | ENSG00000167775 | 0.95 |
| ENSG00000165169 | ENSG00000167780 | -0.95 |
| ENSG00000132434 | ENSG00000167792 | 0.95 |
| ENSG00000108509 | ENSG00000167862 | -0.95 |
| ENSG00000069018 | ENSG00000167900 | -0.96 |
| ENSG00000011021 | ENSG00000167964 | 0.94 |
| ENSG00000047249 | ENSG00000167965 | 0.98 |
| ENSG00000117791 | ENSG00000167965 | -0.96 |
| ENSG00000136193 | ENSG00000167965 | 0.96 |
| ENSG00000157152 | ENSG00000167965 | 0.96 |
| ENSG00000108309 | ENSG00000167972 | 0.95 |
| ENSG00000108395 | ENSG00000167972 | 0.95 |
| ENSG00000145730 | ENSG00000167972 | 0.95 |
| ENSG00000128731 | ENSG00000167986 | 0.95 |
| ENSG00000004487 | ENSG00000168000 | 0.96 |
| ENSG00000047249 | ENSG00000168000 | 0.95 |
| ENSG00000135365 | ENSG00000168000 | 0.94 |
| ENSG00000149179 | ENSG00000168000 | 0.95 |
| ENSG00000167965 | ENSG00000168000 | 0.97 |
| ENSG00000105404 | ENSG00000168002 | 0.96 |
| ENSG00000157191 | ENSG00000168002 | 0.95 |
| ENSG00000123143 | ENSG00000168014 | 0.95 |
| ENSG00000138029 | ENSG00000168014 | -0.97 |
| ENSG00000006744 | ENSG00000168061 | 0.97 |
| ENSG00000090889 | ENSG00000168078 | 0.95 |
| ENSG00000113387 | ENSG00000168078 | 0.97 |
| ENSG00000114346 | ENSG00000168078 | 0.95 |
| ENSG00000164611 | ENSG00000168078 | 0.97 |
| ENSG00000138085 | ENSG00000168124 | -0.95 |
| ENSG00000132680 | ENSG00000168137 | 0.95 |
| ENSG00000064309 | ENSG00000168175 | -0.94 |
| ENSG00000136960 | ENSG00000168209 | 0.96 |
| ENSG00000163171 | ENSG00000168209 | 0.95 |
| ENSG00000164402 | ENSG00000168209 | 0.95 |
| ENSG00000147168 | ENSG00000168229 | 0.95 |
| ENSG00000121807 | ENSG00000168259 | -0.96 |
| ENSG00000119471 | ENSG00000168268 | -0.95 |
| ENSG00000078295 | ENSG00000168280 | 0.96 |
| ENSG00000157542 | ENSG00000168280 | 0.95 |
| ENSG00000074621 | ENSG00000168283 | 0.96 |
| ENSG00000106479 | ENSG00000168283 | 0.96 |
| ENSG00000158473 | ENSG00000168283 | -0.97 |
| ENSG00000133835 | ENSG00000168288 | 0.95 |
| ENSG00000146083 | ENSG00000168288 | -0.95 |
| ENSG00000108578 | ENSG00000168291 | 0.95 |
| ENSG00000158869 | ENSG00000168291 | -0.95 |
| ENSG00000110002 | ENSG00000168386 | 0.95 |
| ENSG00000086589 | ENSG00000168476 | 0.95 |
| ENSG00000102109 | ENSG00000168476 | 0.95 |
| ENSG00000105063 | ENSG00000168476 | 0.98 |
| ENSG00000137207 | ENSG00000168496 | 0.95 |
| ENSG00000115306 | ENSG00000168497 | 0.96 |
| ENSG00000133401 | ENSG00000168522 | 0.95 |
| ENSG00000119139 | ENSG00000168542 | -0.94 |
| ENSG00000164692 | ENSG00000168542 | 0.94 |
| ENSG00000168397 | ENSG00000168546 | -0.96 |
| ENSG00000131981 | ENSG00000168765 | 0.96 |
| ENSG00000143842 | ENSG00000168772 | 0.96 |
| ENSG00000138061 | ENSG00000168785 | 0.95 |
| ENSG00000124688 | ENSG00000168795 | 0.96 |
| ENSG00000075239 | ENSG00000168887 | -0.97 |
| ENSG00000076650 | ENSG00000168887 | 0.94 |
| ENSG00000083720 | ENSG00000168887 | 0.95 |
| ENSG00000106804 | ENSG00000168887 | -0.96 |
| ENSG00000108666 | ENSG00000168887 | 0.97 |
| ENSG00000119711 | ENSG00000168887 | -0.95 |
| ENSG00000132541 | ENSG00000168887 | -0.97 |
| ENSG00000149091 | ENSG00000168887 | 0.95 |
| ENSG00000163794 | ENSG00000168903 | -0.96 |
| ENSG00000108654 | ENSG00000168906 | -0.95 |
| ENSG00000137675 | ENSG00000168928 | 0.95 |
| ENSG00000076108 | ENSG00000168955 | -0.95 |
| ENSG00000108669 | ENSG00000169006 | -0.95 |
| ENSG00000154864 | ENSG00000169006 | 0.95 |
| ENSG00000075213 | ENSG00000169047 | 0.96 |
| ENSG00000125850 | ENSG00000169047 | -0.95 |
| ENSG00000116353 | ENSG00000169057 | 0.95 |
| ENSG00000146122 | ENSG00000169071 | 0.96 |
| ENSG00000105492 | ENSG00000169083 | 0.96 |
| ENSG00000064547 | ENSG00000169100 | 0.94 |
| ENSG00000117859 | ENSG00000169100 | -0.96 |
| ENSG00000151552 | ENSG00000169116 | 0.95 |
| ENSG00000165801 | ENSG00000169116 | 0.96 |
| ENSG00000005187 | ENSG00000169136 | 0.95 |
| ENSG00000019169 | ENSG00000169136 | 0.96 |
| ENSG00000102967 | ENSG00000169136 | 0.96 |
| ENSG00000143819 | ENSG00000169136 | 0.94 |
| ENSG00000155868 | ENSG00000169136 | -0.96 |
| ENSG00000136504 | ENSG00000169213 | 0.95 |
| ENSG00000103024 | ENSG00000169217 | 0.98 |
| ENSG00000116044 | ENSG00000169217 | -0.95 |
| ENSG00000125744 | ENSG00000169217 | 0.96 |
| ENSG00000151079 | ENSG00000169217 | 0.95 |
| ENSG00000099977 | ENSG00000169221 | -0.96 |
| ENSG00000122641 | ENSG00000169231 | 0.95 |
| ENSG00000104888 | ENSG00000169297 | 0.96 |
| ENSG00000138653 | ENSG00000169297 | 0.95 |
| ENSG00000112855 | ENSG00000169371 | 0.96 |
| ENSG00000070081 | ENSG00000169372 | -0.97 |
| ENSG00000141424 | ENSG00000169398 | 0.96 |
| ENSG00000164754 | ENSG00000169398 | 0.95 |
| ENSG00000168306 | ENSG00000169398 | -0.95 |
| ENSG00000015285 | ENSG00000169429 | 0.94 |
| ENSG00000102109 | ENSG00000169567 | -0.95 |
| ENSG00000163497 | ENSG00000169567 | -0.94 |
| ENSG00000134853 | ENSG00000169679 | -0.97 |
| ENSG00000111344 | ENSG00000169692 | -0.98 |
| ENSG00000125148 | ENSG00000169715 | 0.96 |
| ENSG00000105404 | ENSG00000169727 | 0.96 |
| ENSG00000167775 | ENSG00000169727 | 0.94 |
| ENSG00000132768 | ENSG00000169855 | 0.96 |
| ENSG00000146013 | ENSG00000169855 | 0.97 |
| ENSG00000092036 | ENSG00000169857 | 0.96 |
| ENSG00000104903 | ENSG00000169860 | 0.97 |
| ENSG00000100604 | ENSG00000169862 | 0.97 |
| ENSG00000104450 | ENSG00000169862 | 0.95 |
| ENSG00000115020 | ENSG00000169862 | 0.95 |
| ENSG00000163499 | ENSG00000169862 | 0.96 |
| ENSG00000077044 | ENSG00000169902 | -0.94 |
| ENSG00000130988 | ENSG00000169902 | 0.95 |
| ENSG00000163374 | ENSG00000169906 | -0.96 |
| ENSG00000095059 | ENSG00000169976 | 0.98 |
| ENSG00000165140 | ENSG00000170004 | -0.96 |
| ENSG00000118804 | ENSG00000170017 | -0.95 |
| ENSG00000132670 | ENSG00000170035 | 0.96 |
| ENSG00000144199 | ENSG00000170035 | -0.95 |
| ENSG00000168546 | ENSG00000170075 | 0.94 |
| ENSG00000101213 | ENSG00000170231 | 0.95 |
| ENSG00000164626 | ENSG00000170231 | 0.96 |
| ENSG00000058404 | ENSG00000170233 | 0.95 |
| ENSG00000102109 | ENSG00000170233 | 0.97 |
| ENSG00000106089 | ENSG00000170233 | 0.95 |
| ENSG00000115020 | ENSG00000170233 | 0.95 |
| ENSG00000119630 | ENSG00000170233 | 0.96 |
| ENSG00000120645 | ENSG00000170233 | 0.95 |
| ENSG00000121671 | ENSG00000170233 | 0.96 |
| ENSG00000132359 | ENSG00000170233 | 0.96 |
| ENSG00000132639 | ENSG00000170233 | 0.96 |
| ENSG00000132718 | ENSG00000170233 | 0.97 |
| ENSG00000160460 | ENSG00000170233 | 0.98 |
| ENSG00000163497 | ENSG00000170233 | 0.97 |
| ENSG00000166206 | ENSG00000170233 | 0.94 |
| ENSG00000137204 | ENSG00000170265 | -0.95 |
| ENSG00000135407 | ENSG00000170289 | 0.96 |
| ENSG00000071991 | ENSG00000170296 | -0.95 |
| ENSG00000090889 | ENSG00000170312 | 0.94 |
| ENSG00000114346 | ENSG00000170312 | 0.95 |
| ENSG00000131747 | ENSG00000170312 | 0.95 |
| ENSG00000134057 | ENSG00000170312 | 0.95 |
| ENSG00000164109 | ENSG00000170312 | 0.95 |
| ENSG00000164611 | ENSG00000170312 | 0.96 |
| ENSG00000168078 | ENSG00000170312 | 0.96 |
| ENSG00000124935 | ENSG00000170345 | -0.95 |
| ENSG00000128268 | ENSG00000170369 | -0.95 |
| ENSG00000078699 | ENSG00000170430 | -0.95 |
| ENSG00000105379 | ENSG00000170430 | 0.95 |
| ENSG00000105229 | ENSG00000170442 | -0.95 |
| ENSG00000141646 | ENSG00000170456 | -0.95 |
| ENSG00000055957 | ENSG00000170458 | 0.95 |
| ENSG00000109519 | ENSG00000170458 | 0.95 |
| ENSG00000104880 | ENSG00000170545 | 0.95 |
| ENSG00000128789 | ENSG00000170579 | 0.95 |
| ENSG00000075673 | ENSG00000170604 | 0.95 |
| ENSG00000112561 | ENSG00000170633 | 0.95 |
| ENSG00000126217 | ENSG00000170633 | 0.95 |
| ENSG00000166851 | ENSG00000170634 | -0.94 |
| ENSG00000054356 | ENSG00000170745 | 0.96 |
| ENSG00000066032 | ENSG00000170745 | 0.96 |
| ENSG00000072041 | ENSG00000170745 | 0.97 |
| ENSG00000073969 | ENSG00000170745 | 0.95 |
| ENSG00000103154 | ENSG00000170745 | 0.95 |
| ENSG00000118898 | ENSG00000170745 | 0.97 |
| ENSG00000127252 | ENSG00000170745 | 0.96 |
| ENSG00000165731 | ENSG00000170745 | 0.96 |
| ENSG00000095485 | ENSG00000170820 | -0.97 |
| ENSG00000063176 | ENSG00000170848 | -0.96 |
| ENSG00000064270 | ENSG00000170871 | 0.95 |
| ENSG00000023608 | ENSG00000170921 | 0.94 |
| ENSG00000064205 | ENSG00000170921 | 0.96 |
| ENSG00000106123 | ENSG00000170961 | 0.95 |
| ENSG00000116157 | ENSG00000170961 | 0.95 |
| ENSG00000116984 | ENSG00000170962 | 0.94 |
| ENSG00000085063 | ENSG00000171130 | 0.95 |
| ENSG00000109832 | ENSG00000171130 | 0.95 |
| ENSG00000117151 | ENSG00000171132 | 0.95 |
| ENSG00000139985 | ENSG00000171132 | 0.94 |
| ENSG00000091527 | ENSG00000171150 | -0.95 |
| ENSG00000121281 | ENSG00000171161 | -0.95 |
| ENSG00000110696 | ENSG00000171174 | -0.95 |
| ENSG00000165475 | ENSG00000171174 | 0.95 |
| ENSG00000105146 | ENSG00000171201 | 0.95 |
| ENSG00000114786 | ENSG00000171222 | 0.95 |
| ENSG00000160180 | ENSG00000171302 | 0.94 |
| ENSG00000064607 | ENSG00000171303 | 0.95 |
| ENSG00000114405 | ENSG00000171303 | 0.94 |
| ENSG00000111339 | ENSG00000171345 | -0.97 |
| ENSG00000133119 | ENSG00000171365 | -0.94 |
| ENSG00000122711 | ENSG00000171431 | 0.98 |
| ENSG00000164266 | ENSG00000171431 | 0.95 |
| ENSG00000102109 | ENSG00000171450 | 0.97 |
| ENSG00000104112 | ENSG00000171450 | 0.95 |
| ENSG00000106089 | ENSG00000171450 | 0.95 |
| ENSG00000117133 | ENSG00000171453 | 0.96 |
| ENSG00000089199 | ENSG00000171476 | 0.95 |
| ENSG00000102109 | ENSG00000171476 | 0.95 |
| ENSG00000115020 | ENSG00000171476 | 0.94 |
| ENSG00000121671 | ENSG00000171476 | 0.97 |
| ENSG00000130643 | ENSG00000171476 | 0.97 |
| ENSG00000131089 | ENSG00000171476 | 0.96 |
| ENSG00000136960 | ENSG00000171476 | 0.94 |
| ENSG00000139970 | ENSG00000171476 | 0.95 |
| ENSG00000159409 | ENSG00000171476 | 0.94 |
| ENSG00000163497 | ENSG00000171476 | 0.96 |
| ENSG00000163531 | ENSG00000171476 | 0.96 |
| ENSG00000135945 | ENSG00000171492 | 0.95 |
| ENSG00000006530 | ENSG00000171503 | -0.96 |
| ENSG00000116514 | ENSG00000171522 | 0.96 |
| ENSG00000129675 | ENSG00000171551 | 0.95 |
| ENSG00000137252 | ENSG00000171551 | 0.94 |
| ENSG00000163288 | ENSG00000171551 | 0.96 |
| ENSG00000147459 | ENSG00000171557 | -0.95 |
| ENSG00000156096 | ENSG00000171557 | 0.95 |
| ENSG00000111181 | ENSG00000171560 | 0.95 |
| ENSG00000143845 | ENSG00000171560 | 0.96 |
| ENSG00000134480 | ENSG00000171564 | 0.95 |
| ENSG00000139289 | ENSG00000171564 | 0.95 |
| ENSG00000004487 | ENSG00000171603 | 0.94 |
| ENSG00000126858 | ENSG00000171603 | 0.95 |
| ENSG00000137497 | ENSG00000171603 | 0.94 |
| ENSG00000149179 | ENSG00000171603 | 0.97 |
| ENSG00000081177 | ENSG00000171634 | 0.94 |
| ENSG00000135577 | ENSG00000171681 | -0.95 |
| ENSG00000132702 | ENSG00000171720 | -0.94 |
| ENSG00000163481 | ENSG00000171720 | 0.94 |
| ENSG00000006128 | ENSG00000171735 | 0.94 |
| ENSG00000055732 | ENSG00000171735 | 0.95 |
| ENSG00000151364 | ENSG00000171735 | 0.98 |
| ENSG00000079112 | ENSG00000171747 | 0.96 |
| ENSG00000124570 | ENSG00000171747 | 0.97 |
| ENSG00000127831 | ENSG00000171747 | 0.96 |
| ENSG00000003436 | ENSG00000171766 | 0.96 |
| ENSG00000168000 | ENSG00000171766 | -0.96 |
| ENSG00000034713 | ENSG00000171793 | -0.96 |
| ENSG00000108852 | ENSG00000171793 | -0.96 |
| ENSG00000120251 | ENSG00000171793 | -0.96 |
| ENSG00000165801 | ENSG00000171793 | -0.96 |
| ENSG00000019169 | ENSG00000171824 | -0.94 |
| ENSG00000099937 | ENSG00000171824 | -0.95 |
| ENSG00000155868 | ENSG00000171824 | 0.95 |
| ENSG00000067369 | ENSG00000171843 | 0.94 |
| ENSG00000106692 | ENSG00000171843 | 0.95 |
| ENSG00000135365 | ENSG00000171843 | 0.94 |
| ENSG00000139719 | ENSG00000171853 | 0.95 |
| ENSG00000171551 | ENSG00000171873 | 0.95 |
| ENSG00000113552 | ENSG00000171936 | -0.96 |
| ENSG00000074319 | ENSG00000171940 | -0.95 |
| ENSG00000075213 | ENSG00000171940 | 0.95 |
| ENSG00000167081 | ENSG00000171940 | -0.94 |
| ENSG00000078295 | ENSG00000171951 | 0.96 |
| ENSG00000102109 | ENSG00000171951 | 0.95 |
| ENSG00000109339 | ENSG00000171951 | 0.95 |
| ENSG00000171450 | ENSG00000171951 | 0.95 |
| ENSG00000111845 | ENSG00000171953 | 0.94 |
| ENSG00000006744 | ENSG00000171960 | 0.95 |
| ENSG00000123342 | ENSG00000171989 | 0.96 |
| ENSG00000130779 | ENSG00000171992 | 0.94 |
| ENSG00000164816 | ENSG00000172016 | 0.97 |
| ENSG00000164822 | ENSG00000172016 | 0.98 |
| ENSG00000063177 | ENSG00000172061 | 0.95 |
| ENSG00000136213 | ENSG00000172113 | -0.95 |
| ENSG00000021300 | ENSG00000172216 | -0.95 |
| ENSG00000117620 | ENSG00000172238 | 0.96 |
| ENSG00000118777 | ENSG00000172238 | 0.95 |
| ENSG00000122121 | ENSG00000172238 | 0.96 |
| ENSG00000138792 | ENSG00000172238 | 0.96 |
| ENSG00000141434 | ENSG00000172238 | 0.97 |
| ENSG00000147224 | ENSG00000172238 | -0.95 |
| ENSG00000154330 | ENSG00000172238 | 0.97 |
| ENSG00000129625 | ENSG00000172262 | -0.97 |
| ENSG00000118508 | ENSG00000172269 | -0.95 |
| ENSG00000129007 | ENSG00000172283 | -0.96 |
| ENSG00000048740 | ENSG00000172292 | 0.95 |
| ENSG00000070019 | ENSG00000172292 | 0.96 |
| ENSG00000100441 | ENSG00000172292 | 0.95 |
| ENSG00000137563 | ENSG00000172292 | -0.97 |
| ENSG00000136305 | ENSG00000172340 | 0.95 |
| ENSG00000148672 | ENSG00000172340 | 0.95 |
| ENSG00000087086 | ENSG00000172345 | 0.95 |
| ENSG00000138796 | ENSG00000172345 | 0.95 |
| ENSG00000036565 | ENSG00000172348 | 0.96 |
| ENSG00000089199 | ENSG00000172348 | 0.94 |
| ENSG00000108852 | ENSG00000172348 | 0.97 |
| ENSG00000120251 | ENSG00000172348 | 0.96 |
| ENSG00000126106 | ENSG00000172348 | 0.96 |
| ENSG00000165801 | ENSG00000172348 | 0.97 |
| ENSG00000171793 | ENSG00000172348 | -0.95 |
| ENSG00000142583 | ENSG00000172367 | 0.96 |
| ENSG00000060709 | ENSG00000172379 | 0.98 |
| ENSG00000070444 | ENSG00000172379 | 0.95 |
| ENSG00000085511 | ENSG00000172379 | 0.97 |
| ENSG00000102109 | ENSG00000172379 | 0.95 |
| ENSG00000112561 | ENSG00000172379 | 0.95 |
| ENSG00000132359 | ENSG00000172379 | 0.96 |
| ENSG00000136960 | ENSG00000172379 | 0.97 |
| ENSG00000144834 | ENSG00000172379 | 0.94 |
| ENSG00000078699 | ENSG00000172432 | 0.94 |
| ENSG00000170430 | ENSG00000172432 | -0.95 |
| ENSG00000058404 | ENSG00000172478 | 0.95 |
| ENSG00000120251 | ENSG00000172478 | 0.96 |
| ENSG00000127252 | ENSG00000172478 | 0.96 |
| ENSG00000136383 | ENSG00000172478 | 0.95 |
| ENSG00000148123 | ENSG00000172478 | 0.96 |
| ENSG00000170233 | ENSG00000172478 | 0.95 |
| ENSG00000171793 | ENSG00000172478 | -0.95 |
| ENSG00000019169 | ENSG00000172482 | 0.96 |
| ENSG00000080910 | ENSG00000172482 | 0.96 |
| ENSG00000084110 | ENSG00000172482 | 0.96 |
| ENSG00000096654 | ENSG00000172482 | -0.96 |
| ENSG00000099937 | ENSG00000172482 | 0.95 |
| ENSG00000100652 | ENSG00000172482 | 0.97 |
| ENSG00000100665 | ENSG00000172482 | 0.96 |
| ENSG00000104938 | ENSG00000172482 | 0.96 |
| ENSG00000113905 | ENSG00000172482 | 0.95 |
| ENSG00000122194 | ENSG00000172482 | 0.96 |
| ENSG00000123838 | ENSG00000172482 | 0.95 |
| ENSG00000129988 | ENSG00000172482 | 0.95 |
| ENSG00000136881 | ENSG00000172482 | 0.98 |
| ENSG00000139547 | ENSG00000172482 | 0.96 |
| ENSG00000145692 | ENSG00000172482 | 0.95 |
| ENSG00000149124 | ENSG00000172482 | 0.95 |
| ENSG00000164089 | ENSG00000172482 | 0.96 |
| ENSG00000166747 | ENSG00000172482 | -0.95 |
| ENSG00000127415 | ENSG00000172531 | -0.96 |
| ENSG00000069020 | ENSG00000172575 | 0.96 |
| ENSG00000104450 | ENSG00000172575 | 0.96 |
| ENSG00000115020 | ENSG00000172575 | 0.97 |
| ENSG00000120251 | ENSG00000172575 | 0.96 |
| ENSG00000159409 | ENSG00000172575 | 0.95 |
| ENSG00000163499 | ENSG00000172575 | 0.97 |
| ENSG00000169862 | ENSG00000172575 | 0.95 |
| ENSG00000129195 | ENSG00000172663 | 0.95 |
| ENSG00000132170 | ENSG00000172766 | -0.95 |
| ENSG00000150526 | ENSG00000172809 | -0.94 |
| ENSG00000163810 | ENSG00000172817 | 0.96 |
| ENSG00000082512 | ENSG00000172830 | 0.95 |
| ENSG00000134365 | ENSG00000172830 | -0.95 |
| ENSG00000042445 | ENSG00000172831 | 0.95 |
| ENSG00000105784 | ENSG00000172831 | 0.94 |
| ENSG00000085788 | ENSG00000172845 | 0.95 |
| ENSG00000124233 | ENSG00000172845 | 0.95 |
| ENSG00000130035 | ENSG00000172845 | -0.94 |
| ENSG00000013275 | ENSG00000172867 | -0.95 |
| ENSG00000120875 | ENSG00000172915 | 0.96 |
| ENSG00000162367 | ENSG00000172915 | -0.96 |
| ENSG00000106460 | ENSG00000172936 | -0.95 |
| ENSG00000138080 | ENSG00000172943 | -0.95 |
| ENSG00000141469 | ENSG00000172955 | 0.95 |
| ENSG00000149792 | ENSG00000172977 | 0.96 |
| ENSG00000015475 | ENSG00000173064 | -0.95 |
| ENSG00000106479 | ENSG00000173064 | 0.95 |
| ENSG00000031823 | ENSG00000173156 | -0.96 |
| ENSG00000103876 | ENSG00000173156 | 0.96 |
| ENSG00000110583 | ENSG00000173156 | -0.94 |
| ENSG00000117461 | ENSG00000173156 | -0.95 |
| ENSG00000134013 | ENSG00000173198 | 0.96 |
| ENSG00000103381 | ENSG00000173207 | -0.96 |
| ENSG00000139405 | ENSG00000173218 | -0.95 |
| ENSG00000156006 | ENSG00000173221 | 0.95 |
| ENSG00000003147 | ENSG00000173404 | 0.97 |
| ENSG00000117594 | ENSG00000173432 | 0.95 |
| ENSG00000138356 | ENSG00000173432 | 0.96 |
| ENSG00000145192 | ENSG00000173432 | 0.95 |
| ENSG00000156711 | ENSG00000173432 | -0.94 |
| ENSG00000158104 | ENSG00000173432 | 0.95 |
| ENSG00000143502 | ENSG00000173597 | -0.95 |
| ENSG00000163257 | ENSG00000173660 | -0.95 |
| ENSG00000094914 | ENSG00000173692 | 0.95 |
| ENSG00000163743 | ENSG00000173692 | 0.94 |
| ENSG00000091490 | ENSG00000173801 | 0.95 |
| ENSG00000114529 | ENSG00000173801 | 0.95 |
| ENSG00000166145 | ENSG00000173801 | 0.95 |
| ENSG00000149292 | ENSG00000173826 | 0.95 |
| ENSG00000130988 | ENSG00000173848 | -0.95 |
| ENSG00000156675 | ENSG00000173848 | 0.96 |
| ENSG00000149150 | ENSG00000173862 | 0.95 |
| ENSG00000161526 | ENSG00000173862 | -0.95 |
| ENSG00000125861 | ENSG00000173918 | 0.97 |
| ENSG00000139656 | ENSG00000173933 | -0.97 |
| ENSG00000122970 | ENSG00000174156 | -0.95 |
| ENSG00000172345 | ENSG00000174156 | 0.95 |
| ENSG00000111674 | ENSG00000174373 | 0.95 |
| ENSG00000164144 | ENSG00000174437 | 0.95 |
| ENSG00000143793 | ENSG00000174640 | -0.95 |
| ENSG00000100234 | ENSG00000174744 | -0.96 |
| ENSG00000074590 | ENSG00000174748 | 0.95 |
| ENSG00000111275 | ENSG00000174775 | -0.95 |
| ENSG00000148572 | ENSG00000174775 | -0.95 |
| ENSG00000134905 | ENSG00000174808 | 0.95 |
| ENSG00000167377 | ENSG00000174915 | 0.97 |
| ENSG00000130723 | ENSG00000174938 | 0.96 |
| ENSG00000130985 | ENSG00000174938 | 0.96 |
| ENSG00000157152 | ENSG00000174938 | 0.96 |
| ENSG00000072832 | ENSG00000174943 | 0.95 |
| ENSG00000103723 | ENSG00000174943 | 0.96 |
| ENSG00000104112 | ENSG00000174943 | 0.95 |
| ENSG00000173702 | ENSG00000174944 | 0.96 |
| ENSG00000105290 | ENSG00000174946 | -0.95 |
| ENSG00000132718 | ENSG00000174946 | -0.94 |
| ENSG00000167632 | ENSG00000174946 | -0.95 |
| ENSG00000137221 | ENSG00000174977 | 0.95 |
| ENSG00000115263 | ENSG00000174992 | 0.97 |
| ENSG00000166825 | ENSG00000174992 | 0.96 |
| ENSG00000023839 | ENSG00000174996 | -0.95 |
| ENSG00000078295 | ENSG00000174996 | 0.96 |
| ENSG00000109472 | ENSG00000174996 | 0.96 |
| ENSG00000116128 | ENSG00000174996 | 0.95 |
| ENSG00000168280 | ENSG00000174996 | 0.94 |
| ENSG00000100652 | ENSG00000175003 | 0.96 |
| ENSG00000136881 | ENSG00000175003 | 0.97 |
| ENSG00000143845 | ENSG00000175003 | 0.94 |
| ENSG00000164089 | ENSG00000175003 | 0.97 |
| ENSG00000172482 | ENSG00000175003 | 0.97 |
| ENSG00000151725 | ENSG00000175063 | 0.95 |
| ENSG00000102445 | ENSG00000175084 | 0.95 |
| ENSG00000108272 | ENSG00000175084 | 0.95 |
| ENSG00000131981 | ENSG00000175084 | 0.95 |
| ENSG00000144063 | ENSG00000175084 | 0.95 |
| ENSG00000168765 | ENSG00000175084 | 0.96 |
| ENSG00000132912 | ENSG00000175110 | -0.95 |
| ENSG00000105254 | ENSG00000175130 | 0.95 |
| ENSG00000163875 | ENSG00000175130 | 0.95 |
| ENSG00000095397 | ENSG00000175182 | 0.97 |
| ENSG00000116783 | ENSG00000175182 | 0.95 |
| ENSG00000139625 | ENSG00000175182 | 0.96 |
| ENSG00000080910 | ENSG00000175189 | 0.95 |
| ENSG00000105707 | ENSG00000175189 | 0.95 |
| ENSG00000106927 | ENSG00000175189 | 0.95 |
| ENSG00000111181 | ENSG00000175189 | 0.97 |
| ENSG00000117594 | ENSG00000175189 | 0.97 |
| ENSG00000117601 | ENSG00000175189 | 0.97 |
| ENSG00000129988 | ENSG00000175189 | 0.95 |
| ENSG00000130649 | ENSG00000175189 | 0.94 |
| ENSG00000138115 | ENSG00000175189 | 0.96 |
| ENSG00000145192 | ENSG00000175189 | 0.95 |
| ENSG00000151655 | ENSG00000175189 | 0.96 |
| ENSG00000125170 | ENSG00000175264 | -0.96 |
| ENSG00000136352 | ENSG00000175264 | 0.96 |
| ENSG00000136352 | ENSG00000175305 | 0.96 |
| ENSG00000156298 | ENSG00000175305 | -0.94 |
| ENSG00000112651 | ENSG00000175352 | 0.97 |
| ENSG00000144837 | ENSG00000175356 | -0.95 |
| ENSG00000136059 | ENSG00000175416 | 0.95 |
| ENSG00000172283 | ENSG00000175416 | -0.95 |
| ENSG00000173702 | ENSG00000175416 | 0.95 |
| ENSG00000123091 | ENSG00000175426 | 0.95 |
| ENSG00000133878 | ENSG00000175426 | 0.96 |
| ENSG00000136842 | ENSG00000175426 | -0.95 |
| ENSG00000141380 | ENSG00000175426 | -0.95 |
| ENSG00000165731 | ENSG00000175426 | 0.96 |
| ENSG00000170745 | ENSG00000175426 | 0.95 |
| ENSG00000165801 | ENSG00000175581 | -0.95 |
| ENSG00000169116 | ENSG00000175581 | -0.96 |
| ENSG00000072042 | ENSG00000175591 | -0.95 |
| ENSG00000123243 | ENSG00000175745 | -0.95 |
| ENSG00000104907 | ENSG00000175792 | 0.95 |
| ENSG00000062725 | ENSG00000175806 | -0.95 |
| ENSG00000106688 | ENSG00000175806 | 0.96 |
| ENSG00000109472 | ENSG00000175806 | -0.95 |
| ENSG00000114631 | ENSG00000175806 | -0.94 |
| ENSG00000152217 | ENSG00000175806 | -0.95 |
| ENSG00000174946 | ENSG00000175806 | 0.94 |
| ENSG00000174996 | ENSG00000175806 | -0.94 |
| ENSG00000166780 | ENSG00000175832 | 0.95 |
| ENSG00000034713 | ENSG00000175866 | 0.96 |
| ENSG00000115020 | ENSG00000175866 | 0.96 |
| ENSG00000120251 | ENSG00000175866 | 0.97 |
| ENSG00000159409 | ENSG00000175866 | 0.95 |
| ENSG00000163499 | ENSG00000175866 | 0.96 |
| ENSG00000163531 | ENSG00000175866 | 0.95 |
| ENSG00000171793 | ENSG00000175866 | -0.95 |
| ENSG00000172348 | ENSG00000175866 | 0.95 |
| ENSG00000172575 | ENSG00000175866 | 0.95 |
| ENSG00000164600 | ENSG00000175895 | -0.95 |
| ENSG00000117592 | ENSG00000175906 | 0.94 |
| ENSG00000107331 | ENSG00000175931 | 0.95 |
| ENSG00000139625 | ENSG00000175931 | 0.96 |
| ENSG00000140543 | ENSG00000175931 | 0.95 |
| ENSG00000175182 | ENSG00000175931 | 0.95 |
| ENSG00000121413 | ENSG00000175938 | 0.96 |
| ENSG00000157064 | ENSG00000175938 | 0.97 |
| ENSG00000054793 | ENSG00000175970 | 0.95 |
| ENSG00000092096 | ENSG00000175970 | 0.95 |
| ENSG00000107758 | ENSG00000175970 | 0.96 |
| ENSG00000163214 | ENSG00000175970 | 0.95 |
| ENSG00000105971 | ENSG00000176014 | 0.95 |
| ENSG00000162434 | ENSG00000176049 | 0.96 |
| ENSG00000154262 | ENSG00000176101 | -0.96 |
| ENSG00000095713 | ENSG00000176105 | 0.95 |
| ENSG00000141542 | ENSG00000176108 | 0.97 |
| ENSG00000006047 | ENSG00000176165 | 0.95 |
| ENSG00000160193 | ENSG00000176170 | 0.95 |
| ENSG00000145041 | ENSG00000176276 | 0.95 |
| ENSG00000145868 | ENSG00000176340 | 0.95 |
| ENSG00000103241 | ENSG00000176387 | 0.97 |
| ENSG00000120162 | ENSG00000176387 | 0.97 |
| ENSG00000135622 | ENSG00000176406 | 0.97 |
| ENSG00000131788 | ENSG00000176444 | 0.94 |
| ENSG00000136305 | ENSG00000176444 | -0.96 |
| ENSG00000148672 | ENSG00000176444 | -0.95 |
| ENSG00000168268 | ENSG00000176444 | 0.95 |
| ENSG00000172340 | ENSG00000176444 | -0.97 |
| ENSG00000065183 | ENSG00000176454 | -0.96 |
| ENSG00000083799 | ENSG00000176623 | -0.95 |
| ENSG00000108852 | ENSG00000176623 | -0.96 |
| ENSG00000162174 | ENSG00000176734 | 0.96 |
| ENSG00000169398 | ENSG00000176734 | 0.95 |
| ENSG00000038358 | ENSG00000176783 | 0.95 |
| ENSG00000143507 | ENSG00000176783 | -0.95 |
| ENSG00000143819 | ENSG00000176783 | -0.94 |
| ENSG00000175416 | ENSG00000176834 | 0.95 |
| ENSG00000131844 | ENSG00000176884 | 0.96 |
| ENSG00000088325 | ENSG00000176890 | 0.95 |
| ENSG00000111206 | ENSG00000176890 | 0.95 |
| ENSG00000126787 | ENSG00000176890 | 0.97 |
| ENSG00000164611 | ENSG00000176890 | 0.96 |
| ENSG00000170312 | ENSG00000176890 | 0.97 |
| ENSG00000055118 | ENSG00000176894 | -0.95 |
| ENSG00000104112 | ENSG00000176894 | -0.95 |
| ENSG00000106976 | ENSG00000176894 | -0.95 |
| ENSG00000108309 | ENSG00000176894 | -0.95 |
| ENSG00000109472 | ENSG00000176894 | -0.96 |
| ENSG00000111674 | ENSG00000176894 | -0.98 |
| ENSG00000116128 | ENSG00000176894 | -0.96 |
| ENSG00000145730 | ENSG00000176894 | -0.98 |
| ENSG00000167535 | ENSG00000176894 | -0.96 |
| ENSG00000171450 | ENSG00000176894 | -0.96 |
| ENSG00000172345 | ENSG00000176894 | 0.95 |
| ENSG00000174996 | ENSG00000176894 | -0.95 |
| ENSG00000164961 | ENSG00000176919 | -0.95 |
| ENSG00000174243 | ENSG00000176919 | -0.95 |
| ENSG00000137177 | ENSG00000176953 | 0.94 |
| ENSG00000067836 | ENSG00000176956 | 0.95 |
| ENSG00000141127 | ENSG00000176956 | 0.95 |
| ENSG00000165943 | ENSG00000176956 | 0.97 |
| ENSG00000102109 | ENSG00000176974 | -0.97 |
| ENSG00000107281 | ENSG00000176974 | -0.96 |
| ENSG00000169567 | ENSG00000176974 | 0.95 |
| ENSG00000171450 | ENSG00000176974 | -0.95 |
| ENSG00000154099 | ENSG00000177042 | -0.96 |
| ENSG00000167244 | ENSG00000177054 | -0.95 |
| ENSG00000090661 | ENSG00000177105 | -0.94 |
| ENSG00000126005 | ENSG00000177106 | 0.95 |
| ENSG00000167085 | ENSG00000177156 | 0.95 |
| ENSG00000118564 | ENSG00000177189 | -0.94 |
| ENSG00000101191 | ENSG00000177380 | 0.96 |
| ENSG00000111642 | ENSG00000177380 | 0.96 |
| ENSG00000112096 | ENSG00000177380 | -0.95 |
| ENSG00000086589 | ENSG00000177383 | 0.95 |
| ENSG00000105254 | ENSG00000177383 | 0.96 |
| ENSG00000125744 | ENSG00000177383 | 0.97 |
| ENSG00000175130 | ENSG00000177383 | 0.96 |
| ENSG00000001084 | ENSG00000177425 | 0.95 |
| ENSG00000076716 | ENSG00000177426 | 0.94 |
| ENSG00000131171 | ENSG00000177548 | 0.96 |
| ENSG00000159184 | ENSG00000177551 | 0.98 |
| ENSG00000004534 | ENSG00000177575 | -0.97 |
| ENSG00000137100 | ENSG00000177628 | 0.95 |
| ENSG00000149182 | ENSG00000177628 | 0.97 |
| ENSG00000164171 | ENSG00000177628 | -0.95 |
| ENSG00000169271 | ENSG00000177628 | -0.95 |
| ENSG00000152700 | ENSG00000177707 | 0.96 |
| ENSG00000154743 | ENSG00000177854 | 0.95 |
| ENSG00000154274 | ENSG00000177981 | 0.95 |
| ENSG00000160182 | ENSG00000178078 | 0.96 |
| ENSG00000133884 | ENSG00000178104 | 0.95 |
| ENSG00000163913 | ENSG00000178104 | 0.95 |
| ENSG00000174231 | ENSG00000178104 | 0.95 |
| ENSG00000105655 | ENSG00000178146 | -0.96 |
| ENSG00000131773 | ENSG00000178146 | -0.95 |
| ENSG00000058600 | ENSG00000178188 | 0.95 |
| ENSG00000174405 | ENSG00000178201 | 0.95 |
| ENSG00000104112 | ENSG00000178252 | 0.95 |
| ENSG00000108309 | ENSG00000178252 | 0.95 |
| ENSG00000145730 | ENSG00000178252 | 0.95 |
| ENSG00000149654 | ENSG00000178252 | 0.95 |
| ENSG00000099785 | ENSG00000178287 | -0.94 |
| ENSG00000135116 | ENSG00000178363 | 0.95 |
| ENSG00000120910 | ENSG00000178394 | -0.97 |
| ENSG00000122729 | ENSG00000178394 | 0.96 |
| ENSG00000171606 | ENSG00000178394 | 0.94 |
| ENSG00000068078 | ENSG00000178401 | 0.96 |
| ENSG00000099937 | ENSG00000178445 | 0.95 |
| ENSG00000129214 | ENSG00000178445 | 0.95 |
| ENSG00000171824 | ENSG00000178445 | -0.97 |
| ENSG00000085063 | ENSG00000178467 | 0.95 |
| ENSG00000102109 | ENSG00000178467 | 0.96 |
| ENSG00000105290 | ENSG00000178467 | 0.96 |
| ENSG00000106089 | ENSG00000178467 | 0.97 |
| ENSG00000106976 | ENSG00000178467 | 0.95 |
| ENSG00000132718 | ENSG00000178467 | 0.95 |
| ENSG00000136854 | ENSG00000178467 | 0.95 |
| ENSG00000160460 | ENSG00000178467 | 0.95 |
| ENSG00000166206 | ENSG00000178467 | 0.95 |
| ENSG00000171450 | ENSG00000178467 | 0.96 |
| ENSG00000166780 | ENSG00000178498 | 0.96 |
| ENSG00000106688 | ENSG00000178537 | 0.95 |
| ENSG00000156006 | ENSG00000178537 | 0.97 |
| ENSG00000165475 | ENSG00000178537 | 0.94 |
| ENSG00000141002 | ENSG00000178538 | -0.94 |
| ENSG00000146950 | ENSG00000178573 | -0.96 |
| ENSG00000089472 | ENSG00000178623 | 0.95 |
| ENSG00000161813 | ENSG00000178672 | 0.95 |
| ENSG00000081019 | ENSG00000178691 | 0.96 |
| ENSG00000153140 | ENSG00000178691 | 0.95 |
| ENSG00000036565 | ENSG00000178695 | 0.97 |
| ENSG00000060709 | ENSG00000178695 | 0.94 |
| ENSG00000069020 | ENSG00000178695 | 0.94 |
| ENSG00000100604 | ENSG00000178695 | 0.94 |
| ENSG00000115020 | ENSG00000178695 | 0.97 |
| ENSG00000120251 | ENSG00000178695 | 0.97 |
| ENSG00000139970 | ENSG00000178695 | 0.96 |
| ENSG00000162433 | ENSG00000178695 | -0.95 |
| ENSG00000163497 | ENSG00000178695 | 0.95 |
| ENSG00000163499 | ENSG00000178695 | 0.98 |
| ENSG00000172575 | ENSG00000178695 | 0.95 |
| ENSG00000132321 | ENSG00000178719 | 0.97 |
| ENSG00000150995 | ENSG00000178828 | 0.96 |
| ENSG00000076650 | ENSG00000178860 | 0.95 |
| ENSG00000048649 | ENSG00000178913 | 0.95 |
| ENSG00000072080 | ENSG00000178950 | -0.97 |
| ENSG00000089234 | ENSG00000178950 | 0.94 |
| ENSG00000115942 | ENSG00000178950 | 0.96 |
| ENSG00000135094 | ENSG00000178950 | -0.96 |
| ENSG00000142494 | ENSG00000178950 | -0.96 |
| ENSG00000136108 | ENSG00000178966 | 0.97 |
| ENSG00000136824 | ENSG00000178966 | 0.97 |
| ENSG00000115216 | ENSG00000178971 | 0.94 |
| ENSG00000131508 | ENSG00000178980 | 0.96 |
| ENSG00000126267 | ENSG00000178982 | 0.95 |
| ENSG00000108352 | ENSG00000179087 | 0.95 |
| ENSG00000122121 | ENSG00000179087 | 0.95 |
| ENSG00000130234 | ENSG00000179087 | 0.95 |
| ENSG00000141434 | ENSG00000179087 | 0.96 |
| ENSG00000077984 | ENSG00000179091 | 0.95 |
| ENSG00000126752 | ENSG00000179091 | -0.95 |
| ENSG00000171132 | ENSG00000179097 | 0.95 |
| ENSG00000115963 | ENSG00000179115 | -0.95 |
| ENSG00000168439 | ENSG00000179115 | 0.95 |
| ENSG00000169255 | ENSG00000179222 | 0.97 |
| ENSG00000105697 | ENSG00000179271 | -0.95 |
| ENSG00000169180 | ENSG00000179449 | 0.95 |
| ENSG00000122873 | ENSG00000179604 | 0.95 |
| ENSG00000015413 | ENSG00000179674 | 0.96 |
| ENSG00000021488 | ENSG00000179674 | 0.95 |
| ENSG00000130821 | ENSG00000179674 | 0.95 |
| ENSG00000133392 | ENSG00000179674 | 0.98 |
| ENSG00000138698 | ENSG00000179761 | -0.95 |
| ENSG00000143819 | ENSG00000179761 | 0.95 |
| ENSG00000162407 | ENSG00000179761 | 0.95 |
| ENSG00000171853 | ENSG00000179761 | -0.95 |
| ENSG00000007402 | ENSG00000179841 | 0.96 |
| ENSG00000112234 | ENSG00000179899 | 0.95 |
| ENSG00000177504 | ENSG00000179965 | 0.97 |
| ENSG00000165509 | ENSG00000180104 | -0.97 |
| ENSG00000179271 | ENSG00000180182 | 0.95 |
| ENSG00000137936 | ENSG00000180198 | -0.95 |
| ENSG00000151287 | ENSG00000180198 | 0.94 |
| ENSG00000055957 | ENSG00000180210 | 0.95 |
| ENSG00000068796 | ENSG00000180210 | -0.97 |
| ENSG00000100024 | ENSG00000180210 | 0.96 |
| ENSG00000170458 | ENSG00000180210 | 0.95 |
| ENSG00000085063 | ENSG00000180304 | 0.95 |
| ENSG00000006116 | ENSG00000180318 | 0.97 |
| ENSG00000041515 | ENSG00000180318 | 0.94 |
| ENSG00000106123 | ENSG00000180318 | 0.94 |
| ENSG00000138653 | ENSG00000180318 | 0.95 |
| ENSG00000171551 | ENSG00000180318 | 0.97 |
| ENSG00000160007 | ENSG00000180353 | 0.95 |
| ENSG00000112110 | ENSG00000180530 | -0.95 |
| ENSG00000177189 | ENSG00000180543 | -0.97 |
| ENSG00000179583 | ENSG00000180644 | 0.96 |
| ENSG00000135723 | ENSG00000180739 | 0.98 |
| ENSG00000089234 | ENSG00000180855 | 0.95 |
| ENSG00000119965 | ENSG00000180855 | 0.95 |
| ENSG00000145826 | ENSG00000180855 | -0.95 |
| ENSG00000135773 | ENSG00000180891 | -0.95 |
| ENSG00000137100 | ENSG00000180901 | 0.97 |
| ENSG00000112699 | ENSG00000180957 | -0.95 |
| ENSG00000058600 | ENSG00000181191 | 0.95 |
| ENSG00000136908 | ENSG00000181191 | 0.95 |
| ENSG00000100918 | ENSG00000181222 | 0.95 |
| ENSG00000158714 | ENSG00000181274 | 0.97 |
| ENSG00000100348 | ENSG00000181588 | -0.95 |
| ENSG00000141425 | ENSG00000181751 | 0.96 |
| ENSG00000071539 | ENSG00000181784 | -0.95 |
| ENSG00000052126 | ENSG00000181830 | -0.95 |
| ENSG00000087053 | ENSG00000181852 | 0.95 |
| ENSG00000096872 | ENSG00000181852 | 0.96 |
| ENSG00000099812 | ENSG00000181885 | 0.96 |
| ENSG00000120756 | ENSG00000181885 | 0.95 |
| ENSG00000166145 | ENSG00000181885 | 0.96 |
| ENSG00000113387 | ENSG00000182004 | 0.95 |
| ENSG00000116337 | ENSG00000182013 | 0.95 |
| ENSG00000167191 | ENSG00000182022 | 0.95 |
| ENSG00000050426 | ENSG00000182054 | -0.94 |
| ENSG00000152804 | ENSG00000182093 | -0.96 |
| ENSG00000076554 | ENSG00000182107 | 0.95 |
| ENSG00000132437 | ENSG00000182107 | 0.96 |
| ENSG00000074695 | ENSG00000182195 | -0.96 |
| ENSG00000133169 | ENSG00000182195 | 0.97 |
| ENSG00000166922 | ENSG00000182195 | 0.96 |
| ENSG00000108799 | ENSG00000182220 | 0.96 |
| ENSG00000168903 | ENSG00000182253 | 0.95 |
| ENSG00000176092 | ENSG00000182253 | 0.96 |
| ENSG00000135517 | ENSG00000182324 | 0.94 |
| ENSG00000159403 | ENSG00000182326 | 0.98 |
| ENSG00000167987 | ENSG00000182326 | -0.94 |
| ENSG00000085788 | ENSG00000182457 | 0.94 |
| ENSG00000172845 | ENSG00000182457 | 0.95 |
| ENSG00000162433 | ENSG00000182606 | -0.95 |
| ENSG00000138433 | ENSG00000182611 | 0.94 |
| ENSG00000101079 | ENSG00000182631 | -0.95 |
| ENSG00000126254 | ENSG00000182674 | 0.94 |
| ENSG00000006607 | ENSG00000182768 | 0.94 |
| ENSG00000073584 | ENSG00000182768 | 0.94 |
| ENSG00000099956 | ENSG00000182768 | 0.95 |
| ENSG00000136813 | ENSG00000182768 | 0.95 |
| ENSG00000165030 | ENSG00000182768 | -0.95 |
| ENSG00000173862 | ENSG00000182768 | -0.95 |
| ENSG00000138041 | ENSG00000182774 | -0.96 |
| ENSG00000113083 | ENSG00000182791 | 0.95 |
| ENSG00000126838 | ENSG00000182871 | 0.95 |
| ENSG00000096093 | ENSG00000182872 | 0.97 |
| ENSG00000117153 | ENSG00000182872 | 0.95 |
| ENSG00000085465 | ENSG00000183044 | 0.95 |
| ENSG00000072163 | ENSG00000183049 | -0.95 |
| ENSG00000129158 | ENSG00000183049 | 0.95 |
| ENSG00000092850 | ENSG00000183150 | 0.97 |
| ENSG00000096654 | ENSG00000183207 | 0.95 |
| ENSG00000129351 | ENSG00000183207 | 0.95 |
| ENSG00000090661 | ENSG00000183230 | -0.95 |
| ENSG00000163106 | ENSG00000183230 | 0.95 |
| ENSG00000075415 | ENSG00000183337 | -0.95 |
| ENSG00000163875 | ENSG00000183337 | 0.95 |
| ENSG00000116539 | ENSG00000183434 | 0.94 |
| ENSG00000100575 | ENSG00000183473 | 0.95 |
| ENSG00000137200 | ENSG00000183486 | 0.96 |
| ENSG00000038358 | ENSG00000183549 | -0.95 |
| ENSG00000171853 | ENSG00000183549 | -0.94 |
| ENSG00000055732 | ENSG00000183576 | 0.95 |
| ENSG00000171735 | ENSG00000183576 | 0.95 |
| ENSG00000112667 | ENSG00000183629 | -0.94 |
| ENSG00000089737 | ENSG00000183718 | 0.94 |
| ENSG00000136315 | ENSG00000183718 | -0.94 |
| ENSG00000055118 | ENSG00000183741 | 0.96 |
| ENSG00000096093 | ENSG00000183741 | 0.95 |
| ENSG00000099326 | ENSG00000183741 | 0.95 |
| ENSG00000103024 | ENSG00000183741 | 0.95 |
| ENSG00000107281 | ENSG00000183741 | 0.95 |
| ENSG00000114956 | ENSG00000183741 | 0.95 |
| ENSG00000116128 | ENSG00000183741 | 0.98 |
| ENSG00000117791 | ENSG00000183741 | -0.95 |
| ENSG00000167535 | ENSG00000183741 | 0.94 |
| ENSG00000176894 | ENSG00000183741 | -0.95 |
| ENSG00000162645 | ENSG00000183963 | 0.96 |
| ENSG00000113361 | ENSG00000184047 | 0.95 |
| ENSG00000163218 | ENSG00000184056 | -0.95 |
| ENSG00000151552 | ENSG00000184117 | 0.95 |
| ENSG00000028310 | ENSG00000184247 | 0.96 |
| ENSG00000113790 | ENSG00000184247 | -0.95 |
| ENSG00000120837 | ENSG00000184314 | 0.95 |
| ENSG00000162777 | ENSG00000184363 | 0.95 |
| ENSG00000163694 | ENSG00000184363 | 0.94 |
| ENSG00000165215 | ENSG00000184363 | 0.97 |
| ENSG00000181885 | ENSG00000184363 | 0.95 |
| ENSG00000100170 | ENSG00000184434 | 0.95 |
| ENSG00000122121 | ENSG00000184434 | 0.95 |
| ENSG00000125255 | ENSG00000184434 | 0.96 |
| ENSG00000130234 | ENSG00000184434 | 0.96 |
| ENSG00000138079 | ENSG00000184434 | 0.95 |
| ENSG00000141434 | ENSG00000184434 | 0.96 |
| ENSG00000115966 | ENSG00000184545 | 0.95 |
| ENSG00000179915 | ENSG00000184545 | 0.96 |
| ENSG00000105993 | ENSG00000184566 | -0.95 |
| ENSG00000158477 | ENSG00000184566 | 0.96 |
| ENSG00000121417 | ENSG00000184634 | 0.95 |
| ENSG00000001617 | ENSG00000184674 | -0.95 |
| ENSG00000148572 | ENSG00000184677 | -0.94 |
| ENSG00000019505 | ENSG00000184678 | 0.95 |
| ENSG00000115020 | ENSG00000184678 | 0.96 |
| ENSG00000132359 | ENSG00000184678 | 0.96 |
| ENSG00000163497 | ENSG00000184678 | 0.95 |
| ENSG00000163499 | ENSG00000184678 | 0.97 |
| ENSG00000169862 | ENSG00000184678 | 0.96 |
| ENSG00000172575 | ENSG00000184678 | 0.95 |
| ENSG00000178695 | ENSG00000184678 | 0.94 |
| ENSG00000129187 | ENSG00000184867 | 0.94 |
| ENSG00000112320 | ENSG00000184897 | 0.95 |
| ENSG00000008056 | ENSG00000184905 | 0.95 |
| ENSG00000092096 | ENSG00000184905 | 0.98 |
| ENSG00000105270 | ENSG00000184905 | 0.95 |
| ENSG00000107758 | ENSG00000184905 | 0.97 |
| ENSG00000133169 | ENSG00000184905 | 0.95 |
| ENSG00000166922 | ENSG00000184905 | 0.95 |
| ENSG00000131981 | ENSG00000185000 | 0.94 |
| ENSG00000168765 | ENSG00000185000 | 0.97 |
| ENSG00000095794 | ENSG00000185010 | 0.96 |
| ENSG00000166145 | ENSG00000185010 | -0.95 |
| ENSG00000181885 | ENSG00000185010 | -0.95 |
| ENSG00000141696 | ENSG00000185024 | -0.97 |
| ENSG00000077984 | ENSG00000185044 | -0.95 |
| ENSG00000156709 | ENSG00000185049 | -0.97 |
| ENSG00000141279 | ENSG00000185052 | 0.94 |
| ENSG00000153885 | ENSG00000185069 | 0.95 |
| ENSG00000165509 | ENSG00000185069 | 0.97 |
| ENSG00000180104 | ENSG00000185069 | -0.95 |
| ENSG00000132376 | ENSG00000185088 | -0.97 |
| ENSG00000100842 | ENSG00000185187 | -0.96 |
| ENSG00000131781 | ENSG00000185187 | 0.96 |
| ENSG00000104413 | ENSG00000185201 | -0.96 |
| ENSG00000106541 | ENSG00000185201 | -0.95 |
| ENSG00000106993 | ENSG00000185201 | 0.96 |
| ENSG00000114529 | ENSG00000185201 | -0.96 |
| ENSG00000115226 | ENSG00000185201 | 0.97 |
| ENSG00000119888 | ENSG00000185201 | -0.96 |
| ENSG00000129354 | ENSG00000185201 | -0.98 |
| ENSG00000132698 | ENSG00000185201 | -0.97 |
| ENSG00000152990 | ENSG00000185201 | 0.95 |
| ENSG00000173801 | ENSG00000185201 | -0.95 |
| ENSG00000134160 | ENSG00000185313 | 0.94 |
| ENSG00000163531 | ENSG00000185324 | 0.96 |
| ENSG00000172348 | ENSG00000185324 | 0.95 |
| ENSG00000050405 | ENSG00000185347 | 0.95 |
| ENSG00000149428 | ENSG00000185359 | 0.94 |
| ENSG00000108010 | ENSG00000185404 | 0.95 |
| ENSG00000112655 | ENSG00000185420 | 0.95 |
| ENSG00000133318 | ENSG00000185420 | 0.95 |
| ENSG00000136451 | ENSG00000185420 | 0.95 |
| ENSG00000141568 | ENSG00000185420 | 0.95 |
| ENSG00000171766 | ENSG00000185420 | -0.95 |
| ENSG00000152284 | ENSG00000185432 | 0.95 |
| ENSG00000168291 | ENSG00000185432 | -0.95 |
| ENSG00000100288 | ENSG00000185513 | 0.96 |
| ENSG00000102978 | ENSG00000185513 | 0.95 |
| ENSG00000110811 | ENSG00000185513 | 0.95 |
| ENSG00000071189 | ENSG00000185527 | 0.95 |
| ENSG00000138735 | ENSG00000185608 | -0.95 |
| ENSG00000018610 | ENSG00000185721 | -0.95 |
| ENSG00000164244 | ENSG00000185721 | 0.95 |
| ENSG00000115221 | ENSG00000185839 | 0.96 |
| ENSG00000163131 | ENSG00000185839 | 0.95 |
| ENSG00000134809 | ENSG00000185885 | 0.95 |
| ENSG00000143842 | ENSG00000185885 | -0.96 |
| ENSG00000163932 | ENSG00000185885 | -0.95 |
| ENSG00000070476 | ENSG00000185894 | -0.95 |
| ENSG00000047249 | ENSG00000185928 | 0.95 |
| ENSG00000167965 | ENSG00000185928 | 0.95 |
| ENSG00000141441 | ENSG00000185986 | 0.96 |
| ENSG00000185483 | ENSG00000186001 | 0.96 |
| ENSG00000164919 | ENSG00000186010 | 0.95 |
| ENSG00000163159 | ENSG00000186115 | -0.95 |
| ENSG00000072786 | ENSG00000186141 | -0.98 |
| ENSG00000124935 | ENSG00000186141 | 0.96 |
| ENSG00000136352 | ENSG00000186141 | 0.95 |
| ENSG00000139352 | ENSG00000186141 | 0.95 |
| ENSG00000167332 | ENSG00000186141 | 0.97 |
| ENSG00000176049 | ENSG00000186153 | 0.95 |
| ENSG00000165678 | ENSG00000186204 | 0.95 |
| ENSG00000177082 | ENSG00000186204 | -0.95 |
| ENSG00000137513 | ENSG00000186205 | 0.94 |
| ENSG00000095261 | ENSG00000186260 | 0.94 |
| ENSG00000139323 | ENSG00000186298 | 0.96 |
| ENSG00000008056 | ENSG00000186310 | 0.96 |
| ENSG00000102409 | ENSG00000186310 | 0.96 |
| ENSG00000126947 | ENSG00000186310 | 0.96 |
| ENSG00000137817 | ENSG00000186310 | 0.95 |
| ENSG00000168917 | ENSG00000186318 | 0.95 |
| ENSG00000182326 | ENSG00000186350 | 0.96 |
| ENSG00000171928 | ENSG00000186442 | -0.95 |
| ENSG00000106089 | ENSG00000186462 | 0.95 |
| ENSG00000115020 | ENSG00000186462 | 0.95 |
| ENSG00000120251 | ENSG00000186462 | 0.96 |
| ENSG00000121671 | ENSG00000186462 | 0.97 |
| ENSG00000130643 | ENSG00000186462 | 0.96 |
| ENSG00000148798 | ENSG00000186462 | 0.96 |
| ENSG00000159409 | ENSG00000186462 | 0.95 |
| ENSG00000163497 | ENSG00000186462 | 0.97 |
| ENSG00000163531 | ENSG00000186462 | 0.94 |
| ENSG00000170233 | ENSG00000186462 | 0.95 |
| ENSG00000171476 | ENSG00000186462 | 0.95 |
| ENSG00000036565 | ENSG00000186487 | 0.96 |
| ENSG00000058404 | ENSG00000186487 | 0.95 |
| ENSG00000060709 | ENSG00000186487 | 0.94 |
| ENSG00000069020 | ENSG00000186487 | 0.94 |
| ENSG00000089199 | ENSG00000186487 | 0.95 |
| ENSG00000115020 | ENSG00000186487 | 0.97 |
| ENSG00000120251 | ENSG00000186487 | 0.98 |
| ENSG00000121671 | ENSG00000186487 | 0.96 |
| ENSG00000136960 | ENSG00000186487 | 0.96 |
| ENSG00000139970 | ENSG00000186487 | 0.98 |
| ENSG00000163497 | ENSG00000186487 | 0.95 |
| ENSG00000163499 | ENSG00000186487 | 0.97 |
| ENSG00000170745 | ENSG00000186487 | 0.95 |
| ENSG00000171476 | ENSG00000186487 | 0.95 |
| ENSG00000171793 | ENSG00000186487 | -0.95 |
| ENSG00000172348 | ENSG00000186487 | 0.95 |
| ENSG00000175866 | ENSG00000186487 | 0.97 |
| ENSG00000178695 | ENSG00000186487 | 0.98 |
| ENSG00000168461 | ENSG00000186642 | 0.96 |
| ENSG00000125730 | ENSG00000186715 | 0.94 |
| ENSG00000137409 | ENSG00000186715 | -0.94 |
| ENSG00000163581 | ENSG00000186716 | -0.96 |
| ENSG00000175575 | ENSG00000186732 | -0.95 |
| ENSG00000180198 | ENSG00000186842 | 0.97 |
| ENSG00000078618 | ENSG00000187097 | -0.95 |
| ENSG00000054356 | ENSG00000187164 | 0.96 |
| ENSG00000058404 | ENSG00000187164 | 0.97 |
| ENSG00000068971 | ENSG00000187164 | 0.95 |
| ENSG00000089199 | ENSG00000187164 | 0.95 |
| ENSG00000105290 | ENSG00000187164 | 0.95 |
| ENSG00000120251 | ENSG00000187164 | 0.96 |
| ENSG00000121671 | ENSG00000187164 | 0.95 |
| ENSG00000127252 | ENSG00000187164 | 0.96 |
| ENSG00000148123 | ENSG00000187164 | 0.95 |
| ENSG00000160460 | ENSG00000187164 | 0.95 |
| ENSG00000170233 | ENSG00000187164 | 0.95 |
| ENSG00000172478 | ENSG00000187164 | 0.94 |
| ENSG00000109472 | ENSG00000187189 | 0.96 |
| ENSG00000129990 | ENSG00000187189 | 0.96 |
| ENSG00000157388 | ENSG00000187189 | 0.95 |
| ENSG00000168280 | ENSG00000187189 | 0.95 |
| ENSG00000174996 | ENSG00000187189 | 0.95 |
| ENSG00000125144 | ENSG00000187193 | 0.97 |
| ENSG00000125148 | ENSG00000187193 | 0.99 |
| ENSG00000169715 | ENSG00000187193 | 0.97 |
| ENSG00000158560 | ENSG00000187266 | 0.95 |
| ENSG00000163875 | ENSG00000187266 | 0.95 |
| ENSG00000133121 | ENSG00000187323 | 0.95 |
| ENSG00000104611 | ENSG00000187391 | -0.98 |
| ENSG00000145194 | ENSG00000187522 | -0.95 |
| ENSG00000112818 | ENSG00000187534 | 0.95 |
| ENSG00000157064 | ENSG00000187601 | 0.95 |
| ENSG00000068078 | ENSG00000187608 | -0.95 |
| ENSG00000118804 | ENSG00000187608 | -0.95 |
| ENSG00000129084 | ENSG00000187608 | 0.94 |
| ENSG00000158050 | ENSG00000187714 | 0.98 |
| ENSG00000085365 | ENSG00000187735 | 0.96 |
| ENSG00000127445 | ENSG00000187742 | 0.94 |
| ENSG00000067057 | ENSG00000187764 | 0.95 |
| ENSG00000106804 | ENSG00000187764 | -0.97 |
| ENSG00000113141 | ENSG00000187764 | 0.95 |
| ENSG00000148300 | ENSG00000187764 | 0.96 |
| ENSG00000101445 | ENSG00000187801 | -0.95 |
| ENSG00000115386 | ENSG00000187908 | 0.95 |
| ENSG00000086589 | ENSG00000188021 | 0.97 |
| ENSG00000092098 | ENSG00000188021 | 0.95 |
| ENSG00000131238 | ENSG00000188021 | 0.95 |
| ENSG00000179222 | ENSG00000188070 | 0.94 |
| ENSG00000091664 | ENSG00000188295 | 0.95 |
| ENSG00000165912 | ENSG00000188419 | 0.95 |
| ENSG00000039537 | ENSG00000188488 | 0.96 |
| ENSG00000072080 | ENSG00000188488 | 0.95 |
| ENSG00000080910 | ENSG00000188488 | 0.95 |
| ENSG00000104760 | ENSG00000188488 | 0.97 |
| ENSG00000105697 | ENSG00000188488 | 0.95 |
| ENSG00000106927 | ENSG00000188488 | 0.96 |
| ENSG00000113905 | ENSG00000188488 | 0.97 |
| ENSG00000117601 | ENSG00000188488 | 0.95 |
| ENSG00000122194 | ENSG00000188488 | 0.94 |
| ENSG00000129988 | ENSG00000188488 | 0.96 |
| ENSG00000130649 | ENSG00000188488 | 0.95 |
| ENSG00000132693 | ENSG00000188488 | 0.96 |
| ENSG00000132703 | ENSG00000188488 | 0.97 |
| ENSG00000134365 | ENSG00000188488 | 0.98 |
| ENSG00000134389 | ENSG00000188488 | 0.95 |
| ENSG00000135094 | ENSG00000188488 | 0.98 |
| ENSG00000139547 | ENSG00000188488 | 0.96 |
| ENSG00000145826 | ENSG00000188488 | 0.96 |
| ENSG00000148702 | ENSG00000188488 | 0.95 |
| ENSG00000149124 | ENSG00000188488 | 0.95 |
| ENSG00000151655 | ENSG00000188488 | 0.98 |
| ENSG00000156096 | ENSG00000188488 | 0.95 |
| ENSG00000158104 | ENSG00000188488 | 0.95 |
| ENSG00000160801 | ENSG00000188488 | 0.97 |
| ENSG00000129845 | ENSG00000188763 | 0.96 |
| ENSG00000158477 | ENSG00000188763 | 0.95 |
| ENSG00000006116 | ENSG00000188778 | 0.95 |
| ENSG00000041515 | ENSG00000188778 | 0.95 |
| ENSG00000180318 | ENSG00000188778 | 0.95 |
| ENSG00000100385 | ENSG00000188822 | 0.96 |
| ENSG00000004487 | ENSG00000188986 | 0.95 |
| ENSG00000039537 | ENSG00000188986 | -0.96 |
| ENSG00000044115 | ENSG00000188986 | 0.96 |
| ENSG00000162517 | ENSG00000188986 | 0.94 |
| ENSG00000168994 | ENSG00000188986 | -0.96 |
| ENSG00000071243 | ENSG00000188994 | 0.95 |
| ENSG00000134463 | ENSG00000189002 | 0.95 |
| ENSG00000080546 | ENSG00000189043 | -0.95 |
| ENSG00000115020 | ENSG00000189043 | -0.95 |
| ENSG00000163499 | ENSG00000189043 | -0.95 |
| ENSG00000169862 | ENSG00000189043 | -0.96 |
| ENSG00000107185 | ENSG00000189056 | -0.95 |
| ENSG00000095397 | ENSG00000189091 | 0.96 |
| ENSG00000175182 | ENSG00000189091 | 0.97 |
| ENSG00000100393 | ENSG00000189093 | 0.95 |
| ENSG00000101333 | ENSG00000189093 | 0.95 |
| ENSG00000064989 | ENSG00000189108 | 0.96 |
| ENSG00000114529 | ENSG00000189143 | 0.98 |
| ENSG00000129354 | ENSG00000189143 | 0.95 |
| ENSG00000132698 | ENSG00000189143 | 0.95 |
| ENSG00000173801 | ENSG00000189143 | 0.95 |
| ENSG00000122359 | ENSG00000189159 | 0.95 |
| ENSG00000145192 | ENSG00000189159 | -0.94 |
| ENSG00000180957 | ENSG00000189159 | -0.95 |
| ENSG00000124535 | ENSG00000189162 | 0.95 |
| ENSG00000130234 | ENSG00000189221 | 0.95 |
| ENSG00000141434 | ENSG00000189221 | 0.95 |
| ENSG00000155465 | ENSG00000189221 | 0.94 |
| ENSG00000181396 | ENSG00000189221 | -0.96 |
| ENSG00000184434 | ENSG00000189221 | 0.95 |
| ENSG00000140718 | ENSG00000189266 | -0.96 |
| ENSG00000112164 | ENSG00000189283 | 0.95 |
| ENSG00000121380 | ENSG00000189334 | 0.95 |
| ENSG00000134028 | ENSG00000189334 | 0.95 |
| ENSG00000101079 | ENSG00000189339 | 0.95 |
| ENSG00000117069 | ENSG00000196090 | 0.96 |
| ENSG00000117090 | ENSG00000196090 | -0.95 |
| ENSG00000136040 | ENSG00000196090 | 0.97 |
| ENSG00000151208 | ENSG00000196090 | 0.97 |
| ENSG00000176105 | ENSG00000196090 | -0.95 |
| ENSG00000163810 | ENSG00000196091 | 0.96 |
| ENSG00000184566 | ENSG00000196115 | 0.95 |
| ENSG00000179958 | ENSG00000196116 | 0.95 |
| ENSG00000029534 | ENSG00000196132 | 0.95 |
| ENSG00000075240 | ENSG00000196136 | 0.97 |
| ENSG00000111181 | ENSG00000196136 | 0.96 |
| ENSG00000113889 | ENSG00000196136 | 0.95 |
| ENSG00000132693 | ENSG00000196136 | 0.96 |
| ENSG00000151655 | ENSG00000196136 | 0.95 |
| ENSG00000151790 | ENSG00000196136 | 0.96 |
| ENSG00000158874 | ENSG00000196136 | 0.98 |
| ENSG00000071575 | ENSG00000196139 | -0.96 |
| ENSG00000122687 | ENSG00000196139 | -0.96 |
| ENSG00000123240 | ENSG00000196154 | 0.95 |
| ENSG00000171453 | ENSG00000196154 | -0.94 |
| ENSG00000115364 | ENSG00000196208 | -0.95 |
| ENSG00000029534 | ENSG00000196220 | 0.96 |
| ENSG00000058404 | ENSG00000196220 | 0.94 |
| ENSG00000103723 | ENSG00000196220 | 0.94 |
| ENSG00000130540 | ENSG00000196220 | 0.95 |
| ENSG00000160460 | ENSG00000196220 | 0.97 |
| ENSG00000196132 | ENSG00000196220 | 0.95 |
| ENSG00000106803 | ENSG00000196226 | 0.95 |
| ENSG00000182289 | ENSG00000196338 | 0.94 |
| ENSG00000111110 | ENSG00000196345 | 0.95 |
| ENSG00000133624 | ENSG00000196345 | 0.95 |
| ENSG00000179899 | ENSG00000196345 | 0.96 |
| ENSG00000068078 | ENSG00000196372 | 0.97 |
| ENSG00000117069 | ENSG00000196405 | 0.95 |
| ENSG00000128918 | ENSG00000196405 | 0.95 |
| ENSG00000135678 | ENSG00000196419 | -0.97 |
| ENSG00000151883 | ENSG00000196455 | 0.96 |
| ENSG00000068745 | ENSG00000196465 | 0.97 |
| ENSG00000074621 | ENSG00000196465 | 0.95 |
| ENSG00000134716 | ENSG00000196502 | 0.95 |
| ENSG00000138030 | ENSG00000196502 | 0.96 |
| ENSG00000138823 | ENSG00000196502 | 0.96 |
| ENSG00000172831 | ENSG00000196502 | 0.97 |
| ENSG00000158270 | ENSG00000196511 | 0.94 |
| ENSG00000179604 | ENSG00000196544 | 0.95 |
| ENSG00000183576 | ENSG00000196544 | 0.95 |
| ENSG00000068971 | ENSG00000196547 | 0.94 |
| ENSG00000168291 | ENSG00000196565 | -0.97 |
| ENSG00000102054 | ENSG00000196591 | 0.95 |
| ENSG00000119777 | ENSG00000196611 | 0.96 |
| ENSG00000135924 | ENSG00000196655 | 0.97 |
| ENSG00000156030 | ENSG00000196655 | 0.95 |
| ENSG00000171130 | ENSG00000196663 | 0.95 |
| ENSG00000170222 | ENSG00000196743 | 0.95 |
| ENSG00000047188 | ENSG00000196754 | 0.96 |
| ENSG00000134905 | ENSG00000196812 | 0.96 |
| ENSG00000136828 | ENSG00000196812 | 0.95 |
| ENSG00000162551 | ENSG00000196812 | -0.95 |
| ENSG00000174808 | ENSG00000196812 | 0.96 |
| ENSG00000122121 | ENSG00000196878 | 0.95 |
| ENSG00000065618 | ENSG00000196975 | 0.95 |
| ENSG00000074276 | ENSG00000196975 | 0.94 |
| ENSG00000145384 | ENSG00000196975 | 0.96 |
| ENSG00000072121 | ENSG00000197102 | 0.95 |
| ENSG00000100292 | ENSG00000197102 | -0.95 |
| ENSG00000100385 | ENSG00000197102 | -0.95 |
| ENSG00000101191 | ENSG00000197102 | 0.96 |
| ENSG00000116675 | ENSG00000197102 | 0.94 |
| ENSG00000167965 | ENSG00000197102 | 0.96 |
| ENSG00000114054 | ENSG00000197114 | 0.95 |
| ENSG00000145936 | ENSG00000197142 | 0.98 |
| ENSG00000129028 | ENSG00000197165 | -0.95 |
| ENSG00000134716 | ENSG00000197165 | 0.95 |
| ENSG00000138030 | ENSG00000197165 | 0.96 |
| ENSG00000138823 | ENSG00000197165 | 0.96 |
| ENSG00000172831 | ENSG00000197165 | 0.96 |
| ENSG00000196502 | ENSG00000197165 | 0.99 |
| ENSG00000133135 | ENSG00000197249 | 0.95 |
| ENSG00000146242 | ENSG00000197249 | -0.97 |
| ENSG00000040199 | ENSG00000197273 | 0.95 |
| ENSG00000080031 | ENSG00000197273 | 0.95 |
| ENSG00000108272 | ENSG00000197273 | 0.97 |
| ENSG00000114455 | ENSG00000197273 | 0.97 |
| ENSG00000130234 | ENSG00000197273 | 0.95 |
| ENSG00000155465 | ENSG00000197273 | 0.96 |
| ENSG00000189221 | ENSG00000197273 | 0.96 |
| ENSG00000162613 | ENSG00000197275 | 0.95 |
| ENSG00000083454 | ENSG00000197323 | -0.95 |
| ENSG00000176454 | ENSG00000197324 | 0.95 |
| ENSG00000135373 | ENSG00000197358 | -0.95 |
| ENSG00000158158 | ENSG00000197375 | 0.96 |
| ENSG00000163430 | ENSG00000197381 | 0.95 |
| ENSG00000106524 | ENSG00000197386 | 0.96 |
| ENSG00000107537 | ENSG00000197408 | 0.96 |
| ENSG00000171603 | ENSG00000197408 | -0.96 |
| ENSG00000102038 | ENSG00000197444 | 0.95 |
| ENSG00000034713 | ENSG00000197535 | 0.96 |
| ENSG00000165801 | ENSG00000197535 | 0.96 |
| ENSG00000172348 | ENSG00000197535 | 0.96 |
| ENSG00000175866 | ENSG00000197535 | 0.97 |
| ENSG00000185324 | ENSG00000197535 | 0.95 |
| ENSG00000125970 | ENSG00000197563 | 0.96 |
| ENSG00000135744 | ENSG00000197563 | -0.95 |
| ENSG00000075340 | ENSG00000197586 | -0.94 |
| ENSG00000124713 | ENSG00000197586 | -0.95 |
| ENSG00000172283 | ENSG00000197586 | -0.94 |
| ENSG00000107745 | ENSG00000197619 | -0.95 |
| ENSG00000117013 | ENSG00000197622 | 0.97 |
| ENSG00000022267 | ENSG00000197635 | 0.96 |
| ENSG00000070501 | ENSG00000197635 | -0.96 |
| ENSG00000099834 | ENSG00000197635 | 0.95 |
| ENSG00000067836 | ENSG00000197694 | 0.96 |
| ENSG00000069764 | ENSG00000197694 | 0.97 |
| ENSG00000100505 | ENSG00000197694 | 0.96 |
| ENSG00000106976 | ENSG00000197694 | 0.96 |
| ENSG00000107077 | ENSG00000197694 | 0.95 |
| ENSG00000135924 | ENSG00000197694 | 0.95 |
| ENSG00000140750 | ENSG00000197694 | 0.95 |
| ENSG00000156030 | ENSG00000197694 | 0.95 |
| ENSG00000163214 | ENSG00000197694 | 0.97 |
| ENSG00000173692 | ENSG00000197694 | 0.95 |
| ENSG00000110887 | ENSG00000197711 | 0.95 |
| ENSG00000135094 | ENSG00000197711 | 0.96 |
| ENSG00000136238 | ENSG00000197711 | -0.95 |
| ENSG00000148702 | ENSG00000197711 | 0.95 |
| ENSG00000160801 | ENSG00000197711 | 0.95 |
| ENSG00000163631 | ENSG00000197711 | 0.95 |
| ENSG00000188488 | ENSG00000197711 | 0.95 |
| ENSG00000106799 | ENSG00000197713 | 0.95 |
| ENSG00000157036 | ENSG00000197724 | 0.97 |
| ENSG00000162367 | ENSG00000197724 | -0.95 |
| ENSG00000168280 | ENSG00000197724 | 0.95 |
| ENSG00000174996 | ENSG00000197724 | 0.95 |
| ENSG00000137757 | ENSG00000197747 | 0.96 |
| ENSG00000171431 | ENSG00000197747 | 0.94 |
| ENSG00000072121 | ENSG00000197818 | 0.95 |
| ENSG00000101452 | ENSG00000197818 | 0.95 |
| ENSG00000143409 | ENSG00000197818 | 0.95 |
| ENSG00000155868 | ENSG00000197818 | 0.95 |
| ENSG00000161010 | ENSG00000197818 | 0.95 |
| ENSG00000154040 | ENSG00000197830 | 0.94 |
| ENSG00000099869 | ENSG00000197838 | 0.95 |
| ENSG00000116984 | ENSG00000197846 | -0.97 |
| ENSG00000170962 | ENSG00000197846 | -0.95 |
| ENSG00000112110 | ENSG00000197858 | 0.96 |
| ENSG00000186625 | ENSG00000197858 | 0.95 |
| ENSG00000006116 | ENSG00000197872 | 0.97 |
| ENSG00000073350 | ENSG00000197872 | -0.97 |
| ENSG00000119537 | ENSG00000197872 | 0.98 |
| ENSG00000135525 | ENSG00000197872 | -0.96 |
| ENSG00000177106 | ENSG00000197872 | -0.95 |
| ENSG00000003147 | ENSG00000197892 | 0.96 |
| ENSG00000072135 | ENSG00000197892 | 0.96 |
| ENSG00000143842 | ENSG00000197892 | 0.97 |
| ENSG00000168772 | ENSG00000197892 | 0.97 |
| ENSG00000111199 | ENSG00000197901 | 0.97 |
| ENSG00000108852 | ENSG00000197912 | 0.95 |
| ENSG00000118160 | ENSG00000197912 | 0.96 |
| ENSG00000128918 | ENSG00000197912 | 0.94 |
| ENSG00000150625 | ENSG00000197912 | 0.95 |
| ENSG00000158445 | ENSG00000197912 | 0.95 |
| ENSG00000166405 | ENSG00000197912 | 0.97 |
| ENSG00000166780 | ENSG00000197912 | 0.95 |
| ENSG00000196405 | ENSG00000197912 | 0.95 |
| ENSG00000006659 | ENSG00000197919 | 0.95 |
| ENSG00000172830 | ENSG00000197956 | 0.95 |
| ENSG00000175793 | ENSG00000197993 | 0.95 |
| ENSG00000060762 | ENSG00000198000 | -0.95 |
| ENSG00000138823 | ENSG00000198018 | 0.95 |
| ENSG00000066336 | ENSG00000198056 | -0.95 |
| ENSG00000196975 | ENSG00000198074 | 0.95 |
| ENSG00000079557 | ENSG00000198077 | 0.95 |
| ENSG00000080910 | ENSG00000198077 | 0.99 |
| ENSG00000099937 | ENSG00000198077 | 0.95 |
| ENSG00000100652 | ENSG00000198077 | 0.95 |
| ENSG00000104760 | ENSG00000198077 | 0.94 |
| ENSG00000104938 | ENSG00000198077 | 0.95 |
| ENSG00000105697 | ENSG00000198077 | 0.95 |
| ENSG00000106927 | ENSG00000198077 | 0.94 |
| ENSG00000109758 | ENSG00000198077 | 0.97 |
| ENSG00000110169 | ENSG00000198077 | 0.99 |
| ENSG00000111181 | ENSG00000198077 | 0.96 |
| ENSG00000113905 | ENSG00000198077 | 0.96 |
| ENSG00000117594 | ENSG00000198077 | 0.98 |
| ENSG00000117601 | ENSG00000198077 | 0.96 |
| ENSG00000122194 | ENSG00000198077 | 0.95 |
| ENSG00000126759 | ENSG00000198077 | 0.96 |
| ENSG00000129988 | ENSG00000198077 | 0.98 |
| ENSG00000134389 | ENSG00000198077 | 0.95 |
| ENSG00000136881 | ENSG00000198077 | 0.95 |
| ENSG00000139547 | ENSG00000198077 | 0.96 |
| ENSG00000140505 | ENSG00000198077 | 0.97 |
| ENSG00000149124 | ENSG00000198077 | 0.96 |
| ENSG00000151632 | ENSG00000198077 | 0.94 |
| ENSG00000158104 | ENSG00000198077 | 0.96 |
| ENSG00000162365 | ENSG00000198077 | 0.95 |
| ENSG00000166035 | ENSG00000198077 | 0.95 |
| ENSG00000172482 | ENSG00000198077 | 0.95 |
| ENSG00000155962 | ENSG00000198087 | 0.95 |
| ENSG00000165475 | ENSG00000198113 | 0.94 |
| ENSG00000187514 | ENSG00000198134 | 0.97 |
| ENSG00000166579 | ENSG00000198176 | 0.95 |
| ENSG00000136193 | ENSG00000198218 | 0.96 |
| ENSG00000104343 | ENSG00000198301 | 0.95 |
| ENSG00000116151 | ENSG00000198353 | -0.97 |
| ENSG00000132781 | ENSG00000198353 | 0.95 |
| ENSG00000166887 | ENSG00000198366 | -0.95 |
| ENSG00000148824 | ENSG00000198369 | 0.95 |
| ENSG00000100266 | ENSG00000198373 | 0.95 |
| ENSG00000168497 | ENSG00000198417 | 0.95 |
| ENSG00000169715 | ENSG00000198417 | 0.95 |
| ENSG00000124827 | ENSG00000198477 | 0.97 |
| ENSG00000127463 | ENSG00000198515 | -0.95 |
| ENSG00000156858 | ENSG00000198515 | -0.96 |
| ENSG00000089472 | ENSG00000198523 | 0.98 |
| ENSG00000163431 | ENSG00000198523 | 0.94 |
| ENSG00000178623 | ENSG00000198523 | 0.96 |
| ENSG00000163288 | ENSG00000198576 | 0.95 |
| ENSG00000171885 | ENSG00000198576 | 0.94 |
| ENSG00000079102 | ENSG00000198589 | 0.95 |
| ENSG00000116675 | ENSG00000198589 | 0.95 |
| ENSG00000166401 | ENSG00000198589 | -0.95 |
| ENSG00000102554 | ENSG00000198598 | -0.96 |
| ENSG00000135506 | ENSG00000198604 | -0.95 |
| ENSG00000138079 | ENSG00000198624 | 0.95 |
| ENSG00000004487 | ENSG00000198646 | 0.95 |
| ENSG00000116016 | ENSG00000198646 | -0.95 |
| ENSG00000128656 | ENSG00000198646 | 0.96 |
| ENSG00000135457 | ENSG00000198646 | 0.96 |
| ENSG00000137776 | ENSG00000198646 | 0.95 |
| ENSG00000149179 | ENSG00000198646 | 0.95 |
| ENSG00000171603 | ENSG00000198646 | 0.96 |
| ENSG00000171824 | ENSG00000198646 | 0.95 |
| ENSG00000197102 | ENSG00000198646 | 0.96 |
| ENSG00000122194 | ENSG00000198650 | 0.97 |
| ENSG00000138698 | ENSG00000198668 | 0.95 |
| ENSG00000019169 | ENSG00000198671 | 0.95 |
| ENSG00000039537 | ENSG00000198671 | 0.96 |
| ENSG00000072121 | ENSG00000198671 | -0.95 |
| ENSG00000100385 | ENSG00000198671 | 0.94 |
| ENSG00000101191 | ENSG00000198671 | -0.96 |
| ENSG00000128731 | ENSG00000198671 | -0.94 |
| ENSG00000134365 | ENSG00000198671 | 0.95 |
| ENSG00000145826 | ENSG00000198671 | 0.95 |
| ENSG00000155868 | ENSG00000198671 | -0.95 |
| ENSG00000168994 | ENSG00000198671 | 0.98 |
| ENSG00000171824 | ENSG00000198671 | -0.96 |
| ENSG00000188986 | ENSG00000198671 | -0.96 |
| ENSG00000197102 | ENSG00000198671 | -0.96 |
| ENSG00000122304 | ENSG00000198685 | 0.95 |
| ENSG00000012817 | ENSG00000198692 | 0.97 |
| ENSG00000067048 | ENSG00000198692 | 0.97 |
| ENSG00000114374 | ENSG00000198692 | 0.96 |
| ENSG00000129824 | ENSG00000198692 | 0.97 |
| ENSG00000101004 | ENSG00000198718 | 0.95 |
| ENSG00000117448 | ENSG00000198718 | -0.96 |
| ENSG00000148082 | ENSG00000198728 | 0.94 |
| ENSG00000171885 | ENSG00000198728 | 0.95 |
| ENSG00000118271 | ENSG00000198734 | 0.97 |
| ENSG00000138613 | ENSG00000198734 | 0.95 |
| ENSG00000147647 | ENSG00000198736 | 0.95 |
| ENSG00000006611 | ENSG00000198758 | 0.97 |
| ENSG00000079112 | ENSG00000198758 | 0.96 |
| ENSG00000127831 | ENSG00000198758 | 0.94 |
| ENSG00000165556 | ENSG00000198758 | 0.98 |
| ENSG00000106819 | ENSG00000198765 | 0.97 |
| ENSG00000143167 | ENSG00000198788 | 0.96 |
| ENSG00000019505 | ENSG00000198794 | 0.96 |
| ENSG00000123201 | ENSG00000198797 | 0.95 |
| ENSG00000128683 | ENSG00000198797 | 0.96 |
| ENSG00000133135 | ENSG00000198797 | -0.99 |
| ENSG00000135363 | ENSG00000198797 | 0.95 |
| ENSG00000165973 | ENSG00000198797 | 0.95 |
| ENSG00000115221 | ENSG00000198805 | 0.95 |
| ENSG00000196700 | ENSG00000198805 | -0.95 |
| ENSG00000182621 | ENSG00000198826 | 0.95 |
| ENSG00000171155 | ENSG00000198855 | -0.96 |
| ENSG00000134291 | ENSG00000198898 | 0.95 |
| ENSG00000087586 | ENSG00000198901 | 0.96 |
| ENSG00000101003 | ENSG00000198901 | 0.95 |
| ENSG00000179967 | ENSG00000198901 | 0.95 |
| ENSG00000119723 | ENSG00000198908 | 0.95 |
| ENSG00000130844 | ENSG00000198908 | 0.95 |
| ENSG00000132639 | ENSG00000198919 | 0.98 |
| ENSG00000137193 | ENSG00000198919 | -0.95 |
| ENSG00000170233 | ENSG00000198919 | 0.95 |
| ENSG00000076650 | ENSG00000198920 | 0.95 |
| ENSG00000008056 | ENSG00000198932 | 0.96 |
| ENSG00000089199 | ENSG00000198932 | 0.95 |
| ENSG00000104723 | ENSG00000198932 | 0.96 |
| ENSG00000106089 | ENSG00000198932 | 0.95 |
| ENSG00000120645 | ENSG00000198932 | 0.95 |
| ENSG00000121671 | ENSG00000198932 | 0.96 |
| ENSG00000132639 | ENSG00000198932 | 0.95 |
| ENSG00000133169 | ENSG00000198932 | 0.96 |
| ENSG00000135709 | ENSG00000198932 | 0.95 |
| ENSG00000148798 | ENSG00000198932 | 0.98 |
| ENSG00000166922 | ENSG00000198932 | 0.96 |
| ENSG00000184905 | ENSG00000198932 | 0.95 |
| ENSG00000186462 | ENSG00000198932 | 0.96 |
| ENSG00000187164 | ENSG00000198932 | 0.96 |
| ENSG00000157933 | ENSG00000198947 | 0.95 |
| ENSG00000160967 | ENSG00000198948 | -0.96 |
| ENSG00000032389 | ENSG00000198952 | 0.97 |
| ENSG00000145868 | ENSG00000198952 | 0.96 |
| ENSG00000147536 | ENSG00000198952 | -0.94 |
| ENSG00000184144 | ENSG00000198952 | -0.94 |
| ENSG00000198610 | ENSG00000198959 | 0.95 |
| ENSG00000171873 | ENSG00000198963 | 0.96 |
| ENSG00000163586 | ENSG00000200769 | -0.95 |
| ENSG00000100614 | ENSG00000200983 | -0.95 |
| ENSG00000109381 | ENSG00000202252 | -0.96 |
| ENSG00000065609 | ENSG00000203618 | 0.95 |
| ENSG00000171368 | ENSG00000203797 | 0.95 |
| ENSG00000023839 | ENSG00000203825 | -0.95 |
| ENSG00000072832 | ENSG00000203825 | 0.95 |
| ENSG00000103723 | ENSG00000203825 | 0.94 |
| ENSG00000105290 | ENSG00000203825 | 0.98 |
| ENSG00000107281 | ENSG00000203825 | 0.95 |
| ENSG00000108309 | ENSG00000203825 | 0.97 |
| ENSG00000109472 | ENSG00000203825 | 0.94 |
| ENSG00000112234 | ENSG00000203825 | 0.95 |
| ENSG00000132639 | ENSG00000203825 | 0.94 |
| ENSG00000136854 | ENSG00000203825 | 0.96 |
| ENSG00000137193 | ENSG00000203825 | -0.94 |
| ENSG00000140939 | ENSG00000203825 | 0.95 |
| ENSG00000145730 | ENSG00000203825 | 0.95 |
| ENSG00000160460 | ENSG00000203825 | 0.95 |
| ENSG00000174938 | ENSG00000203825 | 0.95 |
| ENSG00000196345 | ENSG00000203825 | 0.95 |
| ENSG00000163938 | ENSG00000203827 | 0.94 |
| ENSG00000145113 | ENSG00000203876 | -0.96 |
| ENSG00000072071 | ENSG00000203879 | 0.96 |
| ENSG00000108309 | ENSG00000203879 | 0.95 |
| ENSG00000109472 | ENSG00000203879 | 0.96 |
| ENSG00000137193 | ENSG00000203879 | -0.94 |
| ENSG00000140939 | ENSG00000203879 | 0.96 |
| ENSG00000145730 | ENSG00000203879 | 0.95 |
| ENSG00000160460 | ENSG00000203879 | 0.95 |
| ENSG00000167972 | ENSG00000203879 | 0.95 |
| ENSG00000176894 | ENSG00000203879 | -0.95 |
| ENSG00000102316 | ENSG00000203880 | 0.94 |
| ENSG00000178146 | ENSG00000203880 | -0.95 |
| ENSG00000137076 | ENSG00000203883 | 0.95 |
| ENSG00000107104 | ENSG00000203950 | -0.95 |
| ENSG00000072071 | ENSG00000204072 | 0.94 |
| ENSG00000163629 | ENSG00000204072 | 0.96 |
| ENSG00000005471 | ENSG00000204128 | 0.96 |
| ENSG00000100288 | ENSG00000204262 | 0.96 |
| ENSG00000185513 | ENSG00000204262 | 0.96 |
| ENSG00000079112 | ENSG00000204325 | 0.95 |
| ENSG00000007312 | ENSG00000204370 | 0.96 |
| ENSG00000185565 | ENSG00000204375 | 0.97 |
| ENSG00000086189 | ENSG00000204434 | 0.95 |
| ENSG00000183690 | ENSG00000204505 | 0.96 |
| ENSG00000100299 | ENSG00000204519 | 0.95 |
| ENSG00000028310 | ENSG00000204580 | 0.96 |
| ENSG00000085511 | ENSG00000204604 | 0.96 |
| ENSG00000111679 | ENSG00000204604 | 0.95 |
| ENSG00000167578 | ENSG00000204604 | 0.95 |
| ENSG00000111653 | ENSG00000204689 | -0.96 |
| ENSG00000185215 | ENSG00000204689 | 0.97 |
| ENSG00000111319 | ENSG00000204852 | -0.96 |
| ENSG00000141655 | ENSG00000204852 | -0.95 |
| ENSG00000114473 | ENSG00000204856 | 0.96 |
| ENSG00000136854 | ENSG00000204859 | 0.95 |
| ENSG00000167967 | ENSG00000204859 | 0.96 |
| ENSG00000081059 | ENSG00000204872 | 0.95 |
| ENSG00000122718 | ENSG00000204941 | 0.96 |
| ENSG00000153012 | ENSG00000204941 | 0.96 |
| ENSG00000144852 | ENSG00000204946 | -0.95 |
| ENSG00000186115 | ENSG00000204946 | -0.95 |
| ENSG00000138069 | ENSG00000205189 | -0.95 |
| ENSG00000178105 | ENSG00000205189 | 0.95 |
| ENSG00000128606 | ENSG00000205220 | -0.95 |
| ENSG00000132182 | ENSG00000205307 | 0.95 |
| ENSG00000125144 | ENSG00000205358 | 0.97 |
| ENSG00000125148 | ENSG00000205358 | 0.98 |
| ENSG00000164930 | ENSG00000205358 | -0.95 |
| ENSG00000169715 | ENSG00000205358 | 0.96 |
| ENSG00000187193 | ENSG00000205358 | 0.98 |
| ENSG00000148672 | ENSG00000205364 | 0.97 |
| ENSG00000163625 | ENSG00000205560 | 0.96 |
| ENSG00000167286 | ENSG00000205560 | -0.95 |
| ENSG00000105063 | ENSG00000205575 | 0.97 |
| ENSG00000168476 | ENSG00000205575 | 0.95 |
| ENSG00000100003 | ENSG00000205629 | -0.95 |
| ENSG00000148834 | ENSG00000205629 | -0.96 |
| ENSG00000139352 | ENSG00000205631 | 0.96 |
| ENSG00000164654 | ENSG00000205631 | 0.95 |
| ENSG00000100813 | ENSG00000205702 | -0.95 |
| ENSG00000167994 | ENSG00000205771 | -0.95 |
| ENSG00000122711 | ENSG00000206075 | 0.95 |
| ENSG00000168291 | ENSG00000206172 | -0.95 |
| ENSG00000183479 | ENSG00000206178 | 0.94 |
| ENSG00000103657 | ENSG00000206466 | 0.95 |
| ENSG00000178773 | ENSG00000206471 | 0.96 |
| ENSG00000102109 | ENSG00000206530 | 0.95 |
| ENSG00000132359 | ENSG00000206530 | 0.95 |
| ENSG00000176095 | ENSG00000206530 | 0.95 |
| ENSG00000184678 | ENSG00000206530 | 0.95 |
| ENSG00000173077 | ENSG00000206561 | 0.95 |
| ENSG00000137080 | ENSG00000206799 | 0.95 |
| ENSG00000136950 | ENSG00000207367 | 0.95 |
| ENSG00000070756 | ENSG00000207496 | 0.95 |
| ENSG00000105372 | ENSG00000207496 | 0.95 |
| ENSG00000137154 | ENSG00000207496 | 0.98 |
| ENSG00000129270 | ENSG00000207740 | -0.96 |
| ENSG00000184492 | ENSG00000207740 | 0.95 |
| ENSG00000004468 | ENSG00000207836 | 0.96 |
| ENSG00000168496 | ENSG00000208036 | 0.95 |
| ENSG00000005471 | ENSG00000211445 | 0.95 |
| ENSG00000107263 | ENSG00000211445 | 0.96 |
| ENSG00000204128 | ENSG00000211445 | 0.95 |
| ENSG00000172456 | ENSG00000211455 | -0.95 |
| ENSG00000132465 | ENSG00000211592 | 0.97 |
| ENSG00000165757 | ENSG00000211592 | -0.95 |
| ENSG00000172869 | ENSG00000211598 | -0.95 |
| ENSG00000206066 | ENSG00000211598 | 0.96 |
| ENSG00000206066 | ENSG00000211630 | 0.95 |
| ENSG00000207836 | ENSG00000211630 | 0.94 |
| ENSG00000129009 | ENSG00000211659 | -0.96 |
| ENSG00000129038 | ENSG00000211659 | -0.95 |
| ENSG00000158714 | ENSG00000211659 | 0.95 |
| ENSG00000181274 | ENSG00000211659 | 0.96 |
| ENSG00000170476 | ENSG00000211669 | 0.98 |
| ENSG00000172869 | ENSG00000211679 | -0.96 |
| ENSG00000207836 | ENSG00000211679 | 0.94 |
| ENSG00000211592 | ENSG00000211679 | 0.95 |
| ENSG00000211598 | ENSG00000211679 | 0.96 |
| ENSG00000211630 | ENSG00000211679 | 0.95 |
| ENSG00000161570 | ENSG00000211772 | 0.95 |
| ENSG00000141506 | ENSG00000211796 | 0.95 |
| ENSG00000163935 | ENSG00000211809 | -0.95 |
| ENSG00000104371 | ENSG00000211829 | 0.95 |
| ENSG00000138653 | ENSG00000211829 | 0.94 |
| ENSG00000197959 | ENSG00000211889 | -0.97 |
| ENSG00000132465 | ENSG00000211895 | 0.97 |
| ENSG00000211592 | ENSG00000211896 | 0.95 |
| ENSG00000211659 | ENSG00000211896 | 0.95 |
| ENSG00000211679 | ENSG00000211896 | 0.95 |
| ENSG00000165792 | ENSG00000211898 | -0.95 |
| ENSG00000040341 | ENSG00000211899 | -0.96 |
| ENSG00000206066 | ENSG00000211934 | 0.95 |
| ENSG00000211630 | ENSG00000211934 | 0.97 |
| ENSG00000206066 | ENSG00000211940 | 0.97 |
| ENSG00000207836 | ENSG00000211940 | 0.96 |
| ENSG00000211598 | ENSG00000211940 | 0.95 |
| ENSG00000211663 | ENSG00000211946 | 0.94 |
| ENSG00000004468 | ENSG00000211947 | 0.96 |
| ENSG00000169813 | ENSG00000211949 | 0.97 |
| ENSG00000211934 | ENSG00000211955 | 0.95 |
| ENSG00000211949 | ENSG00000211955 | 0.95 |
| ENSG00000211598 | ENSG00000211970 | 0.94 |
| ENSG00000211938 | ENSG00000211970 | 0.98 |
| ENSG00000206066 | ENSG00000211973 | 0.95 |
| ENSG00000211940 | ENSG00000211973 | 0.95 |
| ENSG00000152904 | ENSG00000212670 | 0.95 |
| ENSG00000055957 | ENSG00000212738 | -0.95 |
| ENSG00000156711 | ENSG00000212738 | 0.95 |
| ENSG00000095752 | ENSG00000212998 | 0.96 |
| ENSG00000132840 | ENSG00000213015 | -0.95 |
| ENSG00000163374 | ENSG00000213024 | 0.95 |
| ENSG00000167968 | ENSG00000213063 | -0.95 |
| ENSG00000100324 | ENSG00000213246 | 0.97 |
| ENSG00000150361 | ENSG00000213297 | -0.94 |
| ENSG00000066813 | ENSG00000213346 | -0.97 |
| ENSG00000102967 | ENSG00000213346 | -0.96 |
| ENSG00000106804 | ENSG00000213346 | -0.95 |
| ENSG00000109572 | ENSG00000213346 | 0.95 |
| ENSG00000113141 | ENSG00000213346 | 0.95 |
| ENSG00000128731 | ENSG00000213346 | 0.96 |
| ENSG00000136631 | ENSG00000213346 | 0.97 |
| ENSG00000142494 | ENSG00000213346 | -0.96 |
| ENSG00000169136 | ENSG00000213346 | -0.96 |
| ENSG00000072786 | ENSG00000213347 | -0.95 |
| ENSG00000170689 | ENSG00000213347 | 0.97 |
| ENSG00000173207 | ENSG00000213347 | 0.95 |
| ENSG00000175352 | ENSG00000213347 | 0.95 |
| ENSG00000019169 | ENSG00000213398 | 0.95 |
| ENSG00000066813 | ENSG00000213398 | 0.95 |
| ENSG00000072080 | ENSG00000213398 | 0.95 |
| ENSG00000080910 | ENSG00000213398 | 0.96 |
| ENSG00000100652 | ENSG00000213398 | 0.98 |
| ENSG00000102967 | ENSG00000213398 | 0.95 |
| ENSG00000104938 | ENSG00000213398 | 0.97 |
| ENSG00000109758 | ENSG00000213398 | 0.96 |
| ENSG00000112337 | ENSG00000213398 | 0.95 |
| ENSG00000116882 | ENSG00000213398 | 0.95 |
| ENSG00000119965 | ENSG00000213398 | -0.95 |
| ENSG00000122194 | ENSG00000213398 | 0.96 |
| ENSG00000122787 | ENSG00000213398 | 0.97 |
| ENSG00000126759 | ENSG00000213398 | 0.95 |
| ENSG00000134389 | ENSG00000213398 | 0.95 |
| ENSG00000136881 | ENSG00000213398 | 0.95 |
| ENSG00000142494 | ENSG00000213398 | 0.97 |
| ENSG00000149124 | ENSG00000213398 | 0.96 |
| ENSG00000160753 | ENSG00000213398 | -0.95 |
| ENSG00000166035 | ENSG00000213398 | 0.97 |
| ENSG00000172482 | ENSG00000213398 | 0.94 |
| ENSG00000020256 | ENSG00000213445 | -0.96 |
| ENSG00000079257 | ENSG00000213465 | 0.97 |
| ENSG00000171853 | ENSG00000213465 | 0.94 |
| ENSG00000122406 | ENSG00000213579 | 0.96 |
| ENSG00000110244 | ENSG00000213599 | 0.98 |
| ENSG00000117620 | ENSG00000213599 | 0.95 |
| ENSG00000196502 | ENSG00000213599 | 0.96 |
| ENSG00000197165 | ENSG00000213599 | 0.96 |
| ENSG00000081692 | ENSG00000213614 | 0.95 |
| ENSG00000153485 | ENSG00000213672 | -0.95 |
| ENSG00000142156 | ENSG00000213689 | -0.95 |
| ENSG00000159840 | ENSG00000213689 | -0.95 |
| ENSG00000197943 | ENSG00000213693 | -0.94 |
| ENSG00000164344 | ENSG00000213786 | -0.96 |
| ENSG00000198000 | ENSG00000213786 | 0.95 |
| ENSG00000167711 | ENSG00000213846 | -0.95 |
| ENSG00000111110 | ENSG00000213882 | -0.95 |
| ENSG00000100170 | ENSG00000213918 | 0.95 |
| ENSG00000130234 | ENSG00000213918 | 0.96 |
| ENSG00000141434 | ENSG00000213918 | 0.96 |
| ENSG00000179087 | ENSG00000213918 | 0.96 |
| ENSG00000143801 | ENSG00000213930 | 0.96 |
| ENSG00000075142 | ENSG00000213977 | 0.95 |
| ENSG00000101474 | ENSG00000213977 | -0.95 |
| ENSG00000130176 | ENSG00000213977 | 0.95 |
| ENSG00000025156 | ENSG00000213983 | 0.96 |
| ENSG00000072121 | ENSG00000213983 | 0.95 |
| ENSG00000096093 | ENSG00000213983 | 0.95 |
| ENSG00000100802 | ENSG00000213983 | 0.94 |
| ENSG00000102678 | ENSG00000213983 | 0.95 |
| ENSG00000125676 | ENSG00000213983 | 0.94 |
| ENSG00000143409 | ENSG00000213983 | 0.96 |
| ENSG00000197818 | ENSG00000213983 | 0.94 |
| ENSG00000074621 | ENSG00000214021 | 0.96 |
| ENSG00000115421 | ENSG00000214063 | -0.96 |
| ENSG00000125850 | ENSG00000214063 | -0.95 |
| ENSG00000081692 | ENSG00000214078 | 0.94 |
| ENSG00000152092 | ENSG00000214078 | 0.95 |
| ENSG00000173826 | ENSG00000214078 | 0.94 |
| ENSG00000163156 | ENSG00000214167 | -0.95 |
| ENSG00000105011 | ENSG00000214265 | -0.96 |
| ENSG00000182165 | ENSG00000214265 | 0.96 |
| ENSG00000060971 | ENSG00000214274 | 0.96 |
| ENSG00000148218 | ENSG00000214274 | 0.96 |
| ENSG00000106006 | ENSG00000214311 | 0.95 |
| ENSG00000083807 | ENSG00000214313 | 0.97 |
| ENSG00000106327 | ENSG00000214313 | 0.97 |
| ENSG00000137504 | ENSG00000214320 | 0.96 |
| ENSG00000198939 | ENSG00000214367 | 0.95 |
| ENSG00000099326 | ENSG00000214517 | 0.97 |
| ENSG00000104381 | ENSG00000214517 | 0.95 |
| ENSG00000105398 | ENSG00000214517 | -0.95 |
| ENSG00000089902 | ENSG00000214820 | 0.95 |
| ENSG00000096872 | ENSG00000214820 | 0.97 |
| ENSG00000144677 | ENSG00000214820 | 0.95 |
| ENSG00000100802 | ENSG00000214944 | 0.94 |
| ENSG00000138587 | ENSG00000214944 | 0.95 |
| ENSG00000135744 | ENSG00000214973 | -0.96 |
| ENSG00000176619 | ENSG00000214973 | 0.96 |
| ENSG00000197563 | ENSG00000214973 | 0.96 |
| ENSG00000200084 | ENSG00000215030 | 0.98 |
| ENSG00000112029 | ENSG00000215114 | 0.95 |
| ENSG00000108064 | ENSG00000215211 | -0.95 |
| ENSG00000117602 | ENSG00000215211 | 0.95 |
| ENSG00000162552 | ENSG00000215211 | 0.97 |
| ENSG00000136932 | ENSG00000215375 | -0.95 |
| ENSG00000197142 | ENSG00000215859 | 0.94 |
| ENSG00000124678 | ENSG00000216060 | 0.95 |
| ENSG00000198826 | ENSG00000216060 | 0.96 |
| ENSG00000106484 | ENSG00000216657 | 0.96 |
| ENSG00000140199 | ENSG00000216966 | 0.96 |
| ENSG00000211452 | ENSG00000217624 | -0.94 |
| ENSG00000071189 | ENSG00000218089 | 0.97 |
| ENSG00000155833 | ENSG00000218089 | 0.98 |
| ENSG00000185527 | ENSG00000218089 | 0.95 |
| ENSG00000177807 | ENSG00000218890 | 0.95 |
| ENSG00000173114 | ENSG00000219481 | 0.95 |
| ENSG00000178562 | ENSG00000220069 | 0.96 |
| ENSG00000131238 | ENSG00000220205 | 0.95 |
| ENSG00000178982 | ENSG00000221411 | 0.96 |
| ENSG00000119986 | ENSG00000221656 | -0.94 |
| ENSG00000100813 | ENSG00000221716 | 0.95 |
| ENSG00000115963 | ENSG00000221857 | 0.96 |
| ENSG00000139178 | ENSG00000221857 | 0.95 |
| ENSG00000163479 | ENSG00000221857 | -0.96 |
| ENSG00000101180 | ENSG00000221867 | 0.96 |
| ENSG00000174780 | ENSG00000221867 | -0.96 |
| ENSG00000133884 | ENSG00000221870 | -0.94 |
| ENSG00000178104 | ENSG00000221870 | -0.97 |
| ENSG00000013503 | ENSG00000221878 | -0.95 |
| ENSG00000112096 | ENSG00000221886 | -0.97 |
| ENSG00000145882 | ENSG00000221886 | 0.96 |
| ENSG00000161010 | ENSG00000221886 | 0.95 |
| ENSG00000177380 | ENSG00000221886 | 0.97 |
| ENSG00000116698 | ENSG00000221955 | -0.95 |
| ENSG00000104419 | ENSG00000222750 | 0.95 |
| ENSG00000106541 | ENSG00000222750 | 0.97 |
| ENSG00000114529 | ENSG00000222750 | 0.96 |
| ENSG00000129354 | ENSG00000222750 | 0.95 |
| ENSG00000132698 | ENSG00000222750 | 0.98 |
| ENSG00000144908 | ENSG00000222750 | -0.97 |
| ENSG00000189143 | ENSG00000222750 | 0.96 |
| ENSG00000116726 | ENSG00000223513 | 0.95 |
| ENSG00000165806 | ENSG00000223531 | 0.95 |
| ENSG00000138750 | ENSG00000223559 | 0.95 |
| ENSG00000122359 | ENSG00000223572 | 0.94 |
| ENSG00000206066 | ENSG00000223816 | 0.97 |
| ENSG00000172543 | ENSG00000223904 | 0.95 |
| ENSG00000124733 | ENSG00000224126 | 0.95 |
| ENSG00000134853 | ENSG00000224126 | -0.95 |
| ENSG00000165819 | ENSG00000224180 | 0.94 |
| ENSG00000177426 | ENSG00000224180 | 0.95 |
| ENSG00000196712 | ENSG00000224186 | 0.97 |
| ENSG00000152894 | ENSG00000224398 | -0.95 |
| ENSG00000224126 | ENSG00000224398 | 0.95 |
| ENSG00000109586 | ENSG00000224837 | -0.96 |
| ENSG00000151552 | ENSG00000224861 | -0.95 |
| ENSG00000169116 | ENSG00000224861 | -0.96 |
| ENSG00000039537 | ENSG00000224916 | 0.95 |
| ENSG00000066813 | ENSG00000224916 | 0.96 |
| ENSG00000072080 | ENSG00000224916 | 0.96 |
| ENSG00000080910 | ENSG00000224916 | 0.98 |
| ENSG00000100652 | ENSG00000224916 | 0.95 |
| ENSG00000102967 | ENSG00000224916 | 0.96 |
| ENSG00000104760 | ENSG00000224916 | 0.97 |
| ENSG00000104938 | ENSG00000224916 | 0.94 |
| ENSG00000105697 | ENSG00000224916 | 0.95 |
| ENSG00000109758 | ENSG00000224916 | 0.97 |
| ENSG00000110169 | ENSG00000224916 | 0.95 |
| ENSG00000111181 | ENSG00000224916 | 0.96 |
| ENSG00000113600 | ENSG00000224916 | 0.95 |
| ENSG00000113889 | ENSG00000224916 | 0.97 |
| ENSG00000116882 | ENSG00000224916 | 0.95 |
| ENSG00000117594 | ENSG00000224916 | 0.95 |
| ENSG00000117601 | ENSG00000224916 | 0.96 |
| ENSG00000122194 | ENSG00000224916 | 0.96 |
| ENSG00000122787 | ENSG00000224916 | 0.96 |
| ENSG00000123561 | ENSG00000224916 | 0.95 |
| ENSG00000126759 | ENSG00000224916 | 0.97 |
| ENSG00000129988 | ENSG00000224916 | 0.96 |
| ENSG00000130649 | ENSG00000224916 | 0.97 |
| ENSG00000132703 | ENSG00000224916 | 0.96 |
| ENSG00000134365 | ENSG00000224916 | 0.97 |
| ENSG00000134389 | ENSG00000224916 | 0.95 |
| ENSG00000136881 | ENSG00000224916 | 0.95 |
| ENSG00000140505 | ENSG00000224916 | 0.97 |
| ENSG00000142494 | ENSG00000224916 | 0.98 |
| ENSG00000145826 | ENSG00000224916 | 0.96 |
| ENSG00000149124 | ENSG00000224916 | 0.99 |
| ENSG00000151655 | ENSG00000224916 | 0.95 |
| ENSG00000151790 | ENSG00000224916 | 0.98 |
| ENSG00000166035 | ENSG00000224916 | 0.98 |
| ENSG00000188488 | ENSG00000224916 | 0.95 |
| ENSG00000198077 | ENSG00000224916 | 0.95 |
| ENSG00000213398 | ENSG00000224916 | 0.96 |
| ENSG00000126858 | ENSG00000225151 | 0.95 |
| ENSG00000155868 | ENSG00000225151 | 0.95 |
| ENSG00000177469 | ENSG00000225211 | -0.94 |
| ENSG00000090621 | ENSG00000225492 | 0.95 |
| ENSG00000083720 | ENSG00000225531 | 0.95 |
| ENSG00000105835 | ENSG00000225531 | -0.94 |
| ENSG00000106804 | ENSG00000225531 | -0.95 |
| ENSG00000108666 | ENSG00000225531 | 0.95 |
| ENSG00000119640 | ENSG00000225531 | 0.97 |
| ENSG00000168887 | ENSG00000225531 | 0.95 |
| ENSG00000211956 | ENSG00000225698 | 0.96 |
| ENSG00000206208 | ENSG00000225774 | -0.95 |
| ENSG00000110880 | ENSG00000225912 | 0.95 |
| ENSG00000130758 | ENSG00000225980 | 0.95 |
| ENSG00000164654 | ENSG00000225998 | 0.96 |
| ENSG00000100150 | ENSG00000226142 | -0.94 |
| ENSG00000225691 | ENSG00000226201 | 0.95 |
| ENSG00000169981 | ENSG00000226215 | -0.94 |
| ENSG00000183207 | ENSG00000226215 | -0.96 |
| ENSG00000135220 | ENSG00000226306 | 0.96 |
| ENSG00000117115 | ENSG00000226334 | -0.95 |
| ENSG00000206602 | ENSG00000226360 | 0.94 |
| ENSG00000065135 | ENSG00000226565 | -0.96 |
| ENSG00000127946 | ENSG00000226586 | -0.95 |
| ENSG00000165152 | ENSG00000226596 | 0.95 |
| ENSG00000109084 | ENSG00000227051 | -0.97 |
| ENSG00000142677 | ENSG00000227051 | 0.96 |
| ENSG00000136717 | ENSG00000227129 | 0.95 |
| ENSG00000136854 | ENSG00000227242 | 0.95 |
| ENSG00000154978 | ENSG00000227242 | 0.95 |
| ENSG00000204859 | ENSG00000227242 | 0.95 |
| ENSG00000186431 | ENSG00000227246 | 0.95 |
| ENSG00000110514 | ENSG00000227314 | 0.94 |
| ENSG00000115392 | ENSG00000227314 | 0.95 |
| ENSG00000004455 | ENSG00000227372 | -0.95 |
| ENSG00000089053 | ENSG00000227372 | 0.95 |
| ENSG00000165983 | ENSG00000227372 | -0.95 |
| ENSG00000000971 | ENSG00000227429 | -0.95 |
| ENSG00000075240 | ENSG00000227746 | 0.95 |
| ENSG00000079557 | ENSG00000227746 | 0.96 |
| ENSG00000080910 | ENSG00000227746 | 0.95 |
| ENSG00000105697 | ENSG00000227746 | 0.95 |
| ENSG00000110169 | ENSG00000227746 | 0.97 |
| ENSG00000111181 | ENSG00000227746 | 0.95 |
| ENSG00000117594 | ENSG00000227746 | 0.97 |
| ENSG00000140505 | ENSG00000227746 | 0.95 |
| ENSG00000151632 | ENSG00000227746 | 0.95 |
| ENSG00000158104 | ENSG00000227746 | 0.96 |
| ENSG00000158874 | ENSG00000227746 | 0.95 |
| ENSG00000161956 | ENSG00000227746 | -0.95 |
| ENSG00000179271 | ENSG00000227746 | -0.95 |
| ENSG00000196136 | ENSG00000227746 | 0.95 |
| ENSG00000198077 | ENSG00000227746 | 0.96 |
| ENSG00000143420 | ENSG00000227969 | -0.95 |
| ENSG00000092054 | ENSG00000228250 | 0.95 |
| ENSG00000007933 | ENSG00000228278 | 0.97 |
| ENSG00000021852 | ENSG00000228278 | 0.97 |
| ENSG00000080910 | ENSG00000228278 | 0.96 |
| ENSG00000099937 | ENSG00000228278 | 0.95 |
| ENSG00000104760 | ENSG00000228278 | 0.95 |
| ENSG00000106927 | ENSG00000228278 | 0.95 |
| ENSG00000111181 | ENSG00000228278 | 0.95 |
| ENSG00000113600 | ENSG00000228278 | 0.96 |
| ENSG00000113905 | ENSG00000228278 | 0.96 |
| ENSG00000129744 | ENSG00000228278 | 0.94 |
| ENSG00000129988 | ENSG00000228278 | 0.97 |
| ENSG00000130649 | ENSG00000228278 | 0.95 |
| ENSG00000134365 | ENSG00000228278 | 0.95 |
| ENSG00000140505 | ENSG00000228278 | 0.96 |
| ENSG00000142494 | ENSG00000228278 | 0.95 |
| ENSG00000149124 | ENSG00000228278 | 0.97 |
| ENSG00000151655 | ENSG00000228278 | 0.95 |
| ENSG00000151790 | ENSG00000228278 | 0.95 |
| ENSG00000166035 | ENSG00000228278 | 0.95 |
| ENSG00000188488 | ENSG00000228278 | 0.96 |
| ENSG00000224916 | ENSG00000228278 | 0.97 |
| ENSG00000173621 | ENSG00000228581 | 0.95 |
| ENSG00000165424 | ENSG00000228589 | -0.95 |
| ENSG00000179967 | ENSG00000228695 | -0.95 |
| ENSG00000198901 | ENSG00000228695 | -0.94 |
| ENSG00000103540 | ENSG00000228736 | 0.97 |
| ENSG00000108395 | ENSG00000228736 | 0.96 |
| ENSG00000123562 | ENSG00000228736 | 0.95 |
| ENSG00000137073 | ENSG00000228736 | 0.94 |
| ENSG00000137497 | ENSG00000228765 | -0.95 |
| ENSG00000171603 | ENSG00000228765 | -0.95 |
| ENSG00000197408 | ENSG00000228765 | 0.97 |
| ENSG00000069764 | ENSG00000228903 | 0.97 |
| ENSG00000140750 | ENSG00000228903 | 0.95 |
| ENSG00000189093 | ENSG00000228903 | 0.95 |
| ENSG00000197694 | ENSG00000228903 | 0.96 |
| ENSG00000106367 | ENSG00000229048 | -0.94 |
| ENSG00000078795 | ENSG00000229077 | 0.96 |
| ENSG00000117472 | ENSG00000229077 | 0.94 |
| ENSG00000166145 | ENSG00000229077 | 0.98 |
| ENSG00000167699 | ENSG00000229077 | 0.97 |
| ENSG00000173801 | ENSG00000229077 | 0.95 |
| ENSG00000185010 | ENSG00000229077 | -0.95 |
| ENSG00000189143 | ENSG00000229077 | 0.95 |
| ENSG00000174231 | ENSG00000229183 | -0.96 |
| ENSG00000214046 | ENSG00000229183 | -0.96 |
| ENSG00000021852 | ENSG00000229314 | 0.95 |
| ENSG00000075240 | ENSG00000229314 | 0.96 |
| ENSG00000106927 | ENSG00000229314 | 0.96 |
| ENSG00000111181 | ENSG00000229314 | 0.97 |
| ENSG00000113600 | ENSG00000229314 | 0.95 |
| ENSG00000113905 | ENSG00000229314 | 0.95 |
| ENSG00000119401 | ENSG00000229314 | -0.97 |
| ENSG00000129988 | ENSG00000229314 | 0.95 |
| ENSG00000132693 | ENSG00000229314 | 0.98 |
| ENSG00000132703 | ENSG00000229314 | 0.96 |
| ENSG00000151655 | ENSG00000229314 | 0.96 |
| ENSG00000158874 | ENSG00000229314 | 0.95 |
| ENSG00000171560 | ENSG00000229314 | 0.95 |
| ENSG00000175189 | ENSG00000229314 | 0.94 |
| ENSG00000196136 | ENSG00000229314 | 0.95 |
| ENSG00000228278 | ENSG00000229314 | 0.96 |
| ENSG00000134825 | ENSG00000229363 | 0.95 |
| ENSG00000158042 | ENSG00000229363 | 0.95 |
| ENSG00000124225 | ENSG00000229638 | 0.95 |
| ENSG00000204196 | ENSG00000229638 | 0.98 |
| ENSG00000164823 | ENSG00000229676 | 0.94 |
| ENSG00000187608 | ENSG00000229684 | 0.95 |
| ENSG00000102935 | ENSG00000229861 | -0.95 |
| ENSG00000125089 | ENSG00000229932 | -0.99 |
| ENSG00000085832 | ENSG00000229944 | 0.95 |
| ENSG00000140199 | ENSG00000229988 | 0.95 |
| ENSG00000141378 | ENSG00000230199 | 0.95 |
| ENSG00000138814 | ENSG00000230259 | -0.94 |
| ENSG00000092098 | ENSG00000230373 | 0.95 |
| ENSG00000109472 | ENSG00000230606 | 0.95 |
| ENSG00000123562 | ENSG00000230606 | 0.95 |
| ENSG00000140939 | ENSG00000230606 | 0.95 |
| ENSG00000167972 | ENSG00000230606 | 0.95 |
| ENSG00000178252 | ENSG00000230606 | 0.95 |
| ENSG00000151846 | ENSG00000230673 | 0.95 |
| ENSG00000100330 | ENSG00000230705 | -0.95 |
| ENSG00000188229 | ENSG00000230705 | 0.95 |
| ENSG00000122121 | ENSG00000230712 | 0.96 |
| ENSG00000130055 | ENSG00000230712 | 0.95 |
| ENSG00000133475 | ENSG00000230712 | 0.95 |
| ENSG00000141434 | ENSG00000230712 | 0.95 |
| ENSG00000172238 | ENSG00000230712 | 0.95 |
| ENSG00000019582 | ENSG00000230726 | 0.96 |
| ENSG00000165458 | ENSG00000230888 | 0.95 |
| ENSG00000120471 | ENSG00000231286 | -0.96 |
| ENSG00000206066 | ENSG00000231292 | 0.97 |
| ENSG00000211598 | ENSG00000231292 | 0.94 |
| ENSG00000211934 | ENSG00000231292 | 0.95 |
| ENSG00000106524 | ENSG00000231397 | 0.95 |
| ENSG00000154723 | ENSG00000231584 | 0.94 |
| ENSG00000014824 | ENSG00000231700 | 0.97 |
| ENSG00000075539 | ENSG00000231700 | 0.95 |
| ENSG00000117758 | ENSG00000231700 | 0.95 |
| ENSG00000127914 | ENSG00000231700 | 0.95 |
| ENSG00000124766 | ENSG00000231726 | 0.98 |
| ENSG00000126814 | ENSG00000231726 | 0.96 |
| ENSG00000184113 | ENSG00000231900 | 0.95 |
| ENSG00000008283 | ENSG00000232561 | 0.95 |
| ENSG00000101150 | ENSG00000232561 | 0.96 |
| ENSG00000163814 | ENSG00000232561 | 0.95 |
| ENSG00000164879 | ENSG00000232573 | -0.95 |
| ENSG00000209480 | ENSG00000232573 | 0.96 |
| ENSG00000073464 | ENSG00000232691 | 0.95 |
| ENSG00000205084 | ENSG00000232691 | 0.95 |
| ENSG00000072121 | ENSG00000232931 | 0.95 |
| ENSG00000117791 | ENSG00000232931 | -0.94 |
| ENSG00000167965 | ENSG00000232931 | 0.95 |
| ENSG00000171606 | ENSG00000232931 | -0.97 |
| ENSG00000177380 | ENSG00000232931 | 0.95 |
| ENSG00000197102 | ENSG00000232931 | 0.95 |
| ENSG00000091972 | ENSG00000233297 | 0.95 |
| ENSG00000100393 | ENSG00000233297 | 0.97 |
| ENSG00000140750 | ENSG00000233297 | 0.95 |
| ENSG00000204843 | ENSG00000233297 | 0.95 |
| ENSG00000056998 | ENSG00000233401 | 0.95 |
| ENSG00000207740 | ENSG00000233402 | 0.96 |
| ENSG00000124789 | ENSG00000233564 | 0.95 |
| ENSG00000232176 | ENSG00000233762 | 0.95 |
| ENSG00000130695 | ENSG00000234032 | 0.95 |
| ENSG00000153558 | ENSG00000234305 | -0.95 |
| ENSG00000211679 | ENSG00000234353 | 0.95 |
| ENSG00000175029 | ENSG00000234383 | 0.96 |
| ENSG00000179456 | ENSG00000234383 | 0.96 |
| ENSG00000077782 | ENSG00000234651 | 0.94 |
| ENSG00000105379 | ENSG00000234651 | -0.96 |
| ENSG00000170430 | ENSG00000234651 | -0.95 |
| ENSG00000224126 | ENSG00000234651 | 0.95 |
| ENSG00000112096 | ENSG00000234906 | 0.95 |
| ENSG00000136193 | ENSG00000234906 | -0.96 |
| ENSG00000185928 | ENSG00000234906 | -0.95 |
| ENSG00000198218 | ENSG00000234906 | -0.96 |
| ENSG00000221886 | ENSG00000234906 | -0.96 |
| ENSG00000055118 | ENSG00000235106 | 0.97 |
| ENSG00000114956 | ENSG00000235106 | 0.95 |
| ENSG00000114988 | ENSG00000235106 | 0.94 |
| ENSG00000174938 | ENSG00000235106 | 0.96 |
| ENSG00000183741 | ENSG00000235106 | 0.96 |
| ENSG00000147454 | ENSG00000235125 | -0.95 |
| ENSG00000163554 | ENSG00000235449 | -0.95 |
| ENSG00000129351 | ENSG00000235734 | 0.95 |
| ENSG00000066455 | ENSG00000235859 | 0.95 |
| ENSG00000188021 | ENSG00000235859 | 0.95 |
| ENSG00000204256 | ENSG00000236227 | 0.97 |
| ENSG00000167656 | ENSG00000236256 | 0.96 |
| ENSG00000008838 | ENSG00000236263 | 0.97 |
| ENSG00000077254 | ENSG00000236263 | 0.95 |
| ENSG00000171450 | ENSG00000236263 | 0.96 |
| ENSG00000176894 | ENSG00000236263 | -0.96 |
| ENSG00000168090 | ENSG00000236264 | 0.94 |
| ENSG00000035720 | ENSG00000236342 | 0.94 |
| ENSG00000207051 | ENSG00000236439 | 0.95 |
| ENSG00000179841 | ENSG00000236524 | 0.96 |
| ENSG00000199631 | ENSG00000236552 | 0.95 |
| ENSG00000232856 | ENSG00000236552 | 0.95 |
| ENSG00000019169 | ENSG00000236613 | 0.95 |
| ENSG00000072080 | ENSG00000236613 | 0.98 |
| ENSG00000080910 | ENSG00000236613 | 0.95 |
| ENSG00000100652 | ENSG00000236613 | 0.96 |
| ENSG00000101981 | ENSG00000236613 | 0.94 |
| ENSG00000104760 | ENSG00000236613 | 0.95 |
| ENSG00000104915 | ENSG00000236613 | -0.96 |
| ENSG00000104938 | ENSG00000236613 | 0.95 |
| ENSG00000112337 | ENSG00000236613 | 0.94 |
| ENSG00000116882 | ENSG00000236613 | 0.96 |
| ENSG00000122194 | ENSG00000236613 | 0.96 |
| ENSG00000122787 | ENSG00000236613 | 0.97 |
| ENSG00000130649 | ENSG00000236613 | 0.95 |
| ENSG00000132703 | ENSG00000236613 | 0.95 |
| ENSG00000134389 | ENSG00000236613 | 0.95 |
| ENSG00000135094 | ENSG00000236613 | 0.96 |
| ENSG00000136881 | ENSG00000236613 | 0.96 |
| ENSG00000142494 | ENSG00000236613 | 0.96 |
| ENSG00000149124 | ENSG00000236613 | 0.96 |
| ENSG00000156642 | ENSG00000236613 | -0.95 |
| ENSG00000166035 | ENSG00000236613 | 0.98 |
| ENSG00000178950 | ENSG00000236613 | -0.97 |
| ENSG00000213398 | ENSG00000236613 | 0.96 |
| ENSG00000224916 | ENSG00000236613 | 0.97 |
| ENSG00000135951 | ENSG00000236698 | -0.96 |
| ENSG00000163666 | ENSG00000236772 | -0.96 |
| ENSG00000174977 | ENSG00000237056 | 0.95 |
| ENSG00000176014 | ENSG00000237095 | -0.95 |
| ENSG00000147488 | ENSG00000237125 | -0.95 |
| ENSG00000080345 | ENSG00000237649 | 0.96 |
| ENSG00000099785 | ENSG00000237649 | -0.94 |
| ENSG00000178287 | ENSG00000237649 | 0.96 |
| ENSG00000231618 | ENSG00000237758 | 0.94 |
| ENSG00000199293 | ENSG00000239344 | 0.95 |
| ENSG00000198910 | ENSG00000239713 | -0.96 |
| ENSG00000206066 | ENSG00000239951 | 0.95 |
| ENSG00000211598 | ENSG00000239951 | 0.96 |
| ENSG00000211630 | ENSG00000239951 | 0.97 |
| ENSG00000071539 | ENSG00000240122 | 0.96 |
| ENSG00000164611 | ENSG00000240122 | 0.94 |
| ENSG00000060558 | ENSG00000240230 | -0.95 |
| ENSG00000133216 | ENSG00000240694 | -0.95 |
| ENSG00000105372 | ENSG00000240914 | 0.95 |
| ENSG00000106927 | ENSG00000240935 | 0.95 |
| ENSG00000110887 | ENSG00000240935 | 0.95 |
| ENSG00000113905 | ENSG00000240935 | 0.96 |
| ENSG00000119401 | ENSG00000240935 | -0.95 |
| ENSG00000129988 | ENSG00000240935 | 0.95 |
| ENSG00000132693 | ENSG00000240935 | 0.95 |
| ENSG00000132703 | ENSG00000240935 | 0.95 |
| ENSG00000135094 | ENSG00000240935 | 0.96 |
| ENSG00000139547 | ENSG00000240935 | 0.96 |
| ENSG00000148702 | ENSG00000240935 | 0.96 |
| ENSG00000151655 | ENSG00000240935 | 0.95 |
| ENSG00000188488 | ENSG00000240935 | 0.95 |
| ENSG00000229314 | ENSG00000240935 | 0.95 |
| ENSG00000116704 | ENSG00000240972 | -0.96 |
| ENSG00000138109 | ENSG00000240972 | -0.96 |
| ENSG00000160868 | ENSG00000241119 | 0.96 |
| ENSG00000115474 | ENSG00000241409 | 0.96 |
| ENSG00000130234 | ENSG00000241409 | 0.95 |
| ENSG00000141434 | ENSG00000241409 | 0.95 |
| ENSG00000166869 | ENSG00000241409 | 0.94 |
| ENSG00000179087 | ENSG00000241409 | 0.95 |
| ENSG00000197273 | ENSG00000241409 | 0.94 |
| ENSG00000096093 | ENSG00000241434 | 0.96 |
| ENSG00000114956 | ENSG00000241434 | 0.97 |
| ENSG00000115896 | ENSG00000241434 | 0.94 |
| ENSG00000116128 | ENSG00000241434 | 0.94 |
| ENSG00000183741 | ENSG00000241434 | 0.94 |
| ENSG00000116171 | ENSG00000241635 | 0.96 |
| ENSG00000148672 | ENSG00000241635 | 0.95 |
| ENSG00000205358 | ENSG00000241635 | 0.96 |
| ENSG00000240972 | ENSG00000241635 | -0.96 |
| ENSG00000070748 | ENSG00000241728 | -0.95 |
| ENSG00000170099 | ENSG00000241779 | -0.98 |
| ENSG00000119638 | ENSG00000241850 | -0.95 |
| ENSG00000157782 | ENSG00000242019 | 0.95 |
| ENSG00000165671 | ENSG00000242125 | 0.95 |
| ENSG00000020426 | ENSG00000242140 | 0.97 |
| ENSG00000004487 | ENSG00000242173 | -0.96 |
| ENSG00000105976 | ENSG00000242173 | 0.94 |
| ENSG00000109667 | ENSG00000242173 | 0.96 |
| ENSG00000128656 | ENSG00000242173 | -0.96 |
| ENSG00000137497 | ENSG00000242173 | -0.95 |
| ENSG00000149179 | ENSG00000242173 | -0.95 |
| ENSG00000168000 | ENSG00000242173 | -0.96 |
| ENSG00000171603 | ENSG00000242173 | -0.96 |
| ENSG00000198646 | ENSG00000242173 | -0.95 |
| ENSG00000205629 | ENSG00000242173 | -0.97 |
| ENSG00000172869 | ENSG00000242534 | -0.96 |
| ENSG00000206066 | ENSG00000242534 | 0.97 |
| ENSG00000207836 | ENSG00000242534 | 0.95 |
| ENSG00000211598 | ENSG00000242534 | 0.98 |
| ENSG00000211630 | ENSG00000242534 | 0.96 |
| ENSG00000211679 | ENSG00000242534 | 0.95 |
| ENSG00000211934 | ENSG00000242534 | 0.96 |
| ENSG00000211940 | ENSG00000242534 | 0.96 |
| ENSG00000223816 | ENSG00000242534 | 0.95 |
| ENSG00000231292 | ENSG00000242534 | 0.97 |
| ENSG00000239951 | ENSG00000242534 | 0.97 |
| ENSG00000102048 | ENSG00000242550 | 0.96 |
| ENSG00000167910 | ENSG00000242550 | 0.94 |
| ENSG00000103502 | ENSG00000242616 | 0.95 |
| ENSG00000199744 | ENSG00000242735 | 0.96 |
| ENSG00000004468 | ENSG00000242766 | 0.96 |
| ENSG00000206066 | ENSG00000242766 | 0.95 |
| ENSG00000207836 | ENSG00000242766 | 0.96 |
| ENSG00000211598 | ENSG00000242766 | 0.95 |
| ENSG00000211630 | ENSG00000242766 | 0.98 |
| ENSG00000211679 | ENSG00000242766 | 0.97 |
| ENSG00000211934 | ENSG00000242766 | 0.97 |
| ENSG00000231292 | ENSG00000242766 | 0.95 |
| ENSG00000239951 | ENSG00000242766 | 0.97 |
| ENSG00000242534 | ENSG00000242766 | 0.97 |
| ENSG00000100142 | ENSG00000243056 | 0.94 |
| ENSG00000136040 | ENSG00000243232 | 0.96 |
| ENSG00000148798 | ENSG00000243232 | 0.95 |
| ENSG00000172348 | ENSG00000243232 | 0.96 |
| ENSG00000185324 | ENSG00000243232 | 0.95 |
| ENSG00000196405 | ENSG00000243232 | 0.95 |
| ENSG00000143384 | ENSG00000243335 | -0.95 |
| ENSG00000211898 | ENSG00000243477 | -0.94 |
| ENSG00000126012 | ENSG00000243554 | 0.94 |
| ENSG00000050393 | ENSG00000243646 | 0.96 |
| ENSG00000113108 | ENSG00000243646 | -0.95 |
| ENSG00000007933 | ENSG00000243696 | 0.95 |
| ENSG00000019169 | ENSG00000243696 | 0.96 |
| ENSG00000072080 | ENSG00000243696 | 0.95 |
| ENSG00000080910 | ENSG00000243696 | 0.98 |
| ENSG00000099937 | ENSG00000243696 | 0.95 |
| ENSG00000100652 | ENSG00000243696 | 0.95 |
| ENSG00000102967 | ENSG00000243696 | 0.95 |
| ENSG00000104760 | ENSG00000243696 | 0.97 |
| ENSG00000104938 | ENSG00000243696 | 0.96 |
| ENSG00000105697 | ENSG00000243696 | 0.95 |
| ENSG00000109758 | ENSG00000243696 | 0.98 |
| ENSG00000110169 | ENSG00000243696 | 0.97 |
| ENSG00000111181 | ENSG00000243696 | 0.95 |
| ENSG00000113905 | ENSG00000243696 | 0.96 |
| ENSG00000117594 | ENSG00000243696 | 0.96 |
| ENSG00000117601 | ENSG00000243696 | 0.96 |
| ENSG00000122194 | ENSG00000243696 | 0.96 |
| ENSG00000122787 | ENSG00000243696 | 0.96 |
| ENSG00000123561 | ENSG00000243696 | 0.95 |
| ENSG00000126759 | ENSG00000243696 | 0.96 |
| ENSG00000129988 | ENSG00000243696 | 0.97 |
| ENSG00000130649 | ENSG00000243696 | 0.97 |
| ENSG00000134365 | ENSG00000243696 | 0.95 |
| ENSG00000134389 | ENSG00000243696 | 0.95 |
| ENSG00000139547 | ENSG00000243696 | 0.95 |
| ENSG00000140505 | ENSG00000243696 | 0.98 |
| ENSG00000142494 | ENSG00000243696 | 0.97 |
| ENSG00000145826 | ENSG00000243696 | 0.96 |
| ENSG00000149124 | ENSG00000243696 | 0.98 |
| ENSG00000151790 | ENSG00000243696 | 0.96 |
| ENSG00000158104 | ENSG00000243696 | 0.95 |
| ENSG00000166035 | ENSG00000243696 | 0.97 |
| ENSG00000180210 | ENSG00000243696 | 0.95 |
| ENSG00000188488 | ENSG00000243696 | 0.95 |
| ENSG00000198077 | ENSG00000243696 | 0.97 |
| ENSG00000213398 | ENSG00000243696 | 0.96 |
| ENSG00000224916 | ENSG00000243696 | 0.98 |
| ENSG00000228278 | ENSG00000243696 | 0.97 |
| ENSG00000143776 | ENSG00000243716 | 0.95 |
| ENSG00000106327 | ENSG00000243754 | 0.95 |
| ENSG00000145192 | ENSG00000243754 | 0.94 |
| ENSG00000172830 | ENSG00000243754 | -0.99 |
| ENSG00000173432 | ENSG00000243754 | 0.96 |
| ENSG00000086696 | ENSG00000243955 | 0.96 |
| ENSG00000116771 | ENSG00000243955 | 0.95 |
| ENSG00000136872 | ENSG00000243955 | 0.95 |
| ENSG00000156006 | ENSG00000243955 | 0.95 |
| ENSG00000106688 | ENSG00000243989 | 0.95 |
| ENSG00000140374 | ENSG00000243989 | 0.95 |
| ENSG00000156006 | ENSG00000243989 | 0.97 |
| ENSG00000172831 | ENSG00000243989 | 0.97 |
| ENSG00000173221 | ENSG00000243989 | 0.97 |
| ENSG00000178537 | ENSG00000243989 | 0.96 |
| ENSG00000196502 | ENSG00000243989 | 0.96 |
| ENSG00000197165 | ENSG00000243989 | 0.96 |
| ENSG00000075234 | ENSG00000244020 | 0.97 |
| ENSG00000162521 | ENSG00000244020 | -0.94 |
| ENSG00000169715 | ENSG00000244020 | 0.97 |
| ENSG00000187193 | ENSG00000244020 | 0.94 |
| ENSG00000198417 | ENSG00000244020 | 0.95 |
| ENSG00000205358 | ENSG00000244020 | 0.94 |
| ENSG00000084764 | ENSG00000244179 | 0.95 |
| ENSG00000149182 | ENSG00000244179 | 0.96 |
| ENSG00000165731 | ENSG00000244179 | 0.94 |
| ENSG00000105699 | ENSG00000244202 | 0.96 |
| ENSG00000187239 | ENSG00000244202 | -0.95 |
| ENSG00000239344 | ENSG00000244313 | 0.96 |
| ENSG00000226085 | ENSG00000244385 | 0.94 |
| ENSG00000081041 | ENSG00000244414 | 0.95 |
| ENSG00000154262 | ENSG00000244414 | 0.95 |
| ENSG00000084731 | ENSG00000244462 | 0.95 |
| ENSG00000089091 | ENSG00000244462 | 0.96 |
| ENSG00000105372 | ENSG00000244716 | 0.97 |
| ENSG00000168291 | ENSG00000244734 | -0.95 |
| ENSG00000206172 | ENSG00000244734 | 0.98 |
| ENSG00000138092 | ENSG00000244752 | 0.95 |
| ENSG00000156052 | ENSG00000245773 | 0.95 |
| ENSG00000163541 | ENSG00000246275 | 0.95 |
| ENSG00000185672 | ENSG00000246275 | -0.96 |
| ENSG00000100012 | ENSG00000247396 | 0.95 |
| ENSG00000147432 | ENSG00000247396 | 0.94 |
| ENSG00000166012 | ENSG00000247909 | 0.96 |
| ENSG00000136381 | ENSG00000248098 | 0.95 |
| ENSG00000224105 | ENSG00000248098 | -0.95 |
| ENSG00000215021 | ENSG00000248175 | -0.95 |
| ENSG00000100564 | ENSG00000248333 | 0.94 |
| ENSG00000182795 | ENSG00000248413 | 0.95 |
| ENSG00000106628 | ENSG00000248535 | 0.96 |
| ENSG00000162711 | ENSG00000248536 | 0.98 |
| ENSG00000166997 | ENSG00000248536 | 0.96 |
| ENSG00000019485 | ENSG00000248675 | 0.94 |
| ENSG00000143512 | ENSG00000248691 | 0.95 |
| ENSG00000235698 | ENSG00000248833 | 0.96 |
| ENSG00000092096 | ENSG00000249037 | 0.95 |
| ENSG00000117090 | ENSG00000249037 | -0.96 |
| ENSG00000151208 | ENSG00000249037 | 0.95 |
| ENSG00000196090 | ENSG00000249037 | 0.95 |
| ENSG00000147408 | ENSG00000249268 | -0.95 |
| ENSG00000112578 | ENSG00000249281 | 0.95 |
| ENSG00000120280 | ENSG00000249350 | 0.97 |
| ENSG00000006638 | ENSG00000249544 | 0.95 |
| ENSG00000164050 | ENSG00000249611 | -0.97 |
| ENSG00000164002 | ENSG00000249781 | 0.95 |
| ENSG00000165943 | ENSG00000249793 | 0.97 |
| ENSG00000176956 | ENSG00000249793 | 0.98 |
| ENSG00000116830 | ENSG00000249905 | 0.97 |
| ENSG00000070501 | ENSG00000249948 | -0.96 |
| ENSG00000099834 | ENSG00000249948 | 0.95 |
| ENSG00000197635 | ENSG00000249948 | 0.96 |
| ENSG00000176454 | ENSG00000249953 | -0.95 |
| ENSG00000126218 | ENSG00000249956 | 0.95 |
| ENSG00000135437 | ENSG00000249956 | 0.96 |
| ENSG00000100387 | ENSG00000250011 | -0.95 |
| ENSG00000206066 | ENSG00000250036 | 0.97 |
| ENSG00000211598 | ENSG00000250036 | 0.95 |
| ENSG00000211970 | ENSG00000250036 | 0.95 |
| ENSG00000223816 | ENSG00000250036 | 0.96 |
| ENSG00000231292 | ENSG00000250036 | 0.96 |
| ENSG00000239951 | ENSG00000250036 | 0.95 |
| ENSG00000242534 | ENSG00000250036 | 0.97 |
| ENSG00000135336 | ENSG00000250096 | -0.95 |
| ENSG00000106052 | ENSG00000250151 | 0.95 |
| ENSG00000132702 | ENSG00000250287 | -0.95 |
| ENSG00000197757 | ENSG00000250320 | -0.94 |
| ENSG00000087077 | ENSG00000250361 | -0.94 |
| ENSG00000115421 | ENSG00000250361 | 0.95 |
| ENSG00000196159 | ENSG00000250361 | -0.95 |
| ENSG00000137673 | ENSG00000250460 | 0.95 |
| ENSG00000233734 | ENSG00000250460 | 0.95 |
| ENSG00000142534 | ENSG00000250536 | 0.94 |
| ENSG00000250591 | ENSG00000250606 | 0.95 |
| ENSG00000129003 | ENSG00000250653 | 0.97 |
| ENSG00000132466 | ENSG00000250675 | 0.94 |
| ENSG00000103154 | ENSG00000250799 | 0.95 |
| ENSG00000151552 | ENSG00000250799 | 0.96 |
| ENSG00000165801 | ENSG00000250799 | 0.96 |
| ENSG00000166501 | ENSG00000250845 | -0.96 |
| ENSG00000102241 | ENSG00000250896 | 0.96 |
| ENSG00000111602 | ENSG00000250896 | 0.95 |
| ENSG00000143248 | ENSG00000250898 | 0.96 |
| ENSG00000133935 | ENSG00000250996 | -0.95 |
| ENSG00000100453 | ENSG00000251020 | 0.95 |
| ENSG00000075303 | ENSG00000251023 | 0.96 |
| ENSG00000117592 | ENSG00000251102 | -0.96 |
| ENSG00000151690 | ENSG00000251102 | 0.95 |
| ENSG00000174607 | ENSG00000251400 | -0.95 |
| ENSG00000172869 | ENSG00000251546 | -0.95 |
| ENSG00000211592 | ENSG00000251546 | 0.97 |
| ENSG00000211598 | ENSG00000251546 | 0.95 |
| ENSG00000211630 | ENSG00000251546 | 0.97 |
| ENSG00000211659 | ENSG00000251546 | 0.95 |
| ENSG00000211679 | ENSG00000251546 | 0.98 |
| ENSG00000211934 | ENSG00000251546 | 0.95 |
| ENSG00000239951 | ENSG00000251546 | 0.97 |
| ENSG00000242534 | ENSG00000251546 | 0.96 |
| ENSG00000242766 | ENSG00000251546 | 0.98 |
